# Carbon-based materials for the removal of organic dyes from wastewater

**DOI:** 10.1186/s11671-026-04445-5

**Published:** 2026-02-05

**Authors:** Bernice Yram Danu, Charles Kwame Bandoh, John Kwabena Adusei, Moro Haruna, Ahmed Kangmennaa, Prince Yeboah, Francis Kofi Ampong, Eric Selorm Agorku

**Affiliations:** 1 Department of Chemical Sciences, University of Energy and Natural Resources, P. O. Box 214, Sunyani, Ghana; 2 Department of Physics, Kwame Nkrumah University of Science and Technology, Kumasi, Ghana; 3 Department of Chemistry, Kwame Nkrumah University of Science and Technology, Kumasi, Ghana; 4 Department of Chemical Sciences, University for Development Studies, Tamale, Ghana

**Keywords:** Adsorption, Photocatalysis, Carbon-based materials, Dyes, Wastewater

## Abstract

Carbon-based nanomaterials have garnered significant interest as efficient adsorbents for removing organic dyes from wastewater due to their unique physicochemical properties. Carbon nanotubes, graphite, graphene, and activated carbon are among the most studied carbon-based nanomaterials, owing to their large surface areas and high adsorption capacities. These nanoparticles’ surface functionalization and modification can improve their adsorption capabilities, allowing for the selective removal of dyes from complicated wastewater matrices. Several synthesis approaches have been used to modify the characteristics of carbon-based nanomaterials to address specific dye removal needs. The usage of carbon-based nanomaterials for dye removal yields favourable results, providing a cost-effective, environmentally friendly, and long-term solution to wastewater treatment. Nonetheless, scale-up, regeneration, and long-term stability issues must be overcome to facilitate industrial-scale adoption. Despite significant advantages, including high adsorption capacity, photodegradation efficiency, reusability, and environmental compatibility, challenges persist in industrial implementation. Production costs, scalability limitations, and economic viability constraints hinder large-scale adoption. Synthesis methods require optimization for cost-effectiveness while maintaining treatment efficiency. Future research should prioritize developing economical synthesis routes, optimizing material properties for specific applications, and establishing standardized evaluation protocols. The integration of waste-derived precursors offers promising opportunities for sustainable treatment solutions. This review provides a comprehensive framework for understanding current capabilities and future directions in carbon-based wastewater treatment, emphasizing both the substantial potential and existing challenges that must be addressed for successful industrial implementation.

**Graphical abstract:**

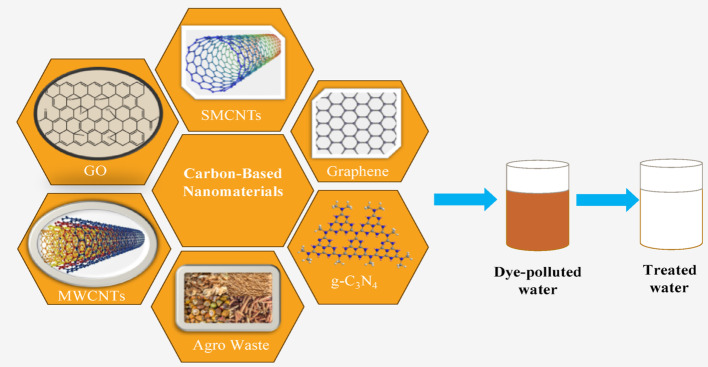

## Introduction

Water pollution caused by organic dyes represents one of the most tenacious environmental challenges of the 21st century. Organic dyes can be broadly classified based on their chemical structure (e.g., azo, anthraquinone, triphenylmethane, phthalocyanine, and indigo dyes) or their application method (e.g., reactive, direct, acid, basic, disperse, and vat dyes) [1]. Azo dyes, characterized by one or more azo bonds (-N = N-), constitute approximately 60–70% of all commercial dyes due to their versatility, colour brightness, and cost-effectiveness [2]. These dyes originate primarily from textile manufacturing, but also from paper, leather tanning, food processing, pharmaceutical, and cosmetic industries, with approximately 280,000 tons of dye-laden wastewater released globally each year [3]. More disturbingly, between 10 and 15% of dyes used in industrial processes remain unreacted during application and find their way into effluents. The situation is particularly terrible in developing nations, where up to 80% of dye-containing wastewater is discharged without suitable post-treatment solutions [4]. The environmental persistence and toxicity of organic dyes stem from their complex aromatic structures, which are specifically designed to resist degradation from light, water, chemicals, and microbial attack-properties that make them excellent colorants but problematic pollutants [5]. Furthermore, the presence of chromophoric groups (responsible for colour) and auxochrome groups (which enhance colour intensity and dye-fiber binding) contributes to their recalcitrance in aquatic environments and their tendency to bioaccumulate in living organisms [6]. Understanding these structural and behavioral characteristics is crucial for developing targeted removal strategies, as different dye classes respond differently to various treatment approaches based on their molecular weight, charge, solubility, and functional groups.

Dye pollution has significant and wide-ranging effects on the environment and human health. In aquatic environments, organic dyes reduce light penetration, raise chemical and biochemical oxygen demand (BOD and COD), and seriously degrade the aesthetic quality of water bodies [3]. Through bioaccumulation in the food chain, numerous synthetic dyes and their metabolites pose major risks to aquatic life and human health due to their poisonous, carcinogenic, mutagenic, or teratogenic qualities [7]. Additionally, many commercial dyes are resistant to traditional wastewater treatment techniques due to their complex molecular structures, which results in persistent environmental pollutants. Despite being locally confined, this issue is of global importance. The textile industry’s extensive water usage drives its contribution of over 80% of dye effluent contamination in Africa’s surface waters [8]. China, India, and Bangladesh collectively release over 3.5 billion tons of textile effluent annually [4]. According to reports by Islam et al. [9]. , textile dyeing and fabric finishing processes are responsible for 17–20% of industrial water contamination, revealing a complex environmental concern.

To address these issues, regulatory agencies around the world have set strict guidelines for the amount of dye in treated effluent. For wastewater released into surface waters and municipal sewage treatment facilities, the U.S. Environmental Protection Agency (EPA) has established nationwide regulatory criteria [10]. Similar policies have been implemented in a variety of nations with varying levels of severity. According to certain laws, the actual color discharge limit is 400 American Dye Manufacturers Institute (ADMI) units [11]. The pH is normally regulated within a range of 6–9 units, and the COD is normally regulated between 8 and 250 mg/L [12]. Depending on the particular dyeing business, the quantity of dye in industrial effluent might amount to 7000 mg/L, although it usually falls between 10 and 200 mg/L [13]. These concentrations greatly surpass the legal thresholds, highlighting the necessity of efficient treatment methods before release.

Several wastewater remediation techniques have been employed over the years, which include: adsorption, photocatalysis, precipitation, ion exchange, membrane separation, filtration, chemical oxidation, and biological treatment [14, 15]. Among these various remediation approaches, adsorption and photocatalysis using carbon-based materials (CBMs) have emerged as particularly promising techniques for dye removal, owing to their high efficiency, cost-effectiveness, and environmental compatibility. Carbon-based materials such as graphene and its derivatives, carbon nanotubes (CNTs), activated carbon, and other agricultural-waste-derived materials exhibit exceptional performance attributed to their large specific surface area, abundant functional groups, high chemical stability, and versatile modification potential [16]. In wastewater treatment, CBMs use various mechanisms to trap dyes, including hydrophobic interactions, ionic bonding, and π-π stacking, which facilitate the binding of dye molecules to the surface of the CBMs. The efficiency often surpasses 90%, making CBMs a preferred choice over other materials [17]. Recent advancements in research have focused on enhancing the performance of these materials through modifications and innovative applications. Techniques such as activating carbon with different chemical agents or incorporating nanoparticles have shown promising results in increasing dye removal capacities. Additionally, developments in the production of biochar and other bio-waste-derived materials have highlighted the potential for using agricultural byproducts, thus promoting sustainability.

Furthermore, the integration of these innovative adsorbents plays a crucial role in various environmental applications, including wastewater treatment and soil enhancement. This strategic use of biomass-derived materials, particularly in adsorptive capacities, showcases their significant contributions towards alleviating pollution and fostering sustainability. Overall, the ongoing research and application of CBMs for dye removal not only offer a practical solution to wastewater treatment but also align with efforts to create a more sustainable environment. By harnessing these materials, industries can significantly reduce dye pollution while making efficient use of available resources. The integration of research findings into practical applications paves the way for more effective and eco-friendly wastewater management strategies.

CBMs can be significantly enhanced for dye removal efficiency through strategic chemical modifications and the application of nanotechnology. Chemical treatments, such as activation with sodium hydroxide (NaOH) or acid-based processes, introduce oxygen- and nitrogen-containing functional groups on the carbon surface, which increase its affinity for ionic and polar dye molecules [18]. These functional groups improve surface polarity and create active binding sites, thereby enhancing adsorption capacity. Nanotechnology further amplifies performance by increasing the specific surface area, creating nanostructured active sites, and increasing reactivity [19]. For instance, incorporating iron or iron oxide nanoparticles into carbon matrices enables magnetic separation and enhances surface reactivity [20]. Similarly, the use of carbon nanotubes (CNTs) and graphene-based nanocomposites provides high surface-to-volume ratios and tunable surface chemistry, making them highly effective in adsorbing and degrading dyes [21]. Additionally, emerging nanomaterials like carbon nanodots are being explored for their dual role in adsorption and photocatalytic degradation of dye pollutants, offering a novel approach to effluent remediation [22].

Recent innovations in this field emphasize sustainability by utilizing agricultural waste as a precursor for carbon-based adsorbents. Materials such as rice husks, coconut shells, sugarcane bagasse, cocoa residues, and banana peels are being upcycled into activated carbons and nano-activated composites, thereby transforming waste into high-value environmental remediation tools [23]. These bio-derived carbons not only reduce dependency on fossil-based precursors but also support the principles of a circular economy. Agro-waste-derived nano-activated carbons and nanocomposites have demonstrated superior adsorption performance compared to conventional materials, which is attributed to their porous structure and modifiable surface chemistry [24]. The integration of such sustainable feedstocks with nanotechnology, such as producing nanocomposites or functionalizing surfaces at the nanoscale, has led to materials that are both eco-friendly and highly efficient [25]. This trend reflects a growing shift toward sustainable, low-cost, and scalable wastewater treatment solutions, aligning environmental protection with resource efficiency in water purification technologies [24, 26].

Despite the advances, several challenges persist in the application of CBMs for dye removal. These include mass transfer limitations, selectivity issues in complex wastewater matrices, nanomaterials regeneration difficulties, and economic feasibility for large-scale applications [16]. Additionally, the adsorption capacity of most adsorbents remains relatively low compared to the high concentrations of dyes in industrial effluents, necessitating continued research and development [27].

While several review papers have previously explored the applications of CBMs in water treatment processes [28–31], there is a critical need to provide a current comprehensive assessment of research developments in this field, given the rapid technological progress in recent years. Notably, most existing reviews have adopted a fragmented approach, typically focusing on either (i) individual types of carbon materials (e.g., graphene or activated carbon alone), (ii) single treatment techniques (adsorption or photocatalysis separately), or (iii) conventional carbon materials without adequate coverage of emerging agro-waste-derived nano-activated composites. Furthermore, previous reviews have insufficiently addressed the synergistic integration of chemical modification strategies with nanotechnology applications, nor have they comprehensively evaluated the transition from laboratory-scale innovations to industrial-scale implementation challenges. Consequently, this study aims to provide a comprehensive analysis of carbon-based materials for the removal of organic dyes from wastewater, distinguishing itself through several unique contributions: (1) a holistic examination of both adsorption and photocatalytic mechanisms within a unified framework; (2) comprehensive coverage of the latest sustainable approaches utilizing agricultural waste-derived nano-activated carbons and nanocomposites; (3) critical analysis of synergistic chemical and nanotechnological modification strategies for performance enhancement; (4) systematic evaluation of scalability challenges and economic feasibility for industrial applications; and (5) identification of emerging trends and future research directions based on the most recent literature (2020-present). The review critically examines recent advances in the field, focusing on innovations that enhance removal efficiency, selectivity, and sustainability, thereby providing researchers and practitioners with an up-to-date, integrated perspective that bridges fundamental research with practical implementation strategies.

## Different carbon-based nanomaterials: structure, properties, synthesis, and their role in water treatment

### Graphene

**Fig. 1:**
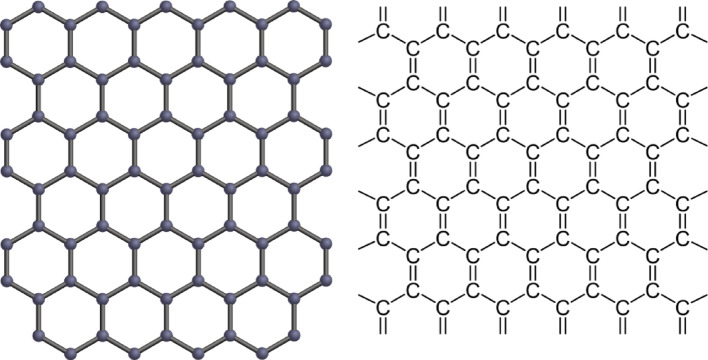
Schematic representation of the graphene structure

Graphene can be defined as a single two-dimensional layer of carbon atoms bound in a hexagonal lattice structure (Fig. 1), and it is stated to be one of the gifted carbon materials [32]. It is also known to be a zero-gap semiconductor, and this is because the conduction and the valence band meet at the Dirac points. It can adsorb and desorb various atoms and molecules such as OH, NH_3_, K, and NO_2_ [33]. Graphene, as a single atomic plane of carbon, can be wrapped up into other graphitic materials such as carbon nanotubes, fullerene, and thin graphene films. Graphene can be divided into a single layer, which is defined as a single two-dimensional hexagonal sheet of carbon atoms; bilayer and few-layer graphene, which also have 2 and 3–10 layers of such two-dimensional sheets, respectively [34]. As a result of graphene’s exceptionally high crystal quality and massless Dirac fermions, monolayer graphene shows an anomalous half-integer quantum Hall effect, remarkable optical properties, ultra-high intrinsic strength, superior thermal conductivity, and extremely high charge carrier mobility. The development of technologies for their industrial manufacture is required due to the widespread use of graphene-based materials in numerous technical domains. Studies show that graphene has the potential for application in the fabrication of highly selective and permeable separation membranes with better performance for water purification, as compared to inorganic filtration membranes or polymer-based membranes [35]. Exfoliation, reduction, electrochemistry, and breakdown (microwave, thermal, and photochemical) are the different techniques used to prepare graphene, which can be obtained by cleaving natural graphite. For pollution control, graphene and its derivatives have been studied [36].

### Graphene oxide

**Fig. 2:**
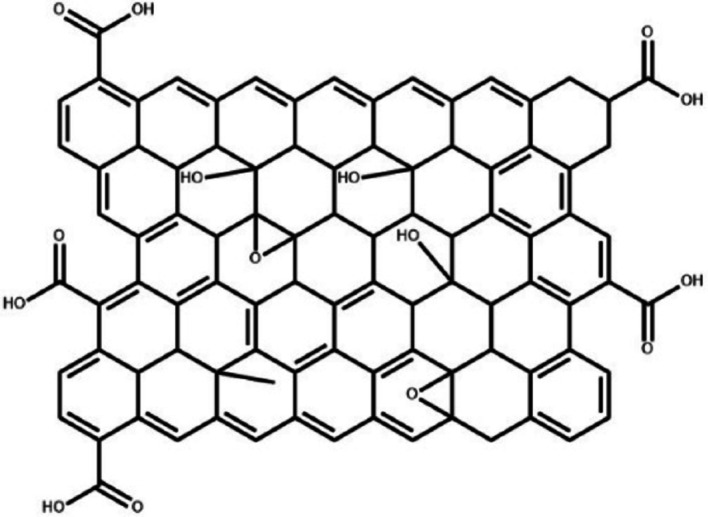
Schematic diagram of a graphene oxide structure

Graphene oxide (GO) represents a unique atomic structure with a single layer containing an arrangement of carbon, hydrogen, and oxygen molecules, which is created when graphite undergoes oxidation [32]. While maintaining significant characteristics of graphene, GO distinguishes itself through its oxygen-containing functional groups (Fig. 2). Graphene oxide demonstrates excellent water affinity and can form stable dispersions in water-based environments due to its polar functional groups [35]. This feature not only simplifies processing through dispersions but also endows GO with superior colloidal stability and an impressive array of mechanical, colloidal, and optical characteristics [37]. In contrast to graphene, graphene oxide (GO) has oxygen-functional groups that significantly augment its chemical reactivity, resulting in a surface abundant with potential binding sites for ionic and molecular interactions [38]. GO-based membranes, being economically viable with impressive performance characteristics, are positioned to revolutionize industrial wastewater treatment. Their key strengths include robust resistance to organic solvents and oxidants, enhanced water attraction properties, reduced membrane clogging, and more precise filtration capabilities [36]. Studies show that optimizing the distance between layers is crucial for maximizing the effectiveness of graphene oxide membranes [37].

#### Challenges in the industrial-scale application of graphene oxide

Despite graphene oxide’s (GO) exceptional properties and versatility across applications from advanced composites to biomedical devices, its industrial-scale deployment faces multifaceted challenges that significantly constrain commercial viability [39].

High production costs represent the principal hindrance to widespread GO adoption [40]. The expense stems from costly raw materials, energy-intensive synthesis processes, and complex purification requirements. These economic blockades limit GO’s practical application across industries, mainly in cost-sensitive sectors like wastewater remediation [41]. The absence of cost-effective mass-production approaches continues to impede the broader implementation of graphene-based materials [42]. Industrial-scale GO production requires significant advances in process optimization and structural uniformity control. Main challenges include the selection and sourcing of suitable graphite precursors and reaction media, which directly influence product quality and consistency. The optimization of oxidation processes remains challenging when scaling from laboratory to industrial production levels [39, 40].

Scalability presents another critical challenge, as current production methods struggle to transition from small-scale laboratory synthesis to continuous industrial manufacturing [43]. Maintaining consistent material properties across large production batches while ensuring reproducibility remains technically demanding and economically challenging.

Addressing these interconnected challenges requires coordinated efforts across multiple disciplines, including the development of more economical synthesis routes, improved process automation, and standardized quality control protocols. Without resolving these fundamental production and scalability issues, GO’s full technological potential will remain unrealized despite its promising material properties. The industrial application of GO thus requires a balanced assessment that acknowledges both its exceptional capabilities and the persistent challenges that must be overcome for successful commercial implementation.

### Reduced graphene oxide

Reduced graphene oxide features a modified graphene structure that is fundamentally different from its precursor, graphene oxide. The key structural characteristic of rGO is the significant reduction in oxygen functional groups, particularly hydroxyl groups, compared to graphene oxide [44]. This structural modification results from the removal of oxygen-containing functional groups that were present in the original graphite oxide, which is a compound of carbon, oxygen, and hydrogen in variable ratios. Unlike pristine graphene, rGO maintains a two-dimensional honeycomb lattice of sp² hybridized carbon atoms while incorporating structural defects, vacancies, and residual oxygen-containing functional groups that remain after the reduction process [45]. The structural characteristics of rGO directly influence its performance as an adsorbent. Its high specific surface area provides numerous active sites for dye molecule interaction [46]. The presence of micropores and mesopores, created during the reduction process, enhances the accessibility of these sites while maintaining the material’s mechanical integrity [47]. Furthermore, the retention of functional groups such as hydroxyl, carboxyl, and epoxy moieties, though reduced compared to the parent graphene oxide, provides chemical anchoring points that facilitate multiple interaction mechanisms with organic dyes [48].

The physicochemical properties of rGO reflect its intermediate position between graphene oxide and pristine graphene. With an oxygen content typically ranging from 5 to 20% by weight, rGO exhibits amphiphilic characteristics, possessing both hydrophobic carbon domains and hydrophilic functional group regions [49]. This dual nature is particularly advantageous for dye adsorption, as it enables interaction with both polar and non-polar dye molecules. The material’s electrical conductivity, tunable band gap, and thermal stability up to 600 °C in inert atmospheres further enhance its applicability in various environmental conditions [50].

#### Synthesis methodologies and process optimization

The synthesis of rGO involves the controlled reduction of graphene oxide through various methodologies, each offering distinct advantages and limitations [47]. Chemical reduction remains the most widely employed approach due to its simplicity and scalability. Common reducing agents include hydrazine hydrate, sodium borohydride, L-ascorbic acid, and increasingly, green alternatives such as plant extracts. The process typically involves dispersing graphene oxide in an aqueous medium, adding the reducing agent, and heating the mixture at temperatures ranging from 60 to 95 °C for several hours [41]. While this method offers excellent control over the reduction process and can be performed at relatively mild conditions, concerns regarding the toxicity of certain reducing agents and incomplete reduction have driven research toward alternative approaches.

Thermal reduction represents another significant synthesis route, involving the high-temperature treatment of graphene oxide powder in inert atmospheres [51]. Operating at temperatures between 200 and 1000 °C, this method achieves high degrees of reduction without chemical residues [52]. However, the high energy requirements and potential for structural damage at extreme temperatures limit its widespread adoption. Electrochemical reduction has emerged as an environmentally friendly alternative, offering precise control over the reduction degree through applied potential manipulation [53]. Despite its advantages in terms of environmental impact and controllability, scalability remains a significant challenge for this approach.

Recent developments in microwave-assisted and photochemical reduction methods have shown promise for rapid, energy-efficient synthesis. Microwave-assisted reduction utilizes rapid heating to achieve reduction in minutes rather than hours [54], while photochemical methods employ light irradiation to drive the reduction process under mild conditions. These emerging techniques represent important steps toward sustainable, large-scale rGO production for commercial applications.

#### Mechanisms of organic dye removal

The exceptional performance of rGO in organic dye removal stems from multiple, often synergistic, interaction mechanisms [55]. π-π stacking interactions represent the primary mechanism for aromatic dye removal, with the delocalized π-electron system of rGO forming strong interactions with the aromatic rings present in most organic dyes. This mechanism is particularly effective for dyes such as methylene blue and rhodamine B, where the aromatic structures can approach within 3.3–3.6 Å of the rGO surface, creating stable adsorption [56].

Electrostatic interactions play a crucial role, particularly for ionic dyes, where the surface charge of rGO interacts with oppositely charged dye molecules. The pH-dependent nature of these interactions allows for optimization of removal efficiency through solution pH adjustment [57]. Hydrogen bonding between residual functional groups on rGO and appropriate sites on dye molecules provides additional binding strength, while hydrophobic interactions between non-polar regions of dyes and the carbon surface contribute to the overall adsorption capacity through van der Waals forces [58].

The relative contribution of these mechanisms varies with dye structure, solution conditions, and rGO characteristics. Understanding these interactions is crucial for optimizing removal efficiency and designing targeted modifications to enhance selectivity for specific dye classes. The multiplicity of interaction mechanisms also explains the broad-spectrum effectiveness of rGO against various dye types, from cationic methylene blue to anionic Congo red [59].

### Graphite

 Graphite, a naturally occurring form of carbon, has gained significant attention as a source of carbon-based materials for various applications. Like diamond, graphite is a crystalline form of carbon. Both graphite and diamond are naturally occurring allotropes of carbon, which are distinct molecular forms of the same element. These allotropes of carbon are created when carbon atoms are linked and ordered to form regular structures. The hardest mineral known to man is diamond, which is made up of four carbon atoms that are connected by strong covalent bonds to one another in a regular isometric structure.

**Fig. 3:**
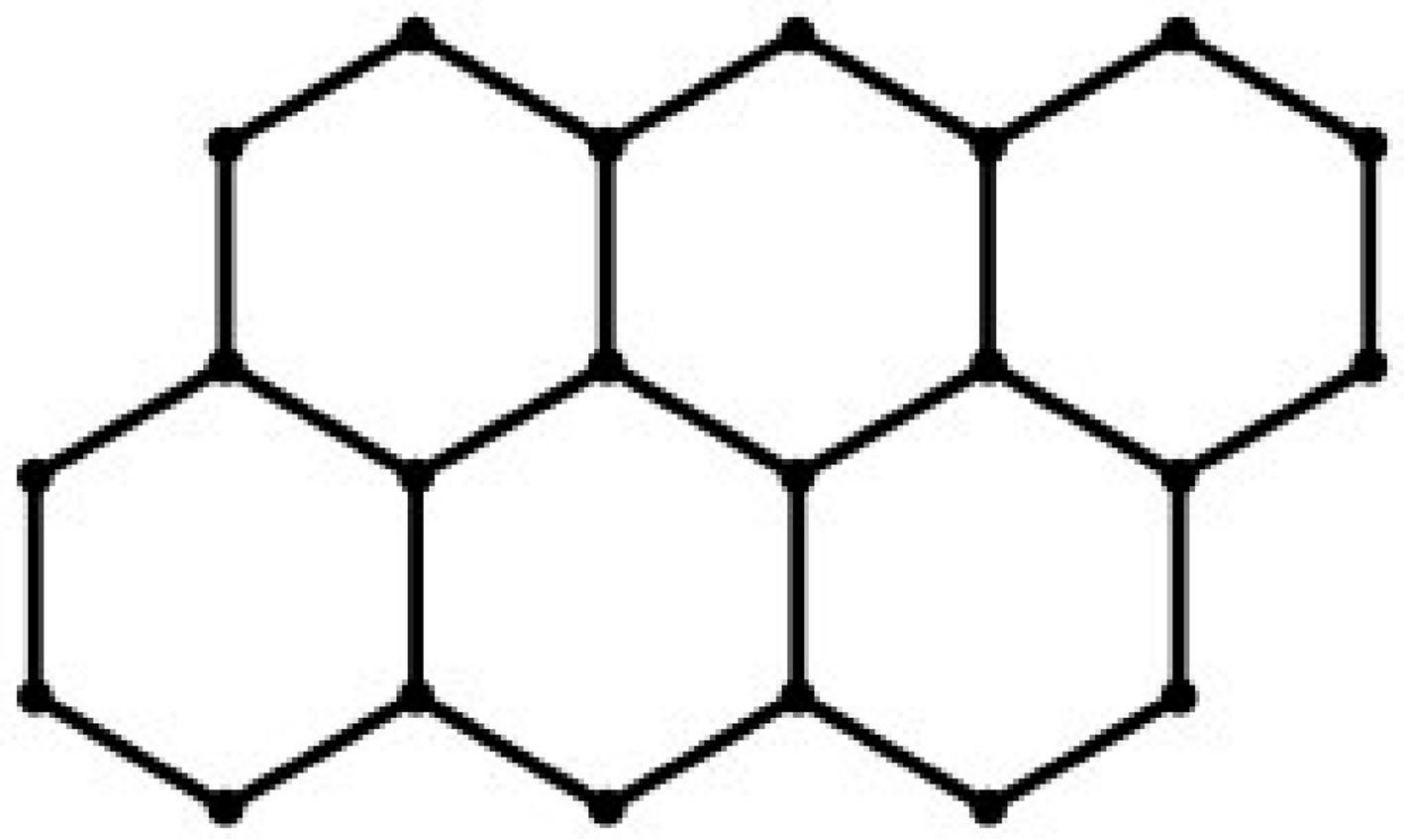
Schematic representation of a graphite structure

In graphite, carbon atoms are only attached to three other carbon atoms, resulting in strong, two-dimensional layers (Fig. 3) that are incredibly stable, but each layer is only weakly connected to adjacent layers by van der Waals forces. The resulting hexagonal layered structure creates one of the softer minerals [60, 61]. Moreover, the presence of delocalized π-electrons within the layers facilitates the transfer of electrons, making graphite an effective material for electron transport in photocatalytic processes [62]. Graphite shares several characteristics with carbon-based materials, including a low specific gravity and chemical inertness that can be maintained up to 900 K. For oxygen, however, it has been proven that graphite reacts with oxygen above this temperature without degrading. Moving on to the properties, graphite also has a high coefficient of friction, a high neutron attenuation factor, a low neutron absorption cross-section, and a sublimation temperature above 3900 K. This makes it an ideal moderator for nuclear reactors. Sometimes, at 3500 K, carbon compounds can still maintain their mechanical capabilities, but their resistivity will vary greatly depending on how they are heated or how they interact with other substances [63]. Although the space between the carbon layers in hydrocarbon materials is higher than in natural monocrystalline graphite, artificial graphite almost has the same crystal structure. Though anisotropic, graphite has a very high thermal conductivity in a direction parallel to the plane of the layers. Graphite’s predicted crystal density is 2.266 g/cm^3^; however, measurements of its specific gravity range from 2.20 to 2.30 depending on purity. High values are primarily due to impurities, while low values are linked to trapped porosity [64]. Graphite is used to manufacture lubricants and pencils. Since it has a high conductivity and a low neutron absorption cross-section, it is employed in nuclear reactors as a moderator and as a refractory material. It is also helpful in electronic devices, including electrodes, batteries, and solar panels [65].

#### Environmental impact of graphite extraction and use in organic contaminant removal: sustainability perspectives

The environmental consequences of graphite extraction and use for eliminating organic contaminants, predominantly dyes, necessitate careful deliberation within the wider context of sustainable wastewater remediation technologies. A number of environmental issues are raised by graphite mining and processing activities, such as disturbance of natural habitats, energy-intensive extraction procedures, and possible groundwater contamination. The carbon footprint of graphite manufacturing stands in contrast to the industry’s increasing focus on environmentally friendly wastewater treatment techniques for wastewater containing dyes [66]. This contradiction emphasizes the necessity of life-cycle analyses when assessing graphite-based adsorbents for the removal of organic pollutants. Despite extraction concerns, graphite-based materials offer significant environmental benefits when applied to textile wastewater treatment. Textile dyeing wastewater contains complex pollutants, including organic dyes, inorganic salts, heavy metals, and surface-active dispersants [67], making graphite’s versatile adsorption properties particularly valuable. The physical adsorption mechanisms of graphite-based materials provide an environmentally preferable alternative to energy-intensive chemical treatments like electro-Fenton, photocatalysis, and ozonation [68]. Recent advancements in 2023 have focused on employing nanoparticles to remediate solutions contaminated with deleterious organic dyes [69], with a growing focus on environmentally friendly biological approaches [70]. The incorporation of graphite-based adsorbents into biological treatment procedures is a promising strategy that blends the sustainability of biological systems with the efficiency of physical adsorption [71].

#### Mechanism of dye removal

 Graphite, while possessing a layered sp² carbon structure that enables weak π–π interactions with aromatic dye molecules, exhibits limited direct applicability in dye removal due to its low specific surface area and hydrophobic nature. However, it serves as a crucial precursor for high-performance carbon materials such as graphene oxide (GO) and expanded graphite, which are far more effective adsorbents [72]. When modified, such as through chemical or thermal expansion, graphite gains increased surface area and edge defects, enhancing its adsorption capacity. Expanded graphite can effectively remove dyes via physisorption, pore trapping, and weak electrostatic interactions, with studies reporting removal efficiencies exceeding 85% for certain pollutants [73]. In composite systems, graphite particles provide structural stability and electrical conductivity, supporting photocatalytic or adsorptive components [74]. From a green synthesis perspective, graphite derived from natural or recycled sources aligns with sustainable material sourcing, and its use as a precursor in low-energy conversion processes contributes to environmentally friendly water treatment solutions.

### Graphitic carbon nitride (g-C_3_N_4_)

The development of efficient and sustainable photocatalytic materials for the removal of organic pollutants from wastewater has become increasingly important. Graphitic carbon nitride (g-C_3_N_4_) has emerged as a promising carbon-based photocatalyst due to its unique properties and the abundance of carbon. This section aims to provide a detailed overview of the synthesis methods employed to prepare g-C_3_N_4_ for photocatalysis, while also highlighting its structural properties and potential applications in environmental remediation.

**Fig. 4:**
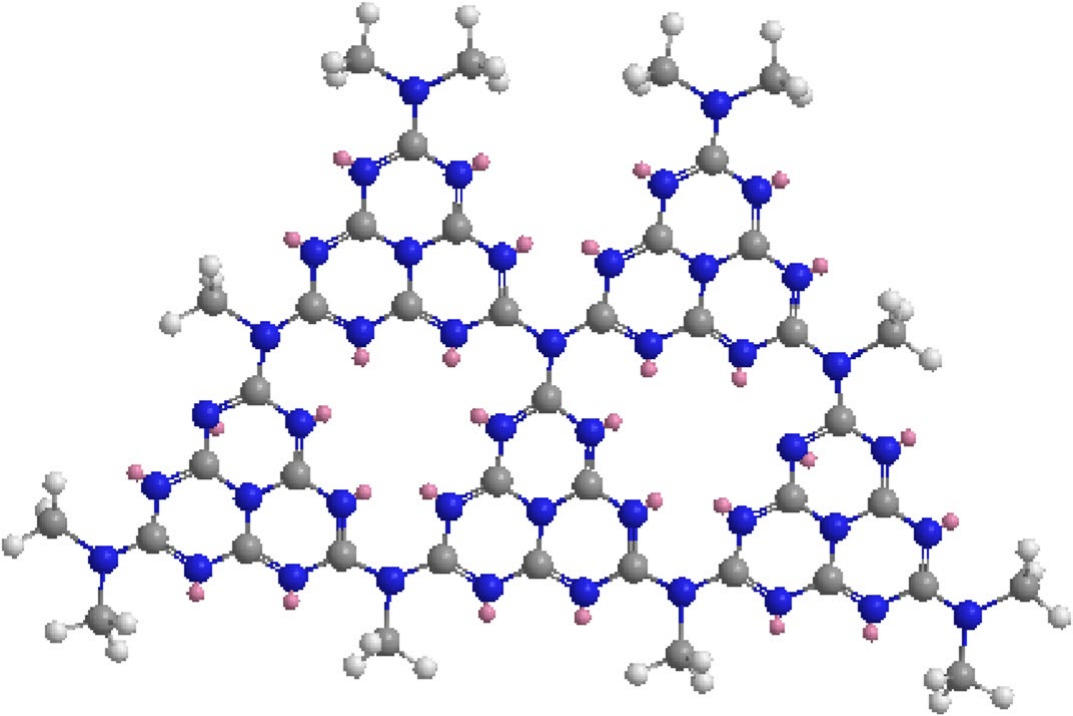
Structure of g-C_3_N_4_

Graphitic carbon nitride (g-C_3_N_4_) possesses several key properties that make it attractive for photocatalytic applications. It has an optimal bandgap energy of approximately 2.7 eV, enabling it to absorb visible light and utilize a significant portion of the solar spectrum for photocatalysis [75]. Additionally, g-C_3_N_4_ exhibits high stability, both thermally and chemically, making it a durable photocatalyst even under harsh reaction conditions. The carbon-based nature of g-C_3_N_4_ provides advantages such as low cost, scalability, and eco-friendliness, making it an attractive alternative to metal-based photocatalysts [76]. Furthermore, g-C_3_N_4_ has a unique electronic structure (Fig. 4) that facilitates efficient charge separation and migration, leading to the generation of reactive oxygen species (ROS) for photocatalytic reactions. Its notable photocatalytic activity enables various reactions, including organic dye degradation, water splitting, and pollutant removal [77].

Conventional synthesis methods for g-C_3_N_4_ involve direct thermal polymerization and template-assisted methods. Direct thermal polymerization is a common approach that utilizes cyanamide or melamine precursors. The precursor undergoes condensation reactions, forming a polymeric structure with a g-C_3_N_4_-like composition [78]. Researchers such as Wang et al. [78] have employed urea, melamine, and cyanuric acid as the precursors and conducted thermal polymerization to obtain g-C_3_N_4_ with a layered structure. In another study, Antil et al. [79] synthesized g-C_3_N_4_ nanosheets using thermal polymerization. In their approach, equal quantities of melamine and ammonium carbonate were put together in a covered crucible and heated at 550 °C for 5 h in a muffle furnace. HCl was added to the product in a beaker after it had been crushed into powder and stirred for 12 h, which was followed by centrifugation, washing, and drying.

Template-assisted methods provide control over the morphology and porosity of g-C_3_N_4_. For instance, Goettmann et al. [80] utilized a hard-template method, employing a silica template to guide the formation of g-C_3_N_4_ with a mesoporous structure. The template was subsequently removed by the use of ammonium bifluoride or hydrogen fluoride, resulting in well-defined porous g-C_3_N_4_. Due to the use of hazardous compounds to remove the hard template, an alternative technique (soft-template method) has been developed and explored [81]. Wang et al. [82] used imidazolium and pyridine-based ionic liquids to synthesise a nano-porous graphitic carbon nitride photocatalyst with exceptional characteristics.

Other synthesis strategies include the sol-gel method and the chemical co-precipitation method. The sol-gel method allows for the controlled synthesis of g-C_3_N_4_ using liquid precursors and controlled hydrolysis and condensation reactions. Alhaddad et al. [83] employed the sol-gel technique to synthesize g-C_3_N_4_ and its composites with improved crystallinity and enhanced photocatalytic activity. Chemical co-precipitation has also been used by Praus et al. [75] to fabricate g-C_3_N_4_ for the decomposition of N_2_O.

In summary, the synthesis of g-C_3_N_4_ as a carbon-based material for photocatalysis is a rapidly evolving field. g-C_3_N_4_ possesses unique properties, including optimal bandgap energy, high stability, abundance of carbon, unique electronic structure, and notable photocatalytic activity. Various synthesis methods, including direct thermal polymerization, template-assisted methods, sol-gel, and chemical co-precipitation, provide avenues for tailoring the structural and morphological properties of g-C_3_N_4_. These synthesis strategies pave the way for harnessing the potential of g-C_3_N_4_ in photocatalytic applications for organic dye degradation, water splitting, and environmental remediation. Table 1 gives a summary of some research on the photocatalytic degradation of pollutants using g-C_3_N_4_.

**Table 1:** Photocatalytic degradation of pollutants using g-C_3_N_4_-based photocatalysts

g-C_3_*N*_4_-based photocatalyst	Synthesis method	Pollutant removed	Removal efficiency/time	References
WO_3_/ g-C_3_N_4_	Physico-chemical technique	Rhodamine B	99.5%	[84]
g-C_3_N_4_/MoS_2_/Bi_2_O_3_	Hydrothermal-calcination	Methylene blue	98.5% /90 min	[85]
MoS_2_/S-doped porous g-C_3_N_4_	Ultrasound assembly and calcination	Rhodamine B	91.1%/15 min	[86]
WO_3_/ g-C_3_N_4_	Wet chemical process	Remazol brilliant red X-3BS Methylene blue	92.4%/210 min 97.8%/160 min	[87]
g-C_3_N_4_/-3ZnO-c-Zn_2_Ti_3_O_8_	Sol–gel and calcinations	Methylene blue	∼99% /45 min	[88]
Carbon quantum dots/ZnO/AgI phosphorus-doped g-C_3_N_4_	Hydrothermal	2,4-dinitrophenol	98.0%/140 min	[89]
Ag_3_PO_4_/modified P and S co-doped g-C_3_N_4_	Deposition-precipitation	2,4-dimethyl phenol	97.0%/120 min	[90]
Bi_24_O_31_C_l1_0/MoS_2_/g-C_3_N_4_	Impregnation–calcination	Tetracycline	97.5% /50 min	[91]
AgI/LaFeO_3_/g-C_3_N_4_	Ultrasound-assisted hydrothermal	Norfloxacin	95%/120 min	[92]

#### Graphitic carbon nitride (g-C_3_N_4_) as an adsorbent

The layered 2D architecture of g-C₃N₄ creates interlayer spaces (typically 0.326 nm) that facilitate molecular intercalation, while the π-conjugated framework enables strong π-π interactions with aromatic pollutants [93]. The abundance of nitrogen-containing functional groups (-NH_2_, -NH-, pyridinic N, pyrrolic N) provides multiple binding sites through various mechanisms, including electrostatic interactions, hydrogen bonding, and coordination bonding with metal ions [94]. Recent studies demonstrate g-C_3_N_4_’s versatility in removing organic dyes (methylene blue, congo red), achieving 100–500 mg/g [95, 96], heavy metals and emerging contaminants, including pharmaceuticals and endocrine disruptors [97] This broad-spectrum removal capability positions g-C_3_N_4_ as a universal adsorbent platform.

Compared to activated carbon, g-C_3_N_4_ offers superior selectivity due to its nitrogen-rich surface chemistry, easier regeneration without structural degradation, and the unique advantage of simultaneous adsorption-photocatalytic degradation under light irradiation [98]. The material demonstrates excellent reusability (> 5 cycles with > 80% efficiency retention) and chemical stability across pH ranges of 2–12 [99, 100]. g-C_3_N_4_’s dual functionality allows for synergistic adsorption-photocatalysis processes where pollutants are first concentrated on the surface through adsorption, then degraded in situ under light irradiation [101, 102].

### Carbon nanotubes (CNTs)

Carbon nanotubes (CNTs) have emerged as a promising source of carbon-based materials for various applications, including photocatalytic water treatment. With their unique structural, mechanical, and electrical properties, CNTs offer significant potential in revolutionizing water treatment technologies by harnessing the power of light-driven processes [103]. By leveraging their exceptional photocatalytic properties, high surface area, and efficient charge transport, CNTs provide new avenues for the degradation of organic pollutants and the purification of water resources through photocatalysis [104]. Carbon nanotubes are cylindrical nanostructures composed of carbon atoms arranged in a hexagonal lattice, forming a tubular shape with diameters in the nanometer range. They can be single-walled (SWCNTs), consisting of a single graphene layer, or multi-walled (MWCNTs), comprising multiple concentric graphene layers. This unique structure provides CNTs with a large surface area and excellent light absorption properties, making them ideal candidates for photocatalytic applications in water treatment [105].

In recent years, there has been a growing interest in the synthesis of CNTs with different structures and morphologies to enhance their photocatalytic activity for water treatment applications. The synthesis of SMCNTs and MWCNTs has gained significant attention due to their unique properties and potential applications in photocatalysis [106]. This section aims to provide an overview of the synthesis methods of SMCNTs and MWCNTs as carbon-based materials for photocatalytic water treatment.

#### Single-walled carbon nanotubes (SWCNTs)

**Fig. 5:**
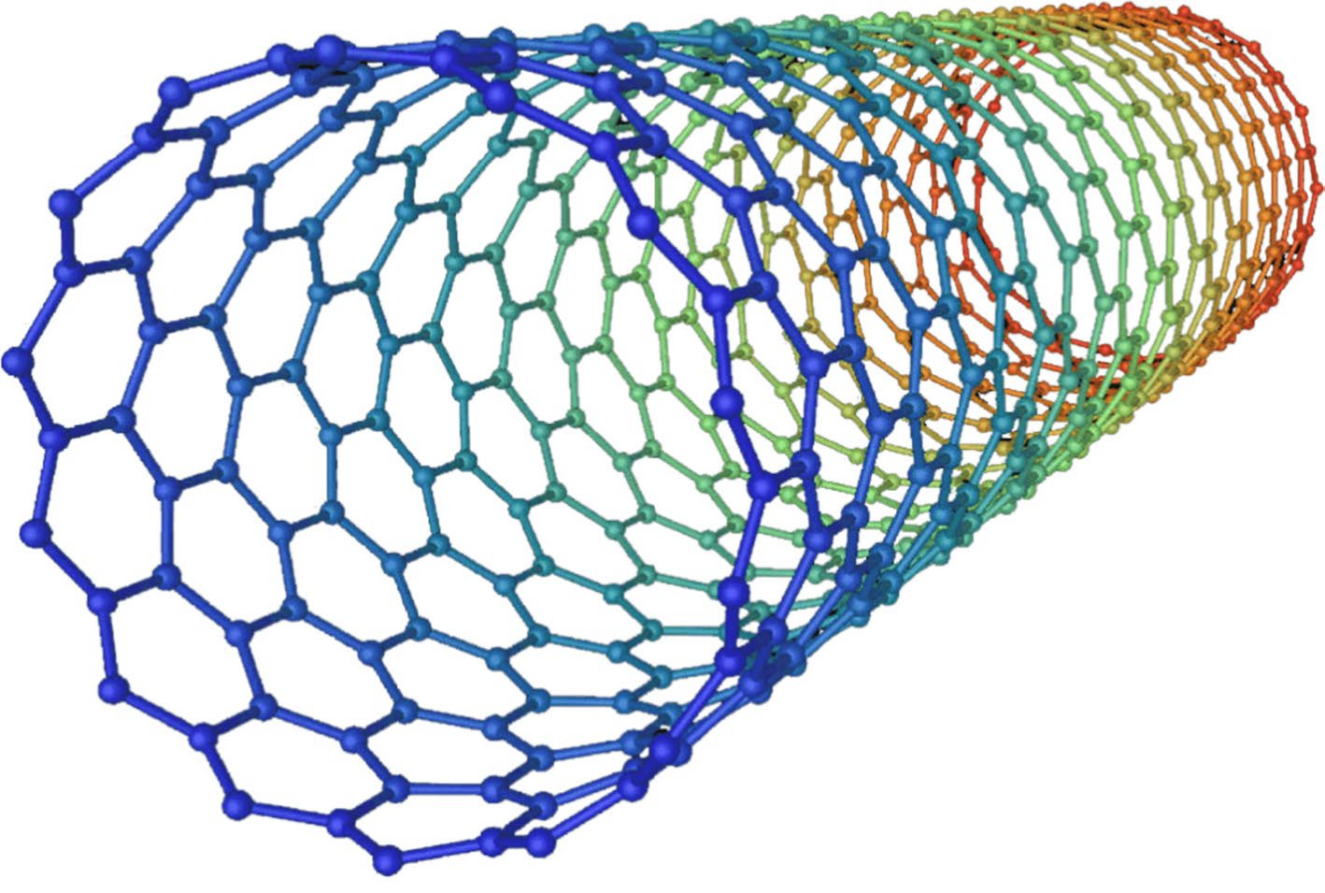
Schematic representation of a single-walled carbon nanotube (SWCNT)

SWCNTs are one-dimensional nanostructures composed of rolled graphene sheets, resembling a seamless cylinder with a diameter on the nanometer scale and lengths that can extend up to several centimetres (Fig. 5) [107, 108]. SWCNTs exhibit remarkable mechanical, electrical, and thermal characteristics, which make them unique and valuable in various applications [107, 109]. These unique properties stem from their nanoscale dimensions, leading to fascinating phenomena such as quantum confinement and ballistic electron transport, which make SWCNTs highly desirable for photocatalysis applications [106]. The nanoscale dimensions of SWCNTs grant them exceptional mechanical strength, surpassing that of steel while maintaining low density, making them unparalleled reinforcements for composite materials and nanoscale engineering. Their high aspect ratio contributes to a vast surface area, facilitating chemical interactions and reactivity that open up possibilities for catalytic and sensing applications [110].

#### Multiwalled carbon nanotubes (MWCNTs)

**Fig. 6:**
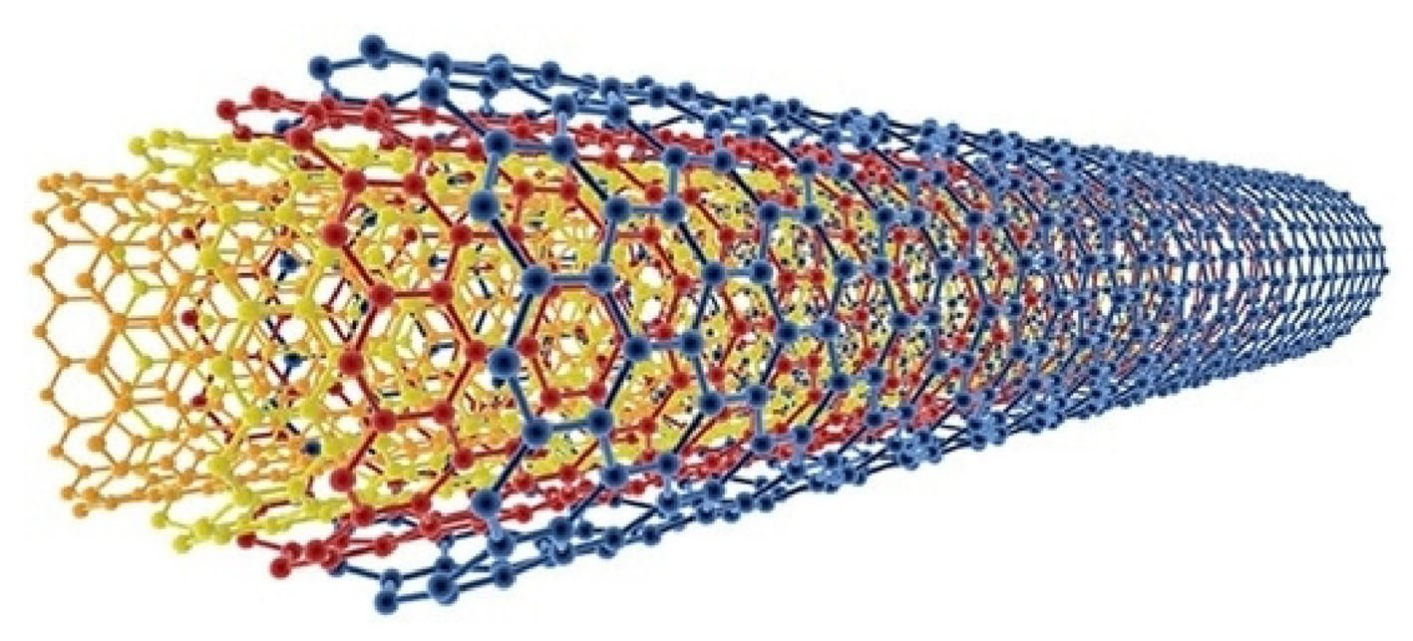
Schematic representation of multiwalled carbon nanotube (MWCNT)

Multiwalled carbon nanotubes (MWCNTs) consist of multiple concentric graphene layers arranged in a cylindrical structure (Fig. 6). MWCNTs exhibit excellent mechanical strength, thermal conductivity, and stability, making them suitable for various applications, including photocatalytic water treatment. The presence of multiple walls in MWCNTs provides enhanced adsorption capacity, surface reactivity, and stability compared to SWCNTs [110]. MWCNTs demonstrate significant potential for photocatalytic water treatment due to their unique structural and surface properties. The multiple concentric layers provide a higher surface area for pollutant adsorption, while the interconnected channels between the layers allow efficient mass transfer and facilitate photocatalytic reactions [111]. The stability and recyclability of MWCNTs make them suitable for repeated use in water treatment processes [112].

#### Synthesis of SWCNTs and MWCNTs

The synthesis of SWCNTs and MWCNTs has been extensively investigated to control their structural and morphological properties, which directly influence their photocatalytic and adsorption performances. Various synthesis methods have been employed, including chemical vapour deposition (CVD), arc discharge, and laser ablation [113]. Each method offers distinct advantages and limitations, and the choice of synthesis technique depends on the desired characteristics of the resulting CNTs [114].

##### Chemical vapour deposition (CVD)

Chemical vapour deposition (CVD) is one of the most widely used methods for synthesizing SWCNTs and MWCNTs. It involves the decomposition of hydrocarbon precursors at elevated temperatures in the presence of a catalyst. The catalyst plays a crucial role in determining the diameter, chirality, and structure of the resulting CNTs [108, 114]. Transition metals such as iron, cobalt, and nickel are commonly used as catalysts. The optimization of synthesis parameters, including temperature, precursor gas composition, and catalyst preparation, enables the control of CNT growth and the achievement of desired properties [115]. One of the key advantages of CVD is its ability to produce CNTs with controlled structures. The catalyst particles act as nucleation sites for CNT growth, size control, and distribution, which directly impact the diameter and chirality of the nanotubes.

Over the years, researchers have developed various catalyst engineering strategies to tailor the CNT properties, including single-walled and multi-walled configurations, and to selectively grow semiconducting or metallic CNTs [110]. Floating catalyst CVD is a widely used method for CNT synthesis, offering excellent scalability and production rates. However, challenges remain in achieving a high yield of single-walled CNTs with uniform chirality. Plasma-enhanced CVD has shown promise in enhancing selectivity and growth kinetics but requires careful optimization of plasma conditions and precursor gas compositions. Moreover, the role of substrates in CNT growth cannot be overlooked [116]. The choice of substrate material and its surface properties influence catalyst adhesion and CNT alignment, leading to different morphologies and alignments of the nanotubes. Understanding substrate interactions is critical for integrating CNTs into device fabrication and composite materials [117].

##### Arc discharge

**Fig. 7:**
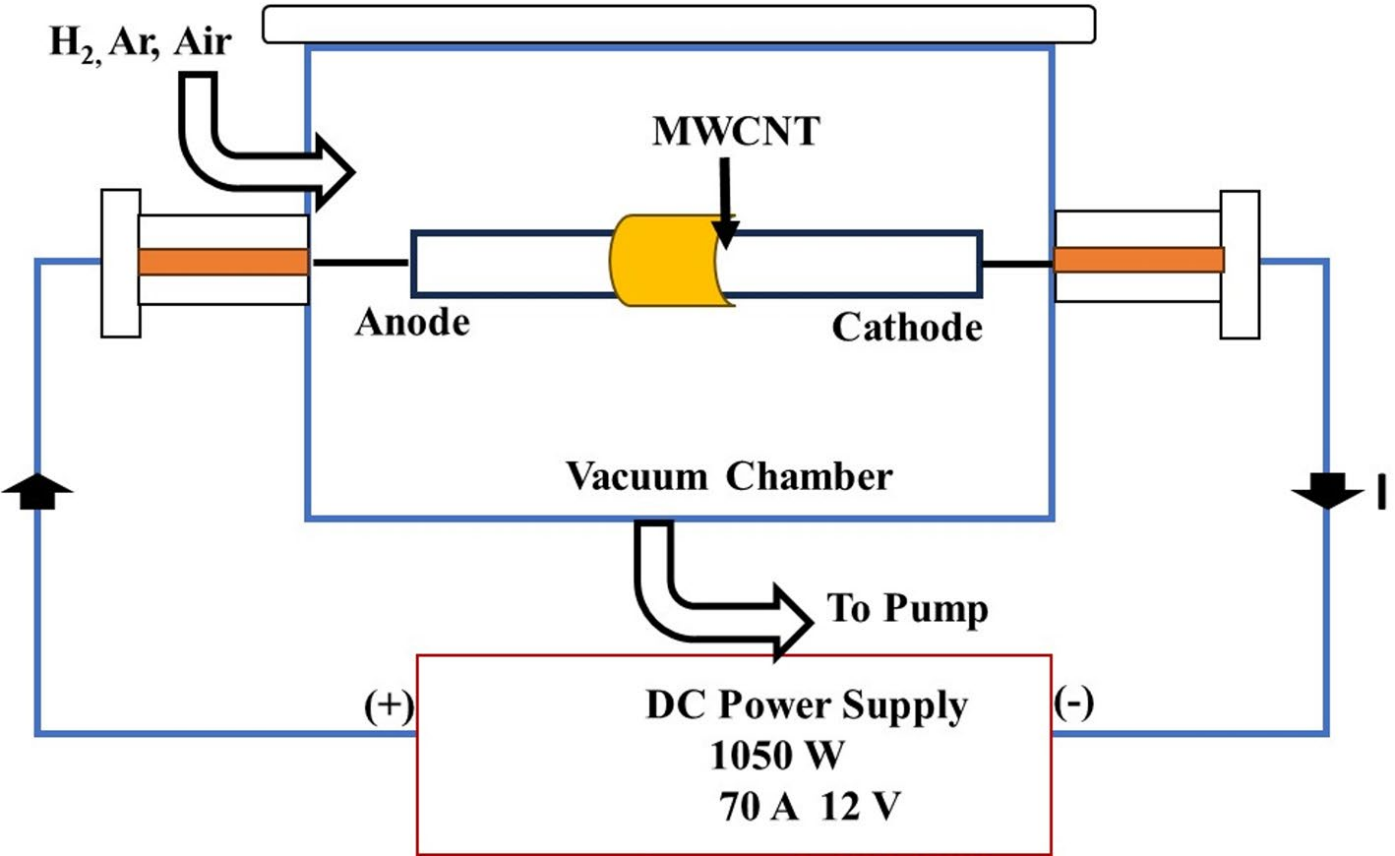
Diagram of an arc discharge method for synthesizing SWCNTs and MWCNTs

The arc discharge is another popular method for synthesizing SWCNTs and MWCNTs. It involves passing a high current through graphite electrodes in an inert atmosphere (Fig. 7). The arc discharge is one of the processes that produce CNTs at higher temperatures (over 1700 °C), which typically results in the formation of CNTs with fewer structural flaws than other processes [118]. The intense heat generated causes vaporization and subsequent condensation of carbon atoms, resulting in the formation of CNTs. This method allows for the production of CNTs with high purity and yield [119]. Without or with the assistance of various catalyst precursors, CNTs could be deposited using an arc discharge. Typically, no catalyst is needed to create MWNTs. On the other hand, when the catalyst made of a transition metal is utilized, SWNTs are created. A composite anode, often in a hydrogen or argon environment, is used in the process of growing SWNTs in an arc discharge [110]. One of the key advantages of the arc discharge method is its ability to produce multi-walled carbon nanotubes (MWCNTs) and, under specific conditions, single-walled carbon nanotubes (SWCNTs). The structure and properties of the synthesized CNTs depend on several factors, including the type of graphite used, the electrode distance, the arc current, and the growth time [120]. Consequently, researchers have explored various parameters to tailor CNT properties for specific applications. The arc discharge method has undergone substantial refinements over the years to enhance the yield, purity, and structural uniformity of the produced CNTs. The introduction of catalysts, such as transition metal nanoparticles, on the graphite electrodes has led to improved CNT growth and control over their diameters. Moreover, the use of magnetic fields during arc discharge has shown promising results in aligning the growing CNTs, which is critical for applications in nanoelectronics and composite materials [121].

##### Laser ablation

**Fig. 8:**
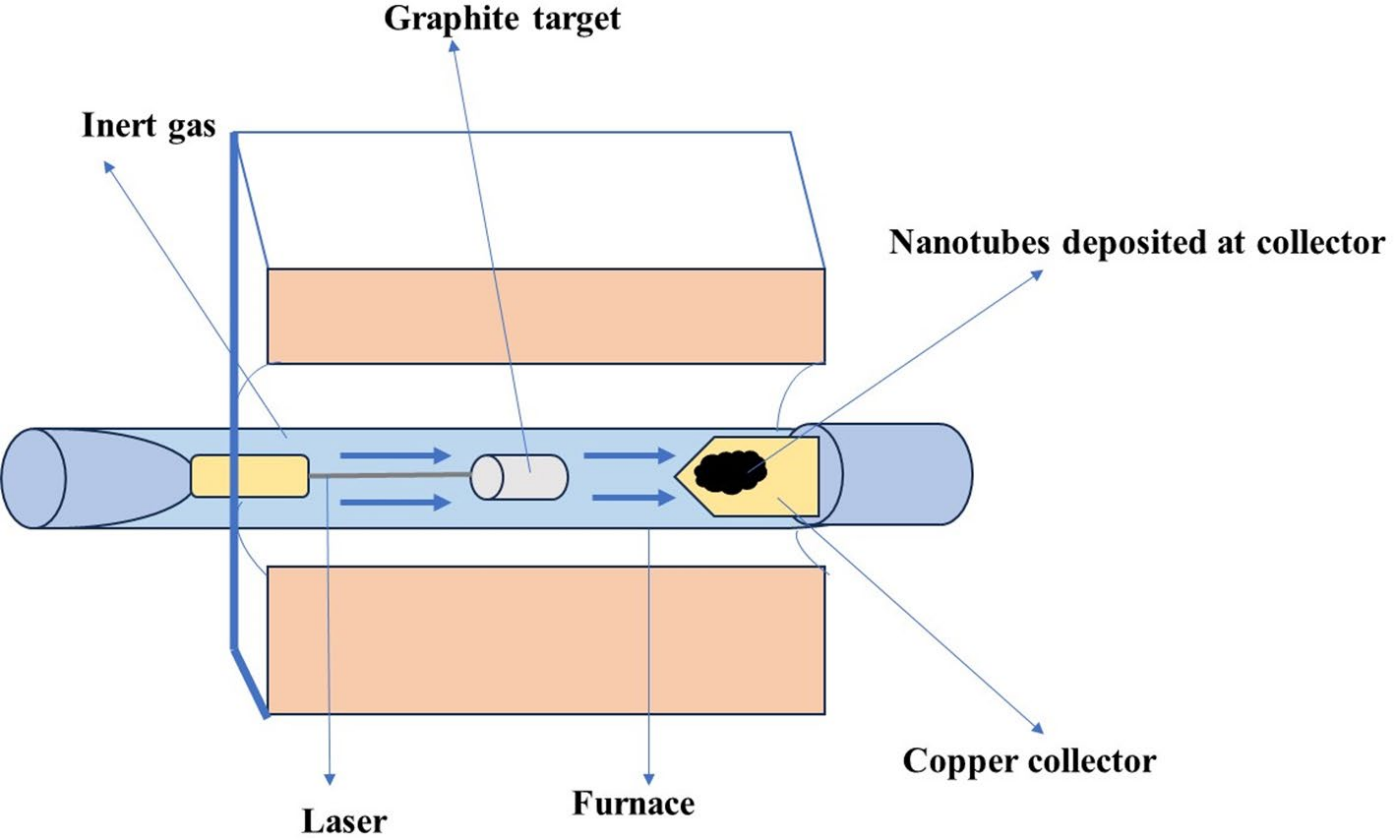
Diagram of a laser ablation technique for the synthesis of SWCNTs and MWCNTs

It is a technique used for the synthesis of SWCNTs and MWCNTs with precise control over their size and structure. In this method, a high-energy laser pulse is used to vaporize a carbon target in the presence of a metal catalyst (Fig. 8). The vaporized carbon atoms condense and form CNTs on a substrate. Although laser ablation enables the production of high-quality CNTs, its scalability and cost-effectiveness remain challenging [122]. One of the remarkable features of laser ablation is the direct growth of CNTs without the need for catalysts. This characteristic eliminates catalyst-related impurities and allows for the production of defect-free nanotubes with excellent electronic and mechanical properties [120]. Furthermore, laser ablation can be performed in various gas environments, enabling the doping of CNTs with different elements and opening up possibilities for tailoring their electronic properties for specific applications. The laser ablation method offers significant advantages in terms of scalability and production rate. It can be easily integrated into continuous processes, making it attractive for industrial-scale synthesis of CNTs [123]. However, challenges remain in controlling the size distribution of the produced CNTs and achieving a higher yield of SWCNTs, which are typically of more significant interest for many applications. To address these challenges, researchers have explored various strategies to optimize the laser ablation process. Fine-tuning the laser parameters, such as pulse duration, energy and wavelength, can significantly influence the characteristics of the synthesized CNTs. Moreover, the use of metal catalysts as seeding layers on the target surface has shown promise in promoting SWCNT growth and controlling their chirality [124].

#### Green and sustainable synthesis pathways for carbon nanotubes

Recent advancements in the synthesis of CNTs have pivoted towards the use of renewable carbon feedstocks, energy-efficient techniques, and non-toxic catalysts to align production with sustainability goals. Bio-based carbon nanotubes synthesized from renewable biomass sources-such as lignin, cellulose, algae, and agricultural or food waste-have gained attention as eco-friendly alternatives to fossil-derived precursors. These biomass-derived feedstocks not only reduce reliance on petroleum-based hydrocarbons but also contribute to lowering the overall carbon footprint by promoting a circular economy through resource reutilization [125].

Energy-efficient synthesis methods have been developed, particularly variations of chemical vapor deposition (CVD) operating at lower temperatures (400–800 °C rather than the conventional 1000+ °C), plasma-assisted synthesis, and hydrothermal carbonization at moderate temperatures (~ 200–300 °C). These approaches significantly reduce energy consumption and greenhouse gas emissions compared to traditional high-temperature methods while maintaining or even improving CNT yield and quality. Additionally, these greener methods often eliminate or reduce the use of hazardous chemicals and solvents, thereby minimizing toxic byproducts in the process [126].

Catalyst selection is pivotal for sustainable synthesis. Non-toxic and earth-abundant catalysts such as manganese oxides, iron, cobalt, and ferrocene derivatives have demonstrated effective CNT growth without introducing significant environmental or health hazards. Catalyst optimization also helps lower reaction temperatures, further enhancing the eco-friendliness of the process. Moreover, catalytic processes using renewable feedstocks have successfully produced both SWCNTs and MWCNTs with desirable structural properties, catering to various application needs [124].

Collectively, these advancements illustrate a promising trajectory for the green and sustainable synthesis of CNTs, balancing the requirements for high-performance nanomaterials with environmental responsibility. Incorporating renewable carbon feedstocks, leveraging energy-efficient synthesis technologies, and employing non-toxic catalysts form the cornerstone of this evolving field.

The transition to green and sustainable CNT synthesis represents more than a technical challenge, it embodies a fundamental commitment to ensuring that advanced nanomaterials contribute to environmental solutions rather than creating new environmental problems. This shift is critical because traditional CNT production methods often involve high energy consumption, toxic byproducts, and significant greenhouse gas emissions, which pose challenges to environmental health and safety. By adopting greener synthesis approaches using renewable biomass feedstocks and energy-efficient processes, the carbon footprint of CNT production can be substantially reduced, aligning the manufacturing phase with broader ecological goals [127].

Moreover, CNTs have demonstrated extraordinary potential in environmental remediation, particularly in removing organic dyes and other contaminants from wastewater. When their production is coupled with sustainable synthesis routes, a holistic environmental benefit is achieved: not only does the application improve water purification and resource conservation, but the material itself is produced in a way that minimizes ecological impact. This synergy creates a circular system where sustainable material production complements environmental protection through water treatment, fostering resource efficiency and reduced pollution [21].

The properties of SWCNTs and MWCNTs can be further enhanced through surface functionalization and modification, which improves their photocatalytic activity for water treatment. Various methods, such as chemical treatment, covalent attachment of functional groups, and doping with heteroatoms, have been employed to modify the surface properties of CNTs [128]. These modifications can improve the adsorption capacity, extend the absorption range, and enhance the photocatalytic efficiency of CNT-based photocatalysts.

In a nutshell, SWCNTs and MWCNTs have enormous potential as carbon-based materials for photocatalytic water treatment. Their exceptional photocatalytic activity is a result of their special characteristics, including a wide surface area, high aspect ratio, and effective charge transfer. In order to customize the structural and surface features of CNTs and affect their photocatalytic performance, the synthesis processes and surface alterations are necessary. The development of effective and environmentally friendly water treatment technologies will be aided by further research and development in this area. Table 2 gives a summary of some CNTs and their related composites used in water photocatalysis of organic dyes.

#### Dye removal mechanism of CNTs

Carbon nanotubes (CNTs) exhibit effective removal of organic dyes through a combination of mechanisms dominated by π-π stacking interactions between the graphitic layers of the nanotubes and the aromatic rings of dye molecules like methylene blue and rhodamine B, and Congo red [128]. The multi-layered cylindrical structure provides a large specific surface area and accessible adsorption sites, while inter-wall spaces and defects act as additional binding pockets for molecular entrapment [129]. Electrostatic interactions significantly contribute to adsorption, particularly when MWCNTs are oxidized to introduce carboxyl or hydroxyl groups that interact with charged dye species [128]. Hydrophobic interactions and pore filling further enhance dye uptake, especially for non-polar aromatic segments. In the framework of green synthesis, CNTs produced from sustainable feedstocks such as plant oils or bioethanol via plasma-enhanced or low-temperature CVD may retain catalytically active metal nanoparticles (e.g., Fe, Ni) that assist both adsorption and photocatalytic degradation under solar irradiation [130]. Their high mechanical strength and thermal stability make CNTs suitable for reusable adsorbent systems, with adsorption kinetics typically following pseudo-second-order models and isotherm behavior fitting the Langmuir model, indicating monolayer adsorption. Integration into composites also improves dispersion and recovery in water treatment applications [131].

**Table 2:** Some CNTs and their related composites used in water photocatalysis of organic dyes

Material type/composite	Method of preparation	Pollutant removed	Degradation/removal efficiency	Time (min)	References
MWCNTs	CVD	Rhodamine B	90%	40	[132]
MWCNTs	–	Methylene Blue and Congo Red	MB − 400 mg/g CR − 500 mg/g	500	[133]
Mn/Fe-AFMCNT	CVD	Acid Blue 92 Acid Red 14 Direct Red 31	AB92–333 mg/g AR14–370 mg/g DR31–323 mg/g	60	[134]
TiO_2_/Graphene-MWCNT	CVD	Methylene Blue	75%	300	[135]
MWCNT-PEN	Simple electrospinning method	Pyrene	96%	30	[136]
MWCNT/TiO_2_	CVD	Methyl orange	90%	90	[137]
MWCNTs-Pt	CVD	Monoazo dye	51.2%	120	[138]

### Activated charcoal

Activated charcoal, also known as activated carbon, is a type of graphite with a rough and imperfect structure. It has a wide range of pores of varying sizes, which significantly increase its surface area and make it useful for various applications such as purifying air and water. The small pores enhance the surface area available for chemical reactions like adsorption and photocatalysis. Before activation, charcoal has a specific surface area of 2.0 to 5.0 m^2^ g^− 1^, which increases to 1000 m^2^ g^− 1^ after activation [134].

The structure of activated charcoal affects its ability to adsorb substances. It is similar to pure graphite, consisting of layers of fused hexagons held together by weak van der Waals forces. However, activated charcoal has a slightly larger interlayer spacing compared to graphite [139]. It can be categorized into graphitizing and non-graphitizing types based on its ability to form graphite. Graphitizing carbon has parallel graphene layers and a sensitive structure, while non-graphitizing carbons have stronger cross-linking and a well-developed microporous structure.

**Fig. 9:**
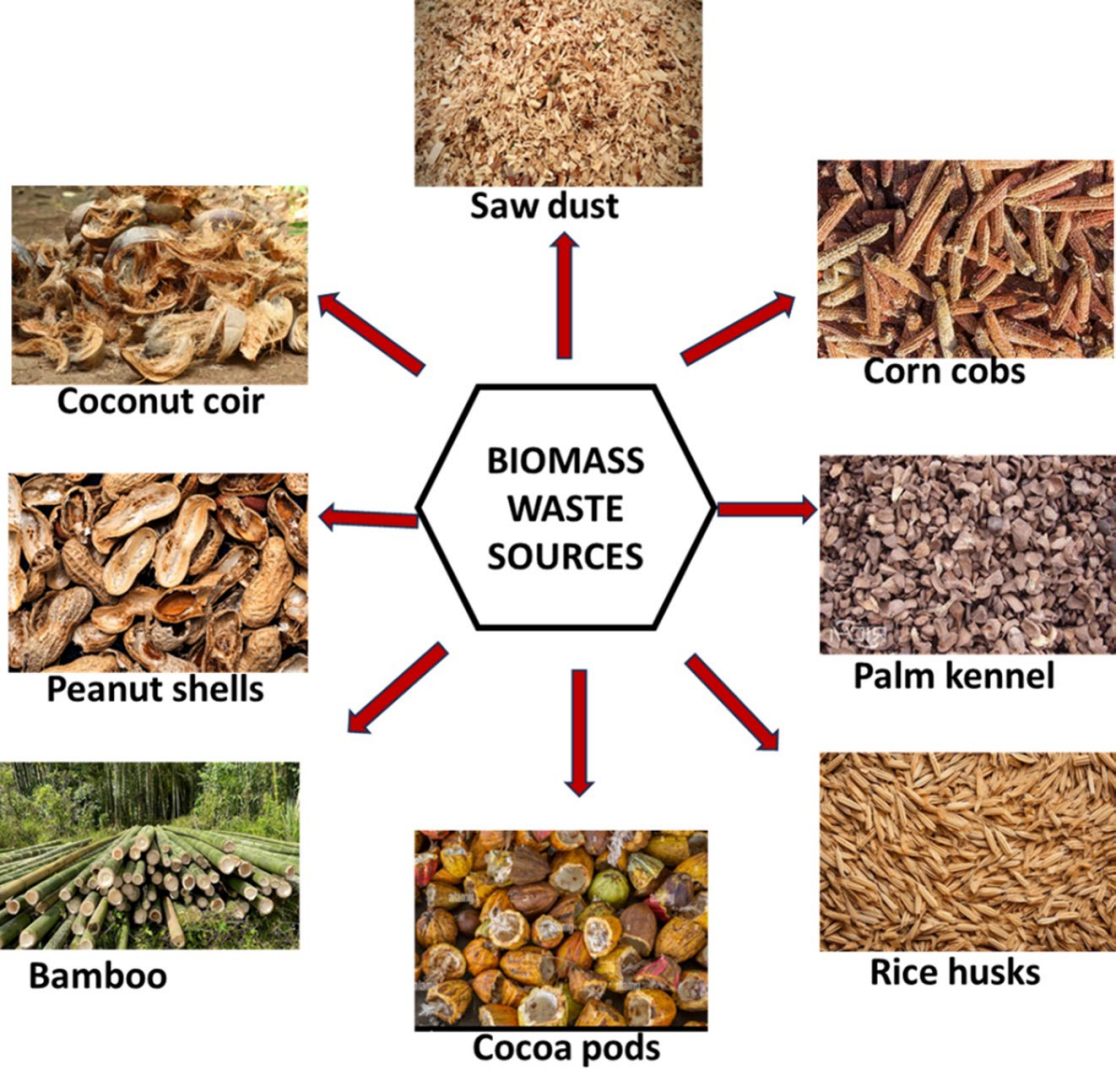
Sources of biomass waste from wood, food, and agriculture

The exact structure of activated charcoal remains a topic of debate. Marsh and Rodríguez–Reinoso examined more than 15 models for the structure, which is mentioned in their book released in 2006, but they were unable to determine which was the most accurate [134]. Recent research suggests it contains heptagonal and pentagonal rings similar to the fullerene structure [134]. The choice of raw material and manufacturing process greatly influences its features, including the pore structure. Selecting the appropriate raw material, production technology, and processing conditions is crucial. Efforts are being made to explore novel raw resources like biomass waste from wood, food, agriculture, and other sources for carbonaceous adsorbent production (Fig. 9).

#### Activation of charcoal

##### Physical activation

The production of activated carbon involves heating source materials with gases, followed by an air-based combustion process that yields refined, polished AC [140]. This transformation occurs through established procedures such as:

(i) *Carbonization*: The carbonization process involves heating carbon-containing substances to between 600 and 900 °C in an atmosphere of inert gases such as nitrogen or argon, undergoing pyrolysis, and transforming into carbonized material.
(ii) *Activation/oxidation*: The activation process occurs when the material is subjected to oxidizing conditions (using steam or O_2_) at a temperature range of 600–1200 °C, followed by heat treatment at 450 °C for an hour using an air-filled muffle furnace [134].

##### Chemical activation

Chemical activation of the carbonaceous precursor is achieved through selective impregnation protocols utilizing diverse activating agents. The impregnation process incorporates either phosphoric acid (25% w/v), potassium hydroxide (5% w/v), sodium hydroxide (5% w/v), calcium chloride (25% w/v), or zinc chloride (25% w/v) as the primary activation catalyst. Subsequently, the chemically treated material undergoes controlled pyrolysis under anaerobic conditions at moderately high temperatures (500–700 °C), facilitating the elimination of tarry byproducts while promoting the development of an optimized porous framework [134]. The thermal treatment induces structural modification of the carbon matrix, thereby facilitating molecular reorganization and subsequently micropore evolution.

Comparatively, the chemical approach proves more beneficial than physical activation due to its reduced temperature requirements, better quality consistency, and shorter processing times, which together prevent tar accumulation. This process requires heating various wood-derived materials, such as chips and sawdust, to elevated temperatures. The chemical activation typically employs potassium-based compounds (K_2_CO_3_, KOH), zinc chloride, or phosphoric acid as activating agents [141–143]. During activation, multiple reactions take place simultaneously: materials undergo condensation reactions, water molecules are removed, carbonization proceeds, polymers form through linking reactions, and oxidative processes occur.

#### Mechanism of dye removal

Activated charcoal (activated carbon) is a highly effective adsorbent for organic dye removal, primarily due to its exceptionally high specific surface area (often exceeding 1000 m²/g and reaching over 3000 m^2^/g in some cases) and well-developed porosity, featuring a network of micropores and mesopores that facilitate physisorption and pore filling [142]. The removal mechanism is dominated by van der Waals forces, hydrophobic interactions, and π-π stacking between the graphitic domains of the carbon and aromatic dye molecules [143]. Surface functional groups, introduced during activation, can enhance electrostatic interactions with charged dyes, while the amorphous carbon structure provides abundant defect sites for molecular trapping [144]. In the context of green synthesis, activated charcoal derived from agricultural waste (e.g., coconut coir, palm kernel, date palm) via sustainable activation methods (e.g., using bio-based activating agents or solar-powered pyrolysis) offers an eco-friendly and low-cost solution for wastewater treatment. Its proven efficiency, recyclability, and scalability make activated charcoal a benchmark material for dye adsorption technologies [145, 146].

### Carbonaceous agricultural waste products for organic dye removal from wastewater

Carbonaceous agricultural waste products have received a lot of attention as low-cost and environmentally friendly adsorbents for removing organic dyes from aqueous solutions. Based on their abundant availability, renewability, and good adsorption capabilities, these waste materials produced from various agricultural operations provide attractive alternatives to standard adsorbents [147, 148]. Agricultural activities generate significant volumes of waste materials, such as rice husks, sugarcane bagasse, coconut shells and coir, palm kennels, date palm seeds and fruit peels, which are mostly made up of carbonaceous components [149]. Carbonaceous agricultural waste materials have inherent porosity frameworks and functional groups that, through physical and chemical interactions, can effectively adsorb organic dyes [150–152]. The use of such waste products not only decreases the environmental impact of agricultural waste disposal but also provides a long-term solution for wastewater treatment [153].

Biomass-derived carbons from palm kernel, coconut coir, and date palm contain lignocellulosic materials rich in hydroxyl and carboxyl groups, which promote adsorption of dyes via hydrogen bonding and electrostatic interactions. Their porous framework allows for effective physical trapping and surface adsorption of dyes. These natural adsorbents are attractive for sustainable wastewater treatment due to their low cost, abundance, and environmental friendliness [154].

#### Coconut coir

Coconut coir is a fibrous fibre generated from coconut husk and is abundant as an agricultural waste product. Their vast availability, renewability, and good adsorption qualities make them appealing alternatives to conventional adsorbents. Various synthesis methods, such as activation, carbonization, and chemical modifications, have been employed over the years to convert coconut coir into carbonaceous compounds suitable for dye adsorption.

Sharma et al. [155] investigated the adsorption of methylene blue dye from wastewaters using coconut coir-based activated carbon. The dried coir pith was carbonized at 700 °C for 1 h in an indigenous experimental setup employing a tubular muffle furnace. The activated carbon synthesized had an adsorption capacity of 15.59 mg/g. Aljeboree et al. [156] also studied the efficacy of coconut husk-based activated carbon as an efficient adsorbent for the removal of crystal violet from aqueous solutions. The coconut husk was impregnated with concentrated H_2_SO_4_ and dried in an oven at 80˚C for 24 h. It was then activated in a hot air oven at 400˚C for 2 h. The carbonized material was rinsed with distilled water to remove free acid until the pH of the activated carbon reached 6.6–6.8 before being dried at 105 °C. The amount of dye uptake increased with dye concentration, pH, temperature and contact time.

Coconut coir dust was used as a low-cost adsorbent for methylene blue dye removal from aqueous solutions by Etim et al. [157]. The synthesized coconut coir dust exhibited a monolayer adsorption capacity of 29.50 mg/g as compared to different agricultural waste adsorbents used for methylene blue removal. Macedo et al. [158] studied the removal capacity of mesoporous activated carbon prepared from coconut coir dust for the adsorption of methylene blue and remazol yellow dyes. They discovered the adsorbent to be effective and efficient. The ability of activated carbon prepared from coconut husk with H_2_SO_4_ activation to remove maxilon blue and direct yellow from aqueous solutions was investigated by Aljeboree et al. [159]. The study demonstrated that both maxilon blue and direct yellow dyes adsorb well at acidic pH. Adsorption uptake was observed to increase with increasing starting dye concentration and contact time but decrease with increasing adsorbent dosage, particle size and system temperature. Researchers have used coconut-based adsorbents for water treatment in various modified forms, according to a review done by Bhatnagar et al. [160].

#### Date palm

In recent years, there has been a growing interest in the use of activated carbon derived from date palm fiber as an excellent adsorbent for the removal of dyes from aqueous solutions. Date palm fiber, an abundant agricultural waste material, provides a sustainable and cost-effective precursor to produce activated carbon with superior adsorption capacities. Date palm fibre has unique properties such as high carbon content, rich surface functional groups and innate porosity, making it an attractive precursor for the manufacture of activated carbon. The activation process, which includes physical or chemical treatments, increases the adsorption capacity and surface area of date palm fiber-based activated carbon, allowing a broader pollutant adsorption possibility.

Daoud et al. [161] experimented on the adsorption of activated carbons made from date palm rachis to remove reactive dye (bezaktiv red s-max) from an aqueous solution. The activated carbon was prepared using chemical activation by KOH as an impregnation agent at 800 °C. The maximum monolayer adsorption capacity (Q_m_) (mg/g) was observed to be 128.21 mg/g or the date palm rachis-based activated carbon in removing the reactive dye.

Chowdhury and his team published a review on the efficient removal of organic dyes from an aqueous environment using date palm-based activated carbon. They mentioned how the various activation methods influenced the adsorption of the organic dye. They claim that, in the case of physical activation, because of the smaller molecular size and faster diffusion rates, steam activation produced activated carbons (ACs) with larger pore size distributions and higher surface area than CO_2_ activation [162]. They concluded that compared to commercial AC, ACs made from date palm wastes have superior textural qualities and consequently a higher adsorption capacity towards a wide range of dyes.

Jabbar et al. [163] performed an experiment where an anionic eosin dye was removed from an aqueous solution using modified activated carbon made from date palm leaves (ACDPF). ACDPF was treated with a 10% w/v concentration of hydrogen peroxide (H_2_O_2_) as an oxidizing agent to improve its adsorption capabilities towards the removal of eosin yellow dye. According to the findings from this experiment, the maximum adsorption capacity was 217 mg/g, and the removal effectiveness of treated AC (ACDPF-2) was 99.78% compared to 98.5% for untreated AC (ACDPF-1). Alharbi and his research group investigated the use of mesoporous activated carbon made from various date palm sources, as well as a mixture for efficient dye removal. They subsequently went ahead to publish an extensive review of the recent methods in the production of activated carbon from date palm residues for the removal of textile dyes [164–166].

#### Palm kennel

Palm kernel, a byproduct of the palm oil industry, provides a conveniently available and renewable precursor to produce activated carbon with high adsorption capacity. Utilizing this byproduct aids in waste reduction and supports the agricultural sustainability of the palm oil industry. Palm kernel-based activated carbon, like other agricultural byproducts, has unique properties such as high carbon content, abundant surface functional groups, and inherent porosities, making it a viable option for pollutant removal. Similarly, physical or chemical activation increases the surface area and adsorption capacity of palm kernel-based activated carbon, allowing for effective dye removal from aqueous solutions.

The preparation, characterization, and pollutant removal using activated carbon derived from palm kernel shells were explored by Garcia et al. [167]. The adsorbents were synthesized by impregnating palm kernel shells (PKS) with ZnCl_2_ as an activating agent, then carbonizing them in an autogenous environment at 500 and 550 °C for 1 h. The findings of this research showed that the sample produced with a 1:1 PKS: ZnCl_2_ mixture, which was carbonized at 550 °C, had the greatest MB adsorption capacity (maximum absorption at equilibrium, q_max_ = 225.3 mg MB / g adsorbent). The kinetic analysis revealed that after 4 h of contact time, removals of more than 90% adsorptions were achieved at equilibrium.

Lee and Zaini [168] studied the adsorption of rhodamine B dyes on palm kernel shell-activated carbons. PKS-AC was created by activating ZnCl_2_ for 2 hours at 600 °C. ZnCl_2_- PKS-AC possessed a high surface area of 1365 m^2^ /g and a maximum rhodamine B adsorption of 108 mg/g.

Mehr et al. [169] explored the possibility of modelling the thermodynamics and kinetics of methyl violet 2B dye removal using palm kernel-activated carbon. The palm kernel AC was modified using sodium hydroxide. The dye removal percentages for modified and unmodified adsorbents were 98.85% and 91.08%, respectively. In comparison to the modified and unmodified adsorbents, the maximal adsorption capacities of methyl violet were 107.3 and 83.91 mg /g, respectively.

In a research published in the AIP conference proceedings, Xiang and Ghazi [170] reported on the removal of methylene blue from an aqueous solution using palm kernel-activated carbon. The PKS was carbonized at 400 °C for 1 h, then impregnated in KOH in a ratio of 1:1 for 15 min before being carbonized again at 800 °C for 15 min. The findings showed that at a lower flow rate (1 mL/min), adsorption was reported to be high, with 96.99% removal.

Jasri et al. [171] explored the viability of using microwave radiation-assisted K_2_CO_3_ activation of mesoporous activated carbon generated from mixed wastes of oil palm fronds and palm kernel shells for methylene blue dye removal. The mixed biowaste powders (OPFPKS) treated with K_2_CO_3_ were catalytically pyrolyzed in a modified microwave oven. The activated carbon version of the mixture was subsequently heated for 24 h at 100 °C. The greatest MB removal (99.6%) happened with the optimum condition being: dosage = 0.06 g, dye concentration = 10 mg/L, pH = 10 and time = 20 min. OPFPKSAC exhibited a good adsorption capacity of 331.6 mg/g. Various researchers have also researched the use of palm kernel-activated carbon and its modified forms for water treatment [172, 173].

## Water treatment techniques for organic dye removal using CBMs

The existence of trace amounts of organic dyes in most water bodies poses an important question about the efficacy of wastewater treatment procedures in eliminating active dye molecules. Many conventional removal approaches have been examined for the removal of organic dyes from the aquatic environment, including photocatalysis, adsorption, and membrane separation treatments [174–176].

### Adsorption technique

Adsorption is a surface phenomenon defined as the adhesion of molecules, ions, or atoms from dissolved solids, liquids, or gases to a surface [177]. The removal of organic dye compounds from wastewater by adsorption is regarded as a green, clean, and versatile method due to its easy design and operation, as it does not produce any toxic wastes as a by-product, and is capable of removing most forms of organic material [178, 179]. The interactions between adsorbate molecules and the absorbent material are defined and dependent on the adsorption phenomena. The adsorption capacity of a carbon-based adsorbent for chemical compounds is affected by the adsorbate’s nature, which includes the polarity, size, molecular weight, functional groups, pore size and structure, and solution conditions (pH, ionic strength, and temperature) [180]. The formation of an adsorbate on the surface of the adsorbent during the adsorption process is influenced by the movement of adsorbate molecules toward the adsorbent’s external boundary layer [179, 181].

Several research studies have investigated the adsorption of active dye chemicals from wastewater using various adsorbents. Several materials, such as polymeric materials, clay, carbonaceous materials, and other materials, have been reported in the literature as adsorbents for the removal of active dye compounds for water treatment [179, 182–185]. This review examines the adsorption efficacy of various carbon-based adsorbents for the removal of active dye compounds from wastewater.

Activated carbon (AC) is a well-known and widely utilized carbon-based adsorbent. It is an efficient material for active dye removal due to its high adsorption capacity, well-developed porosity, and diversified surface chemistry. Several investigations have been conducted to explore AC’s adsorption capabilities and processes. According to Foo and Hameed [186], AC exhibits superior adsorption capability for several dye compounds. They emphasized the impact of pH, temperature, contact time, adsorbent dosage, and initial dye concentration on adsorption performance. Chung and his team investigated the use of activated carbon derived from sucrose and melamine as a low-cost adsorbent for the removal of methylene blue in wastewater. They recorded a maximum adsorption capacity of 454.57 mg/g at an MB concentration of 250 mg/L [187]. Gohr et al. [188] explored the use of -COOH-activated carbon powder for the removal of methylene blue and crystal violet. The modified adsorbent recorded maximum adsorption of 123.75 mg/g and 120 mg/g for methylene blue and crystal violet, respectively.

Alhogbi et al. [189] researched on the removal of Congo red and rhodamine B dyes from wastewater using activated carbon from palm tree fibre waste. The study recorded a maximum adsorption capacity of 9.79 and 26.58 mg/ g at 30 min for Congo red dye and Rhodamine B dye, respectively. An investigation on the adsorption of Yellow 18 dye from aqueous solutions utilizing an activated carbon produced from sour cherry (*Prunus cerasus* L.) stone was done by Angin [190]. The study showed that the activated carbon exhibited an adsorption capacity of 5.76 mg/g at 318 K.

Carbon nanotubes (CNTs) have distinct structural properties such as a high aspect ratio and huge surface area, which make them appealing adsorbents for active dye elimination. CNT adsorption mechanisms have been extensively researched. Wang et al. [191] reviewed the adsorption behaviour of carbon nanotubes, emphasizing the importance of surface functional groups in increasing adsorption capacity. They further explored the implications of CNT characteristics on adsorption efficiency, such as diameter, length, and surface modification. Wu investigated MWCNT adsorption effectiveness for procion red MX-5B at varied pH and temperature levels. Without any alteration to the MWCNTs, the saturation adsorption capacity was recorded as 44.64 mg/g [192]. Foroutan et al. [193] researched on a CNT/MgO/CuFe_2_O_4_ magnetic composite for the removal of methyl violet and Nile blue. The maximum capacity of cationic dyes was determined to be 35 mg/g. They went on to test the adsorbent on real textile wastewater and achieved a removal efficiency of 74%. Several other CNT-based composites, such as CNT-chitosan, CNT–activated carbon fibre (ACF), CNTs-Fe_3_O_4_, CNTs-dolomite, CNTs-cellulose, and CNTs-graphene for the adsorption of dyes, were also reported [194–196].

Graphene and its derivatives have demonstrated significant potential as adsorbents for active dye molecule elimination because of their unique two-dimensional structure and remarkable adsorption properties. The alteration of the surface area of graphene with specific functional groups can be crucial since it enhances the area of interaction between graphene and the contaminants, resulting in high adsorption capacity [178]. Several research studies have been conducted to study the adsorption capability of graphene-based materials Chen et al. [197] and Chen et al. [198] investigated the adsorption capability of graphene oxide-based composites for rhodamine B and methylene blue removal, respectively, highlighting the impact of parameters such as solution pH, temperature, and adsorbent dosage on the adsorption process. They also explored the adsorption mechanisms involved in graphene-based adsorbents, such as π-π stacking and electrostatic interactions.

Abd-Elhamid et al. [199] studied the application of polyacrylonitrile/β-cyclodextrin/graphene oxide nanofibers composite as an efficient adsorbent for cationic dye removal. Using crystal violet (CV) as a model dye molecule, the adsorption activity of PAN/-CV/GO composite nanofibers at various GO concentrations was investigated. The results showed that the concentration of the GO affected the adsorption process [199]. Numerous researchers have explored the use of various graphene oxide materials as adsorbents for different dye removal [200–202]. Table 3 is a summary of carbon-based bio-materials that have been used as adsorbents.

**Table 3:** Various carbon-based bio-materials as adsorbents for different dye removal

Adsorbent	Method of activation	Initial conditions	Adsorption capacity, mg/g	Pollutant removed	Refs.
Date pits	Chemical	dose: 1 g/l, pH: 7.2, initial conc: 100 mg/L, Temp: 20 ℃	31.5	malachite green dye	[203]
Olive stones	Physical	dose: 1 g/l, pH: 10, initial conc: 125.5 mg/L, Temp: 35 ℃	714 and 769 for black and green olive stones resp.	Methylene blue	[204]
Cherry stones	Chemical	dose: 0.5–5 g/l, pH: 11, initial conc: 100 mg/L, Temp: 30 ℃	283.30	Yellow 211	[205]
Jujube Seed	Chemical	dose: 1 g/l, pH: 7.2, initial conc: 10–50 mg/L, Temp: 30 ℃	75.76	Congo-Red	[206]
Tamarind seeds	Chemical	dose:1–3 g/l, pH: 2–12, initial conc: 5–50 mg/L, Temp: ---	142.12 and 94.45 resp.	Methylene blue and Methyl orange	[207]
Pomegranate peels	Chemical	dose: 0.2–3 g/l, pH: ---, initial conc: 25 mg/L, Temp: 25 ℃	384.61	Methylene blue	[208]
Palm tree fibre waste	Chemical	dose: 0.1 g/l, pH: 3–11, initial conc: 50–400 mg/L, Temp: ---	of 9.79 and 26.58 resp.	Congo red and rhodamine B	[189]
Pistachio shell	Physical	dose: 1 g/l, pH: 9, initial conc: 100 mg/L, Temp: room	Batch mode-21.834 Column mode-41.77	Basic blue 41 dye (BB 41)	[209]
Coconut shell	Chemical	dose: 0.5 g/l, pH: 4–9, initial conc: 250 mg/L, Temp: 30 ℃	166.7	Methylene blue	[210]
Waste orange and lemon peels	Chemical	dose:0.01–0.8 g/L, pH: 2–10, initial conc: 50–200 mg/L, Temp: ---	38 and 33 resp.	Methyl orange and methylene blue	[211]
Waste tea	Chemical	dose:0.2 g/L, pH: 2–12, initial conc: 50–350 mg/L, Temp: 30–50 ℃	203.34	Acid blue 25	[212]

#### Regeneration and reuse of CBMs after adsorption

Numerous methods have been developed to regenerate carbonaceous materials, but these often fail to fully restore the original adsorption capacity or cause significant material loss. Sometimes, the regeneration phases are difficult and can lead to adsorbent loss [213]. Regeneration of adsorbents for reuse is essential for lowering operational costs and maintaining sustainable systems [214]. Conventional regeneration techniques fall into three main categories: physical, chemical, and biological [215]. Regeneration is favored due to the environmental and economic drawbacks of disposal and carbon replacement. Regeneration is becoming increasingly important in adsorption for water treatment, necessitating new, alternative, and environmentally friendly regeneration methods [216]. The versatility of carbon-based adsorbents has inspired chemical, physical, and biological regeneration methods that can restore at least 80% of their initial dsorption capacity under optimal conditions. Regenerated materials maintain adsorption efficiency over multiple cycles, and their surface properties remain largely unchanged [217]. The selection of appropriate regeneration agents and techniques enhances dye desorption without attacking the adsorbents.

Thermal regeneration of carbon-based adsorbents is a viable approach for recovering adsorbent activity after dye adsorption. Physical regeneration has increasingly been identified as a promising technique capable of removing surface-adsorbed organic contaminants while simultaneously enhancing the initial textural properties of carbons that have experienced clogging or fouling during usage [218] Thermal regeneration extends the service life of activated carbons, reducing the economic and environmental burdens associated with frequent adsorbent replacement. The mechanisms underpinning the efficacy of physical regeneration methods derive from their ability to restore pore accessibility and surface chemistry to near-original conditions, thereby facilitating sustained adsorption performance upon reuse. In the context of dye removal applications, the thermal treatment employed during regeneration ensures the substantial desorption and decomposition of adsorbed dye molecules, ultimately preserving the integrity of the adsorbent structure and maintaining its capacity for subsequent reuse cycles [219].

Although activated carbons saturated with the acidic dye displayed a superior scaling factor to the.

starting material, the efficiency of regeneration decreased in comparison with those saturated with.

the basic dye. The adsorption capacity of microwave-regenerated samples after three cycles reached 192–240 mg/g for the basic dye and 154–175 mg/g for the acidic dye. Modeling of adsorption equilibrium for textile dyes onto both raw and microwave-regenerated activated carbons indicated multilayer adsorption of large dye molecules on microporous surfaces [220].

Biological regeneration offers an alternative strategy that can restore the adsorption capacity of carbon-based materials under mild conditions, minimizing fouling and maintaining sorption efficiency. Particularly when dealing with organic contaminants in water treatment, biological regeneration is a beneficial technique for recovering the adsorption capacity of carbon-based materials like activated carbon since it works in moderate circumstances, reduces fouling, and preserves sorption effectiveness. This method efficiently cleans the adsorbent and permits its reuse by using microbial activity to break down adsorbed organic materials [221].

The surface functionality of the carbon nanotubes, in particular, the presence of amine groups of long alkyl chains, which generate stronger electrostatic interactions between the MWNTs and the MB molecules [222], combined with the large specific surface area and porosity, provides an enhancement of the equilibrium MB uptake. Processing of used activated carbons saturated with textile dyes by microwave and conventional thermal regeneration methods enables the reusability of carbon-based adsorbents for dye removal [220]. The evolution of material characteristics and adsorption performance was examined through thermal, dielectric, and textural analysis. An adsorption—desorption study was conducted using batch tests with two commercial activated carbons and two targeting textile dyes of widely differing molecular sizes. Dye solutions were prepared in distilled water with three initial concentrations up to 1500 mg/L. Subsequent regeneration trials included nine microwave and conventional thermal cycles at temperatures reaching 600 °C in nitrogen. The latter featured heating periods of 1–12 h at 300, 400, and 500 °C, as representative maximum heating temperatures. Waste loaded with a mixture of methylene blue, acridine orange, and rhodamine B was converted into an organics-in-organics nanohybrid for the rapid and selective removal of harmful dyes from wastewater [223]. The dye intercalated in the interlayer spaces of natural layered silicate can be transformed into carbon by in situ confined conversion. Acid activation simultaneously generates structural defects that create a wealth of adsorption sites, allowing adsorption to proceed effectively. The as-prepared nanohybrid exhibits extraordinary adsorption performance towards multiple classes of dye pollutants, achieving removal efficiencies exceeding 99% at low concentrations below 10 mg/L. Adsorption behaviour conforms to the Langmuir isotherm and pseudo-second-order kinetic models, consistent with monolayer chemisorption on a homogeneous surface. The nanohybrid maintains a dye removal efficiency above 93% after five regeneration cycles. Preparation from dye-loaded clay waste represents a sustainable and economic way to develop new adsorbents, with important implications for practical wastewater purification [224, 225].

### Photocatalysis

The availability of clean and safe water is a fundamental necessity for the sustenance of life and the well-being of our planet [226]. However, the alarming increase in water pollution due to industrialization, urbanization, and agricultural activities has posed a severe threat to global water resources [227, 228]. Traditional water treatment methods have proven insufficient to cope with the scale and complexity of emerging contaminants, emphasizing the urgent need for innovative and sustainable water purification technologies [85]. In recent years, photocatalytic water treatment using carbon-based materials has emerged as a promising and cutting-edge approach to address this pressing environmental challenge [229].

Carbon, in its various forms, has garnered immense attention in the field of photocatalysis due to its unique electronic structure, high surface area, and exceptional catalytic properties. Carbon-based materials such as graphene and its derivatives, carbon nanotubes (CNTs), activated charcoal, graphite, and carbon nitride, possess inherent photocatalytic activity and offer the advantage of being abundant, cost-effective, and environmentally friendly [230]. The potential of these materials to harness solar energy and convert it into powerful oxidants for water purification has gathered noteworthy interest from researchers and environmental engineers alike.

**Fig. 10:**
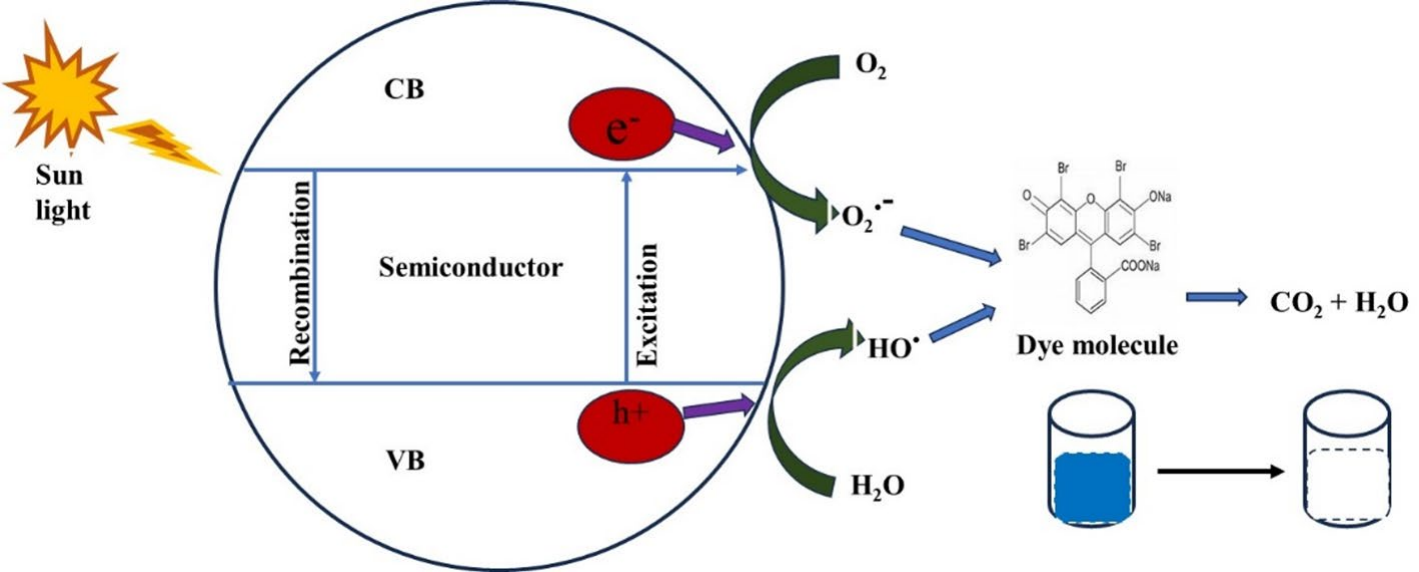
Schematic diagram for the photocatalysis process

The concept of photocatalysis involves the use of light energy to initiate chemical reactions on the surface of a photocatalyst, which can lead to the degradation of organic pollutants [231]. When exposed to light, carbon-based photocatalysts generate electron-hole pairs, and these photoinduced charge carriers facilitate redox reactions, resulting in the production of highly reactive species such as hydroxyl radicals (•OH), superoxide radicals (•O^2−^) and hydrogen peroxide (H_2_O_2_) [232]. These reactive oxygen species exhibit strong oxidation potential and efficiently transform harmful contaminants into harmless byproducts, thereby purifying the water (Fig. 10).

The importance of using carbon-based photocatalysts for water treatment lies in their capacity to use solar energy, a nearly limitless and renewable resource for environmental restoration. Additionally, carbon-based photocatalysts can purify enormous quantities of water on a large scale, potentially providing a solution to the world’s water crisis because of their scalable and sustainable nature [233]. The incorporation of carbon-based materials into water treatment technologies can be crucial as we move toward a more sustainable future in guaranteeing that future generations will have access to clean water.

In this section, the recent advancements in photocatalytic water treatment using carbon-based materials will be explored, examining their unique properties, underlying mechanisms, and potential applications. The review will delve into the various strategies employed to enhance the photocatalytic performance of carbon-based materials, such as element doping and forming composite materials with other semiconductors or metal oxides. Recently, a study was conducted by Ismail et al. [234] on the synthesis of α-Fe_2_O_3_/CNTs nanohybrids (with varied amounts of CNT) for photocatalytic degradation of Bismark Brown R dye using iron (III) acetylacetonate and MWCNT as precursors. The reported degradation efficiency increased from 83 to 98% with increasing CNT content from 0 to 50%, with 50% CNT being the optimum amount. This enhanced photoactivity was attributed to the combined effect of CNT and α-Fe_2_O_3_ and the suppression of electron-hole recombination at the interfaces of Fe_2_O_3_/CNT. In another report, Palanisamy et al. [111] used the wet impregnation method to produce a fantastic g-C_3_N_4_/α-Bi_2_O_3_/MWCNT nanocomposite. They assessed the photocatalytic performance of the nanocomposites against mixed dyes (Rh B and MB) and recorded about 94% decomposition efficiency. The reason for this remarkable performance was similar to that of Ismail et al. [234]. On the other hand, the effects of RGO addition on the TiO_2_’s photocatalytic properties have been studied by Yu and coworkers [235] using MO dye. They obtained a maximum degradation efficiency of about 97% within 90 min for the optimal amount of RGO in TiO_2_, which was found to be 6% of the mass of GO precursor relative to that of titanium sulphate and realized a decline in the efficiency beyond the optimum value. This decline was ascribed to the fact that excess RGO hindered the photon energy intake by TiO_2_. It is clearly evident that the role of carbon-based nanomaterials in the area of photocatalytic removal of organic dyes from water cannot be underestimated. Table 4 gives a summary of some of the recent reports on the application of carbon-based materials on the photocatalytic degradation of dyes.

**Table 4:** Some recent reports on the application of carbon-based materials on the photocatalytic degradation of dyes

Photocatalyst & dosage	Source of carbon	Organic dye & conc. (mg/L)	pH	Reaction time (min)	Efficiency (%)	Refs.
TiO_2_-bleached date palm fibers (BDPF)(2 g)	Date palm leaves	MB /50 CR /50	6 4	180	97.95 90.57	[236]
g-C_3_N_4_/Co/ZnO (20 mg)	Melamine	MB/15 CV/15 RhB/15	– – –	120	96.30 74. 50 75.14	[237]
Zn/CNT (100 mg)	MWCNT	CR/25	–	70	97.70	[238]
MWCNTs/Bi_2_S_3_ (50 mg)	MWCNT	MB/15	7	150	90.75	[239]
G-ZnO (50 mg)	Graphite powder	Rh-B MB MO	–	90 105 120	100.00 100.00 100.00	[240]
Ce^3+^-g-C_3_N_4_(120 mg)	Urea	MB/15	–	240	99.00	[241]
Fe-g-C_3_N_4_/ Ag_3_VO_4_/ CNT (0.5 g)	Melamine, CNT	2,4-DMP	6	140	99.00	[242]
HEC/SiO_2_/g-C_3_N_4_(50 mg)	g-C_3_N_4_	MB/30	–	60	94.60	[243]
Fe–TiO_2_/AC(1 g/L)	Palm empty fruit bunch	MG/100	3–4	45	97.00	[244]
ZnO/AC (20 mg)	Parthenium weed	MG/10 CR/10 MB/10	– – –	480	93.00 98.00 99.00	[245]
ZnO-SWCNT (130 mg)	SWCNTs	MB/0.25 g/L	–	120	100.00	[246]
DWCNT/N, Pd TiO_2_(100 mg)	DWCNT	EY/100	–	180	99.87	[247]
F-TiO_2_(B)/SWCNT (10 mg) F-TiO_2_(B)/MWCNT (10 mg)	SWCNTs MWCNTs	MG/100 MG/100	Neutral Neutral	120 120	91.83 88.89	[248] [248]

In summary, photocatalytic wastewater remediation using carbon-based nanomaterials represents a promising and eco-friendly approach to tackling water pollution. Their exceptional properties, combined with ongoing research efforts to enhance their performance, offer great potential for large-scale and sustainable water treatment solutions. As the field of photocatalysis continues to advance, carbon-based nanomaterials are poised to play a pivotal role in ensuring a cleaner and healthier environment for future generations. However, further interdisciplinary collaborations and comprehensive assessments are essential to realize the full potential of these materials and address the challenges for their practical implementation in wastewater treatment systems.

### Membranes

Membrane separation technology has been applied for biomass recycling in biotechnology and wastewater treatment. The implementation of membranes as a viable means of water treatment has progressed, using more advanced membranes made from new materials and employed in various configurations [249]. Membrane materials and processes that show promise to achieve high selectivity for water, ions, and small molecules, as well as the mechanisms involved, are highlighted in this section. In many cases, one membrane process is followed by another to produce water with high purity and quality for various purposes. One type of membrane may thus enhance the function of another to meet goals ranging from the disposal of wastewater to the production of drinking water from an unexpected source. Membranes can be divided into microfiltration, reverse osmosis, ultrafiltration, and nanofiltration [250].

#### Microfiltration

Microfiltration (MF) is a pressure-driven separation process, which is widely used in concentrating, purifying, or separating macromolecules, colloids, and suspended particles from solution. Microfiltration is a widely used membrane process for various applications such as wastewater treatment, juice clarification, protein separation, and bacteria separation. It can be explained as a water treatment process that is particularly suitable for the removal of suspended solids, especially bacteria, algae, and protozoa. However, microfiltration is less successful for the removal of dissolved contaminants such as natural organic matter [251]. Microfiltration membranes are available in both spiral-wound and flat-sheet configurations. Likewise, the membrane modules and filtration units can be customized to achieve specific application goals as required. This opens numerous possibilities for modified membrane systems for diversified applications [249]. Zang et al. [252] synthesized a diatomite hybrid microfiltration carbon membrane for oily wastewater treatment. In this work, the maximum oil rejection was achieved at 98.2% for 200 mg/L of oily wastewater. Also, in the work of Homem et al. [253], polyethersulfone microfiltration membranes (mPES) were modified with polyethyleneimine (PEI) and graphene oxide (GO) by-layer-by-layer self-assembly method via electrostatic interaction using a pressurised filtration system. The best performance of the membrane was achieved with blue corazol dye rejection of 97.8% and pure water permeability of 99.4 L m^− 2^ h^− 1^ bar ^− 1^.

#### Ultrafiltration

The ultrafiltration (UF) process uses membrane-based separation, where molecules move through a semipermeable barrier in response to pressure forces or differences in concentration. As a mechanical filtration technique, UF has proven its worth in water treatment applications, delivering excellent results whether used independently or integrated into larger treatment systems. Its combination of safety, cleanliness, cost-effectiveness, and powerful separation capabilities makes it ideal for tackling various water impurities [254]. Ultrafiltration membranes can be constructed from water-loving or water-repelling polymers. The process achieves nearly complete separation at relatively low pressures (1–2 bar), as osmotic pressure plays a minimal role. However, when treating oily wastewater, membrane performance suffers from fouling - tiny oil droplets accumulate on the surface and clog the pores. To combat this, improving the membrane’s water-attracting properties and resistance to fouling is crucial for optimal performance [255].

**Fig. 11:**
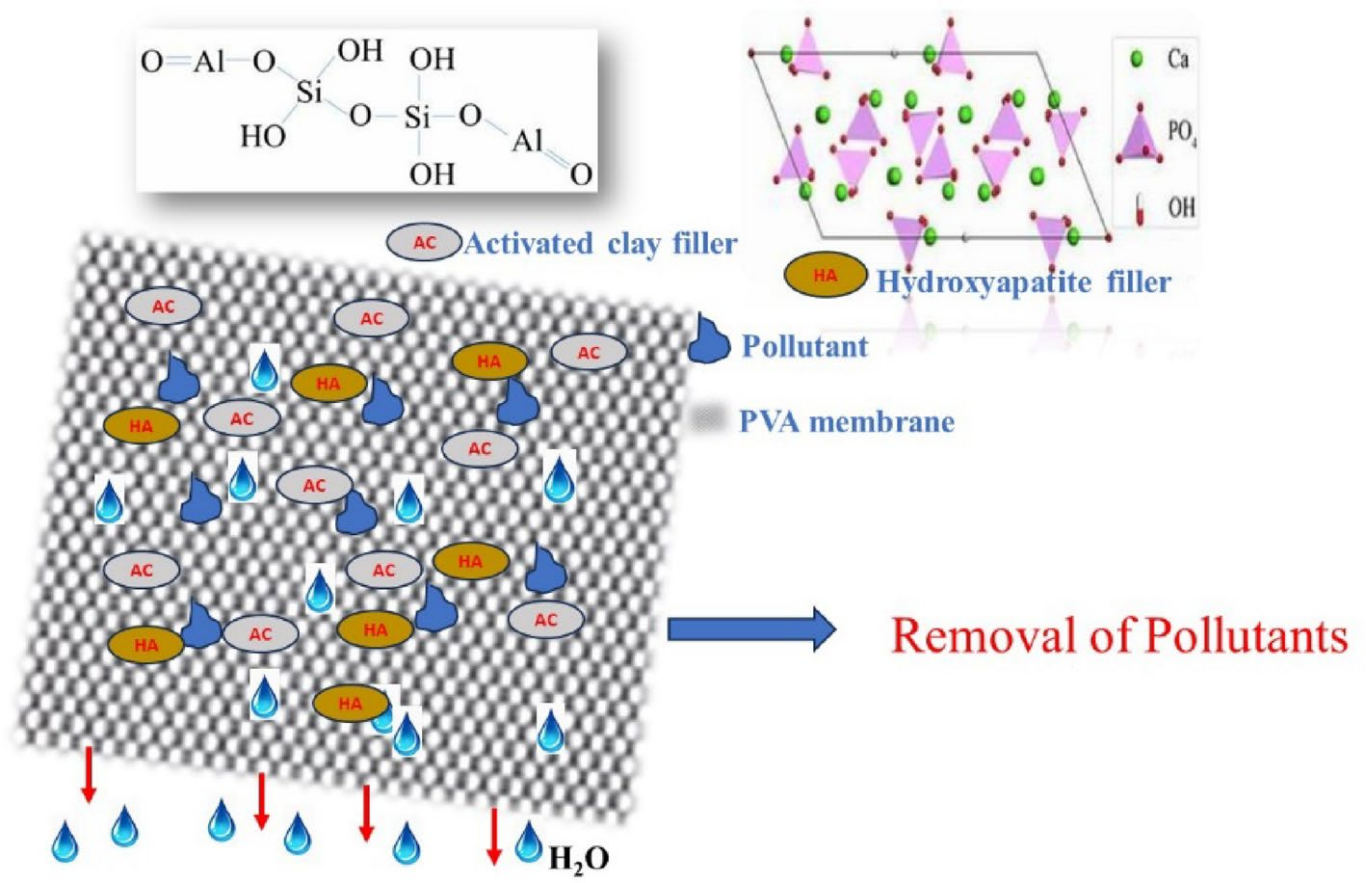
Ultrafiltration method using a PVA membrane [258]

Microfiltration shares many characteristics with ultrafiltration, as both rely on physical size-based separation mechanisms to capture particles. This contrasts with membrane gas separation, which functions through differential diffusion and absorption rates. Ultrafiltration membranes are characterized by their molecular weight cut-off (MWCO), and the technology encompasses two main variants: micellar-enhanced and polymer-enhanced ultrafiltration. Athanasekou et al. [256] developed an innovative water treatment system combining photocatalysis with ultrafiltration, powered by visible light. Their approach utilized a composite membrane made of partially reduced graphene oxide and titanium dioxide. The team created this hybrid system by depositing TiO_2_ nanoparticle-decorated graphene oxide sheets into ultrafiltration monolith pores using dip-coating methods. In another study, researchers developed a catalyst combining Co_3_O_4_ with carbon nanofibers of different types. They integrated this Co_3_O_4_@CNF catalyst with ultrafiltration membranes to activate peroxymonosulfate oxidation for treating wastewater that had already undergone initial treatment [257]. Building on this work, another research team created PVA-based membranes incorporating both activated clay and hydroxyapatite fillers (Fig. 11). These composite membranes proved highly effective, removing over 95% of methylene blue dye through the combined action of the PVA matrix and its mineral additives [258].

#### Reverse osmosis

Membrane processes, including reverse osmosis (RO), have been widely adopted for water treatment and reuse [259]. RO membranes lack discrete pores that penetrate the membrane and represent one end of the spectrum of commercially available membranes. RO system separates dissolved solutes (including single-charged ions, such as Na^+^, Cl^−^. etc.) from water via a semipermeable membrane that passes water in preference to the solute. RO can be described as a diffusion-controlled process in which the mass transfer of permeant through RO membranes, which is controlled by diffusion, is known as the solution–diffusion mechanism. In the solution–diffusion mechanism, permeants dissolve in the membrane material and then diffuse through the membrane [260]. The polymer material of RO membranes forms a layered, web-like structure, where water must follow a tortuous pathway through the membrane to reach the permeate side. RO uses pressured membranes for the treatment and desalination of brackish water, producing high-quality water. However, as with other membrane processes (i.e., nanofiltration, ultrafiltration, and microfiltration), the challenge of RO is the management of the concentrate generated from the filtration processes [259]. Inukai et al. [261] developed an advanced composite membrane for reverse osmosis by combining multi-walled carbon nanotubes with aromatic polyamide through interfacial polymerization. Their investigation revealed that incorporating MWCNTs at 15.5% by weight yielded optimal results, enhancing water flux and fouling resistance while protecting the membrane from chlorine-induced degradation.

#### Nanofiltration

The versatility of nanofiltration (NF) membranes extends across numerous sectors, with their primary application being water processing and desalination. These specialized membranes serve as crucial components in treating diverse water sources, from groundwater to wastewater, and can function as a preliminary step in desalination operations. Beyond water treatment, NF technology has found its way into pharmaceutical manufacturing, biotechnology processes, food production, and various non-water-based applications [262]. The emergence of nanofiltration as a preliminary treatment step marks a significant advance in desalination technology. NF systems excel at purifying water by removing multiple impurities, from microscopic organisms to minerals causing hardness, and partially filtering out dissolved salts [263]. Successful implementation requires careful modeling, especially using advanced predictive techniques based on Nernst-Planck principles. However, the use of such methods generally requires a prior characterization of membrane properties, which depends on extensive experimentation. The chemical structures and physical properties of nanofiltration membranes determine water permeability, solute selectivity, mechanical/thermal stability, and antifouling properties, which greatly influence the separation efficiency and operation cost in nanofiltration applications [264]. In the work of Wang et al. [265], Polyethersulfone/carbon nanotube-based mix matrix membranes (MMM) were successfully prepared by the phase inversion method for nanofiltration. The investigation centered on two carbon nanotube variants - one measuring 20 nm (CNT1) and another at 40 nm (CNT2) in diameter. The research team explored both the impact of nanotube size and the effects of varying CNT2 concentrations between 0.01 and 1 weight%. Their findings indicated that membranes incorporating the smaller CNT1 exhibited enhanced nanofiltration performance. At 0.1 wt% CNT2 content, the PES/CNT2 membranes achieved peak performance with water flux reaching 38.91 L/m^2^ h and Na_2_SO_4_ rejection of 87.25% under 4 bar pressure. The rejection efficiency followed the pattern: Na_2_SO_4_ > MgSO_4_ > NaCl. Table 5 gives a summary of various membranes with carbon fillers for various applications.

**Table 5:** Some published work on various membranes with carbon fillers for various applications

Membrane type	Carbon-based filler	Application	References
Conducting polymer electrolyte	Carbon-based nanotubes(CNT)	Energy storage, Energy harvesting	[266]
Si_3_N_4_ Composites	Carbon black	Industrial application	[267]
Polyurethane membranes	Carbon black, Carbon nanotubes	Water treatment and gas separation	[268]
Polyimide membranes	Carbon-based nanomaterials	Oil-Water separation, gas separation	[269]
HDPE Membranes	Carbon black (CB), Multi-Walled carbon nanotubes (MWCNT), Graphene oxide	Water treatment, oil-water separation	[270]
Carbon-Based membranes	Carbon nanotubes (CNTs), Graphene, Graphene Oxide	Water treatment, Oil-Water separation	[271]

### Carbon-Based materials hybrid treatment systems for water treatment

Water pollution represents a critical global challenge, necessitating the development of advanced and efficient treatment technologies [272]. Traditional single-technology approaches to wastewater treatment often face inherent limitations when confronting complex, multi-contaminant industrial effluents. However, the strategic integration of carbon-based materials (CBMs) into hybrid treatment systems has emerged as a transformative approach to overcome these limitations and achieve superior pollutant removal efficiency.

Carbon nanomaterials, including activated carbon, carbon nanotubes (CNTs), graphene, and quantum dots-have demonstrated exceptional performance in water treatment applications [273]. Activated carbon, derived from biowaste materials, presents an environmentally friendly and economically viable solution for water treatment, serving multiple functions including taste and odor control, adsorption of suspended metals, and removal of undesired pollutants [274]. CNTs, in particular, possess remarkably large surface areas and can effectively remove diverse contaminants including heavy metals, mycotoxins, antimicrobials, antibiotics, and even uranium during wastewater treatment [275]. When functionalized, these carbon-based materials exhibit enhanced removal capabilities and selectivity for specific pollutant classes.

The fundamental premise underlying hybrid treatment systems is that combining multiple treatment technologies, each with distinct removal mechanisms can leverage complementary advantages and achieve synergistic effects that exceed the performance of individual technologies [276]. Rather than relying on a single removal pathway, hybrid systems strategically integrate CBMs with complementary treatment modalities such as electrochemical processes, advanced oxidation processes (AOPs), membrane filtration, or biological treatment. This multi-barrier approach addresses the inherent limitations of single-mechanism systems: adsorption-only approaches lead to adsorbent saturation, photocatalysis alone may be limited by light penetration, and electrochemical systems require continuous energy input.

The incorporation of carbon-layered nanostructures into biopolymer-based membranes has showcased remarkable outcomes, including enhanced separation efficiency and extended operational lifetime [277]. These innovations demonstrate that CBMs are not merely passive adsorbents but can serve as active components in integrated treatment systems, facilitating electron supporting catalytic reactions, and providing structural support for biological communities.

### Application of carbon-based materials in real-world water treatment scenarios

Carbon-based materials have emerged as powerful tools in addressing global water treatment challenges, offering versatile solutions across various applications. This section examines how these materials are being applied in real-world settings, highlighting their performance, innovations, and future potential.

#### Municipal water treatment plants

Municipal water treatment facilities worldwide have incorporated carbon-based materials into their purification processes, with activated carbon being the most widely adopted. The Thames Water treatment plant in the UK exemplifies successful implementation, using granular activated carbon (GAC) filters to remove chlorinated compounds and pharmaceuticals from drinking water with removal efficiencies of 85–95% for chlorinated compounds and 70–80% for pharmaceuticals.

Biochar has emerged as a cost-effective alternative to traditional activated carbon in municipal applications. The city of Seattle has implemented a biochar-based water treatment system specifically designed to address stormwater runoff contamination, achieving removal efficiencies of 90% for heavy metals and 80% for other pollutants [278]. This application demonstrates biochar’s potential as a more sustainable option, particularly when derived from locally available waste biomass [279].

Recent innovations in municipal applications include electrochemically active carbon nanotube (CNT) filters that have demonstrated enhanced performance in removing emerging contaminants. Studies published through 2025 indicate these advanced filters can achieve removal rates exceeding 98% for pharmaceuticals and personal care products [280], addressing growing concerns about these persistent contaminants in municipal water supplies. The development of hybrid carbon-based materials and composites has led to enhanced performance, improved regeneration capabilities, and extended operational lifespans [281].

#### Industrial wastewater treatment

In industrial settings, carbon-based materials provide effective solutions for treating complex effluents containing dyes, heavy metals, and recalcitrant organic compounds. Carbon nanotubes and graphene oxide have gained significant traction in these applications due to their exceptional adsorption capacities.

A textile manufacturing plant in Bangladesh implemented a CNT-based treatment system that achieved 95% removal efficiency for dyes and 90% for other pollutants. Additionally, research conducted at the University of California demonstrated graphene oxide’s remarkable capacity to remove heavy metals from industrial wastewater, with reported removal efficiencies of 99% for lead and 95% for mercury [41].

Comparative studies published in 2024 showed that graphene oxide and activated carbon achieved 97% removal efficiency for bisphenol A (BPA), making these materials particularly valuable for industrial applications where endocrine-disrupting compounds are present [282]. Functionalized carbon nanotubes have also demonstrated enhanced capabilities for desalination and the extraction of industrial pollutants [283], with some applications reporting operational cost reductions of up to 30% compared to conventional treatment methods.

The last decade has seen dramatic progress in graphene-based composites as advanced adsorbents for application in wastewater treatment [284]. These composites combine the exceptional properties of graphene with other materials to enhance performance and target specific contaminants. Catalytic degradation using carbon nanotubes and graphene oxide can effectively remove toxic pollutants from wastewater without producing secondary waste [285], addressing a critical challenge in industrial treatment processes.

#### Decentralized systems

Carbon-based materials have proven particularly valuable in decentralized water treatment systems, especially in developing regions with limited infrastructure. A study conducted by researchers at the University of Nairobi demonstrated the effectiveness of biochar-based household water treatment systems in Kenya, achieving removal efficiencies of 99% for bacteria, 95% for viruses, and 90% for parasites [286].

Point-of-use water treatment systems often apply combinations of technologies incorporating carbon-based materials to achieve the removal of multiple contaminants or prevent biological contamination. Recent implementations in rural areas of Sub-Saharan Africa have demonstrated that locally-produced biochar filtration systems can provide sustainable access to clean water at approximately one-third the cost of imported treatment technologies, while maintaining comparable effectiveness [287].

Decentralized wastewater systems using carbon-based materials are increasingly being implemented in developing countries [288], serving as a cornerstone of efforts to enhance resource efficiency and improve the resilience of water infrastructure [289]. These systems are particularly valuable in rural or sparsely populated areas where centralized infrastructure is not economically feasible.

#### Cost-effectiveness and sustainability considerations

Systematic reviews comparing biochar and activated carbon with conventional wastewater treatment methods have revealed several economic advantages of carbon-based approaches [290]. Biomass-derived carbon nanocomposites offer cost-effective alternatives for wastewater treatment, particularly when sourced from local agricultural waste streams [291]. This circular economy approach not only reduces treatment costs but also addresses waste management challenges.

A 2025 comprehensive analysis of carbon material lifecycle impacts found that while advanced materials like graphene and CNTs currently have higher production energy requirements, their extended operational lifespan and superior contaminant removal efficiencies may result in favorable long-term sustainability profiles. Biochar derived from agricultural waste demonstrates the most favorable overall environmental footprint, particularly when produced using renewable energy sources [278, 292].

Modified biochar and activated carbon have demonstrated high adsorption efficiencies for emerging contaminants, such as microplastics. Meanwhile, CNTs and graphene, with their high carbon content and surface area, also show promising results. Carbon-based adsorbents, including granular activated carbon, biochar, and carbon nanotubes, are notably praised for their cost-effectiveness in addressing these challenging pollutants [293].

#### Regulatory frameworks and standards

The application of carbon-based materials in water treatment is increasingly guided by evolving regulatory frameworks. Strict standards led by the US EPA, WHO, and EU are accelerating the adoption of advanced activated carbon solutions to meet increasingly stringent water quality requirements [294]. These regulatory developments have spurred innovations in monitoring technologies and certification protocols specific to carbon-based treatment systems.

As of 2025, several countries have integrated specific provisions for carbon-based water treatment technologies into their water quality standards, recognizing the unique capabilities these materials offer for addressing both conventional and emerging contaminants. This regulatory recognition has facilitated wider implementation and created market incentives for further advancement of carbon-based water treatment solutions. The Bipartisan Infrastructure Law in the United States has allocated over $50 billion to improve drinking water, wastewater, stormwater, and nature-based infrastructure [295], creating opportunities for the implementation of advanced carbon-based treatment technologies. Additionally, the European Union has established new frameworks for urban wastewater collection and treatment [296], potentially expanding the application of carbon-based materials in municipal systems.

#### Challenges and limitations

The application of carbon-based materials in the removal of organic dyes from wastewater presents challenges and limitations that must be addressed to optimize effectiveness and scalability. One significant issue is the environmental impact associated with dye effluents, which not only contribute to the degradation of aquatic ecosystems but also pose health risks to human populations. Traditional methods for dye removal, while effective to various extents, often lack energy efficiency and cost-effectiveness, restricting their broader application [297]. This inefficiency is pronounced when considering certain biological, chemical, and physical technologies, which may require extensive energy inputs or costly operational processes. In particular, the reliance on biopolymer-based solutions, though promising due to their renewability and non-toxic characteristics, still faces scalability hurdles. While laboratory studies continue to demonstrate favorable outcomes with these materials, the transition from experimental to industrial applications is fraught with difficulties. Many of the methods employed for dye removal at lab scales, including those utilizing leaf-based adsorbents, require complex phase separation techniques such as centrifugation or filtration, which can significantly increase operational costs [298]. These complications diminish the practicality of utilizing leaf-based adsorbents on a larger scale, leading to concerns about their economic viability when deployed in wastewater treatment facilities. Moreover, the physical and chemical properties of carbon-based materials can vary significantly depending on the precursor source and activation methods; this inconsistency can impede their effectiveness as reliable adsorbents for different types of organic dyes. The challenge, therefore, is not merely in sourcing effective carbon-based materials but also in understanding how to modify and optimize these adsorbents for diverse contaminant profiles and varying operational conditions.

Although some advances have been made, several challenges persist in the application of carbon-based materials for dye removal. These include mass transfer limitations, selectivity issues in complex wastewater matrices, adsorbent regeneration difficulties, and economic feasibility for large-scale applications [299]. Additionally, the adsorption capacity of most adsorbents remains relatively low compared to the high concentrations of dyes in industrial effluents, necessitating continued research and development. Traditional adsorbents often fall short due to limited capacity and a lack of sustainability. Furthermore, there are challenges related to the efficient separation of adsorbents after treatment, which has led to research on nanomagnetic carbon-based adsorbents that can be more easily recovered [27, 300].

Consequently, while the potential for carbon-based materials in the removal of organic dyes from wastewater is significant, a multifaceted approach that tackles these challenges is essential for advancing their practical application in real-world scenarios.

#### Future directions in research

In the pursuit of more effective methodologies for the removal of organic dyes from wastewater, future research should prioritize the exploration and optimization of carbon-based materials, given their promising adsorption capabilities and diverse modifications. One area of focus is the integration of biopolymers such as cellulose, chitin, and chitosan into carbon-based composite materials. These biopolymers are abundant, renewable, non-toxic, and biodegradable, which aligns with contemporary sustainability goals in environmental management. Their inherent properties can be further enhanced through the development of advanced oxidation processes, which have shown substantial effectiveness in dye removal applications [297]. Future investigations should also explore the synergetic effects of combining these biopolymers with carbon-based materials, thereby improving the efficiency of removal processes and reducing the overall costs associated with conventional methods. Moreover, the utilization of alternative adsorbents, particularly those derived from natural sources, warrants further exploration. Research has suggested that leaf-based adsorbents, while primarily studied in lab-scale settings, show potential for scalable application [298].

The field of adsorption technology for dye removal prioritizes novel adsorbents obtained from waste materials, encouraging resource circularity while achieving superior selectivity and capacity in complex wastewater conditions. Recent reviews have emphasized the unceasing evolution of adsorbent materials, with activated carbons prepared from various residues showing promising results for organic dye removal. Biochar and unconventional sources like biomass-based sorbents show particular promise as cost-effective, sustainable options due to their low cost, sustainability, and availability [301, 302]. A review by Sriram and colleagues [303] found that biochar and modified multi-walled carbon nanotubes (MWCNTs) performed best at eliminating dyes from single-component solutions and, more significantly, real-world multi-component (binary and ternary) systems. A significant development is the move in emphasis toward complex effluents, which replicate real textile wastewater with different types of dyes. In the majority of earlier research, adsorbents were assessed against a single pollutant. Research needs to focus on materials that work well in these complex environments. Moreover, a major strategic objective is to create materials that work well at natural pH levels in order to reduce the energy and chemical usage related to pH adjustment, which is a major operational challenge in current systems.

In addition, future research should focus on the preparation and modification of next-generation carbon-based adsorbents to enhance their adsorption performance [304]. Development of carbon materials from waste sources, like carbon generated from polyethylene terephthalate (PET), should receive special attention because of their high surface area, which has shown considerable adsorption potential [305]. This approach addresses both environmental sustainability and cost-effectiveness concerns.

Sustainable synthesis methods minimize energy consumption and environmental impact during manufacturing, ensuring materials remain economically viable throughout their lifecycle. Surface engineering techniques optimize adsorbent chemistry and morphology, enhancing dye uptake through improved pollutant interactions. The development of biodegradable reusable materials capable of adsorbing and reducing dyes in aqueous media represents a significant advancement in sustainable adsorption technology [306]. Efficient regeneration processes enable repeated use without performance loss, reducing operational costs and waste generation.

However, the economic viability of such processes remains a significant challenge due to the complexities involved in phase separation techniques commonly employed, such as centrifugation and filtration. This necessitates a shift in research paradigm towards developing more cost-effective methodologies that simplify these processes without compromising treatment efficacy. Emphasis should also be placed on the economic analysis of these new technologies to ascertain their viability in industrial applications.

The future of carbon-based materials for the remediation of organic dyes in polluted wastewater through adsorption and photocatalysis holds considerable promise, particularly as advancements in material science and nanotechnology continue to progress. As identified by Khan et al. [307], the limitations of first-generation photocatalysts, such as their dependency on UV light and inefficacy in degrading a broad spectrum of dyes, have spurred research into more sophisticated second- and third-generation photocatalysts. These newer materials demonstrate improved charge separation and reaction kinetics, allowing for greater efficiency in dye degradation under visible light, therefore addressing both environmental and practical considerations in wastewater treatment. Continuing to enhance the design of these catalysts will play a crucial role in improving performance. For instance, the optimization of surface area and morphology can significantly influence the adsorption capacity, providing a pathway for enhanced degradation efficiency.

Additionally, the integration of immobilization technology in third-generation photocatalysts has opened avenues for catalyst regeneration, thereby fortifying the sustainability of the treatment process. Future developments are likely to focus on maximizing the utilization of natural sunlight, thereby improving the economic feasibility of photocatalytic treatments [308]. By making strides towards the optimization of reactor designs and integrating automated control systems, researchers will be able to streamline the photocatalysis process, leading to more effective wastewater reclamation solutions that can be implemented on a larger scale. Alongside these technical advancements, a concerted effort to explore the molecular mechanisms of photocatalysis will be critical in unlocking the full potential of carbon-based materials for dye degradation. With the ongoing research and innovative approaches tailored towards enhancing photocatalytic efficiencies, the environmental remediation of wastewater is poised for significant improvements, paving the way for cleaner and more sustainable industrial practices.

## Conclusion

This comprehensive review demonstrates that carbon-based materials (CBMs), including activated carbon, carbon nanotubes (CNTs), graphene, and biochar, etc., have emerged as highly promising technologies for organic dye removal from wastewater, with reported removal efficiencies ranging from 75 to 90% for carbon nanoparticles and up to 100% for metal oxide nanoparticle composites. However, while these materials have shown exceptional laboratory performance, the transition from bench-scale innovation to industrial-scale implementation remains critically underdeveloped.

Several transformative innovations have advanced the field substantially. First, the strategic integration of nanotechnology with chemical functionalization has fundamentally enhanced material performance. Surface modification through oxygen- and nitrogen-containing functional groups, combined with nanostructuring, has created materials with superior selectivity and capacity for diverse dye classes. Second, the emergence of agro-waste-derived CBMs, such as activated carbon from cocoa shells and agricultural byproducts, represents a paradigm shift toward circular economy principles, simultaneously addressing waste management and pollution remediation. Third, novel composite materials combining CBMs with metal oxides and other nanomaterials have demonstrated synergistic effects, expanding the mechanistic toolkit beyond simple adsorption to include photocatalytic degradation and magnetic separation. These innovations collectively represent a maturation of the field from single-material, single-mechanism approaches to integrated, multifunctional systems.CBMs represent a genuinely promising technology for addressing the persistent challenge of organic dye removal from wastewater. However, realizing their full potential for large-scale, sustainable industrial application requires moving beyond incremental improvements in laboratory performance toward systematic resolution of identified gaps. The field must transition from demonstrating “what works” in controlled settings to comprehensively understanding “why it works,” “how to scale it,” and “what it costs.” Only through such rigorous, multifaceted research efforts, combining fundamental innovation with practical validation, economic analysis, and regulatory consideration, can CBMs fulfill their promise as transformative solutions for sustainable wastewater treatment. The next decade will be critical in determining whether CBM technologies transition from laboratory curiosities to essential components of industrial wastewater treatment infrastructure.

## Data Availability

No datasets were generated or analysed during the current study.

## References

[1] Nagraj PK, Chaurasia SL, Bharati N, Sharma J, Kumar AM, Sivalingam. Degradation of dyes by fungi: an overview on recent updates. Microbe. 2025;6:100232. 10.1016/j.microb.2024.100232.

[2] Singh GB, Vinayak A, Mudgal G, Kesari KK. Azo dye bioremediation: an interdisciplinary path to sustainable fashion. Environ Technol Innov. 2024;36:103832. 10.1016/j.eti.2024.103832.

[3] Lellis B, Fávaro-Polonio CZ, Pamphile JA, Polonio JC. Effects of textile dyes on health and the environment and bioremediation potential of living organisms. Biotechnol Res Innov. 2019;3:275–90. 10.1016/j.biori.2019.09.001.

[4] Lin J, Ye W, Xie M, Seo DH, Luo J, Wan Y, Van der Bruggen B. Environmental impacts and remediation of dye-containing wastewater. Nat Rev Earth Environ. 2023;4:785–803. 10.1038/s43017-023-00489-8.

[5] Dutta S, Adhikary S, Bhattacharya S, Roy D, Chatterjee S, Chakraborty A, Banerjee D, Ganguly A, Nanda S, Rajak P. Contamination of textile dyes in aquatic environment: adverse impacts on aquatic ecosystem and human health, and its management using bioremediation. J Environ Manage. 2024;353:120103. 10.1016/j.jenvman.2024.120103. 38280248 10.1016/j.jenvman.2024.120103

[6] Kumar M, Mishra A, K Patel S, Kushwaha J, Singh S, Mishra V, Singh D, Singh V, S Giri B, R Singhania R, Singh D. Environmental impacts and strategies for bioremediation of dye-containing wastewater. Bioengineering. 2025;12:1043. 10.3390/bioengineering12101043. 41155042 10.3390/bioengineering12101043 PMC12561609

[7] Kushwaha A, Kushwaha R, Singh P, Mishra S. Impact of chemical dyes on human health and environment. Int J Home Sci. 2023;9:105–8.

[8] Kapanga PM, Nyakairu GWA, Nkanga CI, Lusamba SN, Tshimanga RM, Shehu Z. A review of dye effluents polluting African surface water: sources, impacts, physicochemical properties, and treatment methods. Discover Water. 2024;4:85. 10.1007/s43832-024-00129-2.

[9] Islam MM, Aidid AR, Mohshin JN, Mondal H, Ganguli S, Chakraborty AK. A critical review on textile dye-containing wastewater: ecotoxicity, health risks, and remediation strategies for environmental safety. Clean Chem Eng. 2025;11:100165. 10.1016/j.clce.2025.100165.

[10] Krause MJ, Bronstein KE. Estimating National sludge generation and disposal from US drinking water and wastewater treatment plants. J Clean Prod. 2024;453:142121. 10.1016/j.jclepro.2024.142121. 40151806 10.1016/j.jclepro.2024.142121 PMC11938657

[11] Boukarma L, Aziam R, Aboussabek A, El Qdhy S, Zerbet M, Sinan F, Chiban M. Novel insights into crystal Violet dye adsorption onto various macroalgae: comparative study, recyclability and overview of chromium (VI) removal. Bioresour Technol. 2024;394:130197. 10.1016/j.biortech.2023.130197. 38086462 10.1016/j.biortech.2023.130197

[12] Castillo-Suárez LA, Sierra-Sánchez AG, Linares-Hernández I, Martínez-Miranda V, Teutli-Sequeira EA. A critical review of textile industry wastewater: green technologies for the removal of Indigo dyes. Int J Environ Sci Technol. 2023;20:10553–90. 10.1007/s13762-023-04810-2. 10.1007/s13762-023-04810-2 PMC10041522 37360556

[13] Momina M, Qurtulen Q, Salimi Shahraki H, Ahmad A, Zaheer Z. Machine learning approaches to predict adsorption performance of sugarcane derived-carbon dot – based composite in the removal of dyes. Sep Purif Technol. 2024;351:127937. 10.1016/j.seppur.2024.127937.

[14] Abdullah TA, Nguyen B-S, Juzsakova T, Rasheed RT, Hafad S, Mansoor H, Al-Jammal N, Salman AD, Awad HA, Domokos E, Le PC, Nguyen V-H. Promotional effect of metal oxides (MxOy = TiO2, V2O5) on multi-walled carbon nanotubes (MWCNTs) for kerosene removal from contaminated water. Mater Lett. 2021;292:129612. 10.1016/j.matlet.2021.129612.

[15] Rezai B, Allahkarami E. Chap. 2 - Wastewater treatment processes—techniques, technologies, challenges faced, and alternative solutions, in: R.R. Karri, G. Ravindran, M.H. Dehghani, editors, Soft computing techniques in solid waste and wastewater management. Amsterdam, The Netherlands: Elsevier; 2021. pp. 35–53. 10.1016/B978-0-12-824463-0.00004-5.

[16] Ajiboye TO, Oyewo OA, Onwudiwe DC. Adsorption and photocatalytic removal of Rhodamine B from wastewater using carbon-based materials. FlatChem. 2021;29:100277. 10.1016/j.flatc.2021.100277.

[17] Samuel HS, Adeiza Okino I, Gideon O, Etim EE, Sustainable wastewater treatment: recent progress in the use of bio-waste-derived adsorbents for organic dye removal. Asian J Environ Res. 2024;1:137–51. 10.69930/ajer.v1i3.173.

[18] Abegunde SM, Idowu KS, Adejuwon OM, Adeyemi-Adejolu T. A review on the influence of chemical modification on the performance of adsorbents. Resour Environ Sustain. 2020;1:100001. 10.1016/j.resenv.2020.100001.

[19] Abdullah TA, Juzsakova T, Rasheed RT, Mallah MA, Salman AD, Cuong LP, Jakab M, Zsirka B, Kułacz K, Sebestyén V. V_2_ O_5_, CeO_2_ and their MWCNTs nanocomposites modified for the removal of kerosene from Water. Nanomaterials 2022;12. 10.3390/nano12020189 10.3390/nano12020189 PMC8778115 35055208

[20] Karzegar S, Abedi M, Salmani MJ, Askrishahi M, Babaei F, Salmani MH. Modification of agricultural waste carbon adsorbents with iron and iron oxide nanoparticles for heavy metals removal: a scoping review of the literature. J Environ Health Sustainable Dev. 2024;9:2405–15. 10.18502/jehsd.v9i4.17387.

[21] Loura N, Rathee K, Dhull R, Singh M, Dhull V. Carbon nanotubes for dye removal: a comprehensive study of batch and fixed-bed adsorption, toxicity, and functionalization approaches. J Water Process Eng. 2024;67:106193. 10.1016/j.jwpe.2024.106193.

[22] Varshan GSA, Namasivayam SKR, Sivasuriyan KS. Carbon nanodots as nanoadsorbents: a novel approach for dye-polluted effluent remediation. Environ Monit Assess. 2025;197:1082. 10.1007/s10661-025-14537-x. 40900418 10.1007/s10661-025-14537-x

[23] Alvez-Tovar B, Scalize PS, Angiolillo-Rodríguez G, Albuquerque A, Ebang MN, de Oliveira TF. Agro-Industrial waste upcycling into activated carbons: a sustainable approach for dye removal and wastewater treatment. Sustainability. 2025;17:2036. 10.3390/su17052036.

[24] Dada AO, Inyinbor AA, Atunwa BT, Gonuguntla S, Bello OS, Adekola FA, Pal U. Agrowaste-carbon and carbon-based nanocomposites for endocrine disruptive cationic dyes removal: a critical review. Biotechnol Rep. 2024;44:e00860. 10.1016/j.btre.2024.e00860. 10.1016/j.btre.2024.e00860 PMC11639365 39678013

[25] Abady MM, Mohammed DM, Soliman TN, Shalaby RA, Sakr FA. Sustainable synthesis of nanomaterials using different renewable sources. Bull Natl Res Cent. 2025;49:24. 10.1186/s42269-025-01316-4.

[26] Ali K, Zeidan H, Ben Amar R. Evaluation of the use of agricultural waste materials as low-cost and eco-friendly sorbents to remove dyes from water: a review. Desalin Water Treat. 2023;302:231–52. 10.5004/dwt.2023.29725.

[27] Satyam S, Patra S. Innovations and challenges in adsorption-based wastewater remediation: a comprehensive review. Heliyon. 2024;10. 10.1016/j.heliyon.2024.e29573. 10.1016/j.heliyon.2024.e29573 PMC11064087 38699034

[28] Shah IA, Bilal M, Almanassra IW, Ihsanullah I. A comprehensive review of graphene oxide-based membranes for efficient dye removal from water sources. Sep Purif Technol. 2024;330:125277. 10.1016/j.seppur.2023.125277.

[29] Sagadevan S, Fatimah I, Mathanmohun M, Lett JA, Al-Anber MA. Biowaste-derived carbon for wastewater treatment: a sustainable and cost-effective approach. Biomass Convers Biorefin. 2025;15:13323–45. 10.1007/s13399-024-06198-6.

[30] Khalajiolyaie A, Jian C. Advances in graphene-based materials for metal ion sensing and wastewater treatment: a Review. Environments. 2025;12. 10.3390/environments12020043

[31] Said AA, Lisa M, Rahmad RF. Recent advances in carbon-based adsorbent materials for ammonium removal from water. BIO Web Conf. 2025;156:2014. 10.1051/bioconf/202515602014.

[32] Cooper DR, D’Anjou B, Ghattamaneni N, Harack B, Hilke M, Horth A, Majlis N, Massicotte M, Vandsburger L, Whiteway E, Yu V. Experimental review of graphene. ISRN Condens Matter Phys. 2012;2012:1–56. 10.5402/2012/501686.

[33] Geim AK. Graphene: status and prospects. Science. 1979;324(2009). 10.1126/science.1158877. 10.1126/science.1158877 19541989

[34] Choi W, Lahiri I, Seelaboyina R, Kang YS. Synthesis of graphene and its applications: a review. Crit Rev Solid State Mater Sci. 2010;35:52–71. 10.1080/10408430903505036.

[35] Bhol P, Yadav S, Altaee A, Saxena M, Misra PK, Samal AK. Graphene-based membranes for water and wastewater treatment: a review. ACS Appl Nano Mater. 2021;4. 10.1021/acsanm.0c03439.

[36] An YC, Gao XX, Jiang WL, Han JL, Ye Y, Chen TM, Ren RY, Zhang JH, Liang B, Li ZL, Wang AJ, Ren NQ. A critical review on graphene oxide membrane for industrial wastewater treatment. Environ Res. 2023;223. 10.1016/j.envres.2023.115409. 10.1016/j.envres.2023.115409 36746203

[37] Wei Y, Zhang Y, Gao X, Ma Z, Wang X, Gao C. Multilayered graphene oxide membrane for water treatment: a review. Carbon N Y. 2018;139. 10.1016/j.carbon.2018.07.040.

[38] Tarcan R, Todor-Boer O, Petrovai I, Leordean C, Astilean S, Botiz I. Reduced graphene oxide today. J Mater Chem C Mater. 2020;8. 10.1039/c9tc04916a.

[39] Lowe SE, Zhong YL. Challenges of industrial-scale graphene oxide production. Graphene oxide. Wiley; 2016;410–31. 10.1002/9781119069447.ch13.

[40] Zhan M, Xu M, Lin W, He H, He C. Graphene oxide research: current developments and future directions. Nanomaterials. 2025;15:507. 10.3390/nano15070507. 40214552 10.3390/nano15070507 PMC11990175

[41] Anegbe B, Ifijen IH, Maliki M, Uwidia IE, Aigbodion AI. Graphene oxide synthesis and applications in emerging contaminant removal: a comprehensive review. Environ Sci Eur. 2024;36:15. 10.1186/s12302-023-00814-4.

[42] Jia K, Zhang J, Zhu Y, Sun L, Lin L, Liu Z. Toward the commercialization of chemical vapor deposition graphene films. Appl Phys Rev. 2021;8. 10.1063/5.0056413.

[43] Hassan MH, Sajjad H, Zubair M. Biomedical applications of graphene oxide: progress and challenges-a comprehensive review. Int Res J Educ Technol 2024;6:2581–7795.

[44] Huang H-H, Joshi RK, De Silva KKH, Badam R, Yoshimura M. Fabrication of reduced graphene oxide membranes for water desalination. J Memb Sci. 2019;572:12–9. 10.1016/j.memsci.2018.10.085.

[45] M.M. and A.K.N. Ismail Ismariza and Ramli, Graphene-Based hybrid materials and their applications, in: Gopinath Subash MM C. B. and, Ramli, editors. Hybrid-Nanomaterials: fabrication, characterization and applications, Springer Nature Singapore, Singapore, 2024: pp. 89–121. 10.1007/978-981-97-9022-7_6

[46] Safajou H, Mizwari ZM, Rostaminia A, Khojasteh H, Aspoukeh P, Mazhari M-P. Green synthesis and enhanced photocatalytic performance of rGO/ZnO/Fe_3_O_4_ nanocomposites: a sustainable approach to environmental remediation. J Fluoresc. 2024. 10.1007/s10895-024-04014-y. 39446180 10.1007/s10895-024-04014-y

[47] Kumari N, Kumar S, Chauhan P, Kaur GA, Kainthla I, Shandilya M. Environmentally sustainable techniques for rGO synthesis: focus on spun calcination and clean technology advances. J Inorg Organomet Polym Mater. 2025;35:699–723. 10.1007/s10904-024-03385-w.

[48] Wang Y, Liu Y, Zhang H, Duan X, Ma J, Sun H, Tian W, Wang S. Carbonaceous materials in structural dimensions for advanced oxidation processes. Chem Soc Rev. 2025;54:2436–82. 10.1039/D4CS00338A. 39895415 10.1039/d4cs00338a

[49] Adotey E, Kurbanova A, Ospanova A, Ardakkyzy A, Toktarbay Z, Kydyrbay N, Zhazitov M, Nuraje N, Toktarbaiuly O. Development of superhydrophobic reduced graphene oxide (rGO) for potential applications in advanced materials. Nanomaterials. 2025;15:363. 10.3390/nano15050363. 40072166 10.3390/nano15050363 PMC11901565

[50] Sadek R, Sharawi MS, Dubois C, Tantawy H, Chaouki J. Superior quality chemically reduced graphene oxide for high performance EMI shielding materials. RSC Adv. 2022;12:22608–22. 10.1039/D2RA02678C. 36105968 10.1039/d2ra02678c PMC9372871

[51] Li J, Salleh NA, Ahmad N, Alshoaibi A, Kheawhom S, Mohamad AA. Reduced graphene oxide for supercapacitor applications. Singapore: Springer Nature Singapore; 2025. 10.1007/978-981-96-4930-3.

[52] Dudding C, Jankhani S, Grebener L, Ali M, Segets D, Wiggers H, Pope MA. Improved graphene-based sodium-ion battery anodes using low surface area, low temperature reduced graphene oxide powders. Carbon N Y. 2025;244:120608. 10.1016/j.carbon.2025.120608.

[53] Tene T, Bellucci S, Guevara M, Romero P, Guapi A, Gahramanli L, Straface S, Caputi L.S., Vacacela Gomez C. Role of graphene oxide and reduced graphene oxide in electric double-layer capacitors: a systematic review. Batteries. 2024;10. 10.3390/batteries10070256.

[54] Fathy M, Hassan H, Hafez H, Soliman M, Abulfotuh F, Kashyout AEHB. Simple and fast Microwave-Assisted synthesis methods of nanocrystalline TiO2 and rGO materials for Low-Cost Metal-Free DSSC applications. ACS Omega. 2022;7:16757–65. 10.1021/acsomega.2c01455. 35601296 10.1021/acsomega.2c01455 PMC9118208

[55] Mohd Kaus NH, Rithwan AF, Adnan R, Ibrahim ML, Thongmee S, Mohd SF, Yusoff. Effective strategies, mechanisms, and photocatalytic efficiency of semiconductor nanomaterials incorporating rgo for environmental contaminant degradation. Catalysts. 2021;11. 10.3390/catal11030302

[56] Barzinmehr H, Mirza-Aghayan M, Boukherroub R. Mechanistic insights into the roles of graphene oxide and its derivatives in organic transformations – a review. Catal Reviews. 2024;66:1737–827. 10.1080/01614940.2023.2232643.

[57] Kumar M, Chowdhury S, Randhawa JK. Emerging trends in membrane-based wastewater treatment: electrospun nanofibers and reticular porous adsorbents as key components. Environ Sci (Camb). 2024;10:29–84. 10.1039/D3EW00119A.

[58] Xiao J, Lv W, Xie Z, Tan Y, Song Y, Zheng Q. Environmentally friendly reduced graphene oxide as a broad-spectrum adsorbent for anionic and cationic dyes via π–π interactions. J Mater Chem Mater. 2016;4:12126–35. 10.1039/C6TA04119A.

[59] Shaha CK, Al Mahmud MA, Saha S, Karmaker S, Saha TK. Efficient removal of Sparfloxacin antibiotic from water using sulfonated graphene oxide: kinetics, thermodynamics, and environmental implications. Heliyon. 2024;10. 10.1016/j.heliyon.2024.e33644. 10.1016/j.heliyon.2024.e33644 PMC11261116 39040378

[60] Nasir S, Hussein MZ, Zainal Z, Yusof NA. Carbon-based nanomaterials/allotropes: a glimpse of their synthesis, properties and some applications. Materials. 2018;11:1–24. 10.3390/ma11020295. 10.3390/ma11020295 PMC5848992 29438327

[61] Xiang Q, Cheng B, Yu J. Graphene-Based photocatalysts for solar-fuel generation. Angewandte Chemie - Int Ed. 2015;54:11350–66. 10.1002/anie.201411096. 10.1002/anie.201411096 26079429

[62] Keeling J. Graphite: properties, uses and South Australian resources. MESA J. 2017;84:28–41.

[63] Elamrawy N. Graphite structure and its mechanical properties, Master’s thesis. Milan, Italy: Politecnico di Milano; 2018.

[64] Robinson GR, Hammarstrom JM, Olson DW, Graphite US. Geol Surv. 2017;1:1–36.

[65] Simandl G.J., Paradis S, Akam C. Graphite deposit types, their origin, and economic significance. Br Columbia Geol Surv Paper. 2015;3:163–71.

[66] Al-Tohamy R, Ali SS, Li F, Okasha KM, Mahmoud YA-G, Elsamahy T, Jiao H, Fu Y, Sun J. A critical review on the treatment of dye-containing wastewater: ecotoxicological and health concerns of textile dyes and possible remediation approaches for environmental safety. Ecotoxicol Environ Saf. 2022;231:113160. 10.1016/j.ecoenv.2021.113160. 35026583 10.1016/j.ecoenv.2021.113160

[67] Zhang Y, Zhang Z, Han H, Zhang M, Wang H, Song H, Chen Y. Effective removal of organic dyes using the ultrasonic-assisted hydrothermal synthesis of nap zeolite doping Cu or Fe in Fenton-like oxidation systems. Sep Purif Technol. 2022;299:121767. 10.1016/j.seppur.2022.121767.

[68] Bal G, Thakur A. Distinct approaches of removal of dyes from wastewater: a review. Mater Today Proc. 2022;50:1575–1579. 10.1016/j.matpr.2021.09.119

[69] Alguacil FJ, Alonso M, Robla JI. Removal of hazardous organic dyes from liquid wastes using advanced nanomaterials. Int J Mol Sci. 2024;25:9671. 10.3390/ijms25179671. 39273617 10.3390/ijms25179671 PMC11396100

[70] Kusumlata B, Ambade A, Kumar S, Gautam, Sustainable Solutions. Reviewing the future of textile dye contaminant removal with emerging biological treatments. Limnological Rev. 2024;24:126–49. 10.3390/limnolrev24020007.

[71] Ali AE, Chowdhury ZZ, Devnath R, Ahmed MM, Rahman MM, Khalid K, Wahab YA, Badruddin IA, Kamangar S, Hussien M, Pallan KH, Mitra A. Removal of Azo dyes from aqueous effluent using Bio-Based activated carbons: toxicity aspects and environmental impact. Separations. 2023;10:506. 10.3390/separations10090506.

[72] Xin Y, Bai Y, Wu X, Zhang D, Ao W, Fang M, Huang Z, Yao Y. Adsorpt perform modified graphite synth dyes solutions mater. 2024;17:4349. 10.3390/ma17174349. 10.3390/ma17174349 PMC11395901 39274738

[73] Hoang NB, Nguyen TT, Nguyen TS, Bui TPQ, Bach LG. The application of expanded graphite fabricated by microwave method to eliminate organic dyes in aqueous solution. Cogent Eng. 2019;6. 10.1080/23311916.2019.1584939.

[74] Jin Q-Q, Zhu X-H, Xing X-Y, Ren T-Z. Adsorptive removal of cationic dyes from aqueous solutions using graphite oxide. Adsorpt Sci Technol. 2012;30:437–47. 10.1260/0263-6174.30.5.437.

[75] Praus P, Svoboda L, Ritz M, Troppová I, Šihor M, Kočí K. Graphitic carbon nitride: Synthesis, characterization and photocatalytic decomposition of nitrous oxide. Mater Chem Phys. 2017;193:438–46. 10.1016/j.matchemphys.2017.03.008.

[76] Liu J, Wang H, Antonietti M. Graphitic carbon nitride reloaded: emerging applications beyond (photo)catalysis. Chem Soc Rev. 2016;45:2308–26. 10.1039/c5cs00767d. 26864963 10.1039/c5cs00767d

[77] Aljuaid A, Almehmadi M, Alsaiari AA, Allahyani M, Abdulaziz O, Alsharif A, Alsaiari JA, Saih M, Alotaibi RT. Khan, g-C_3_N_4_-based photocatalyst for the efficient photodegradation of toxic Methyl orange dye: recent modifications and future perspectives. Molecules. 2023;28:1–15. 10.3390/molecules28073199. 10.3390/molecules28073199 PMC10096294 37049963

[78] Wang X, Zhou C, Shi R, Liu Q, Waterhouse GIN, Wu L, Tung CH, Zhang T. Supramolecular precursor strategy for the synthesis of Holey graphitic carbon nitride nanotubes with enhanced photocatalytic hydrogen evolution performance. Nano Res. 2019;12:2385–9. 10.1007/s12274-019-2357-0.

[79] Antil B, Kumar L, Reddy KP, Gopinath CS, Deka S. Direct thermal polymerization approach to N-Rich Holey carbon nitride nanosheets and their promising photocatalytic H2 evolution and charge-storage activities. ACS Sustain Chem Eng. 2019;7:9428–38. 10.1021/acssuschemeng.9b00626.

[80] Goettmann F, Fischer A, Antonietti M, Thomas A. Metal-free catalysis of sustainable Friedel-Crafts reactions: direct activation of benzene by carbon nitrides to avoid the use of metal chlorides and halogenated compounds. Chem Commun. 2006;4530–2. 10.1039/b608532f. 10.1039/b608532f 17283808

[81] Kumru B, Antonietti M. Colloidal properties of the metal-free semiconductor graphitic carbon nitride. Adv Colloid Interface Sci. 2020;283:102229. 10.1016/j.cis.2020.102229. 32795670 10.1016/j.cis.2020.102229

[82] Wang Y, Wang X, Antonietti M, Zhang Y. Facile one-pot synthesis of nanoporous carbon nitride solids by using soft templates. Chemsuschem. 2010;3:435–9. 10.1002/cssc.200900284. 20191634 10.1002/cssc.200900284

[83] Alhaddad M, Mohamed RM, Mahmoud MHH. Promoting visible light generation of hydrogen using a Sol-Gel-Prepared MnCo2O4@g-C3N4p-n heterojunction photocatalyst. ACS Omega. 2021;6:8717–25. 10.1021/acsomega.1c00697. 33817535 10.1021/acsomega.1c00697 PMC8015085

[84] Bai J, Lv W, Ni Z, Wang Z, Chen G, Xu H, Qin H, Zheng Z, Li X. Integrating MoS2 on sulfur-doped porous g-C3N4 Iostype heterojunction hybrids enhances visible-light photocatalytic performance. J Alloys Compd. 2018;768:766–74. 10.1016/j.jallcom.2018.07.286.

[85] Singh J, Arora A, Basu S. Synthesis of coral like WO_3_/g-C_3_N_4_ nanocomposites for the removal of hazardous dyes under visible light. J Alloys Compd. 2019;808:151734. 10.1016/j.jallcom.2019.151734.

[86] Sutanto N, Saharudin KA, Sreekantan S, Kumaravel V, Md H, Akil. Heterojunction catalysts g-C3N4/-3ZnO-c-Zn2Ti3O8 with highly enhanced visible-light-driven photocatalytic activity. J Solgel Sci Technol. 2020;93:354–70. 10.1007/s10971-019-05101-4.

[87] Hasija V, Sudhaik A, Raizada P, Hosseini-Bandegharaei A, Singh P. Carbon quantum Dots supported AgI /ZnO/phosphorus doped graphitic carbon nitride as Z-scheme photocatalyst for efficient photodegradation of 2, 4-dinitrophenol. J Environ Chem Eng. 2019;7:103272. 10.1016/j.jece.2019.103272.

[88] Raizada P, Sudhaik A, Singh P, Shandilya P, Gupta VK, Hosseini-Bandegharaei A, Agrawal S. Ag3PO4 modified phosphorus and sulphur co-doped graphitic carbon nitride as a direct Z-scheme photocatalyst for 2, 4-dimethyl phenol degradation. J Photochem Photobiol Chem. 2019;374:22–35. 10.1016/j.jphotochem.2019.01.015.

[89] Kang J, Jin C, Li Z, Wang M, Chen Z, Wang Y. Dual Z-scheme MoS2/g-C3N4/Bi24O31Cl10 ternary heterojunction photocatalysts for enhanced visible-light photodegradation of antibiotic. J Alloys Compd. 2020;825:153975. 10.1016/j.jallcom.2020.153975.

[90] Liu W, Zhou J, Yao J. Shuttle-like CeO2/g-C3N4 composite combined with persulfate for the enhanced photocatalytic degradation of Norfloxacin under visible light. Ecotoxicol Environ Saf. 2020;190:110062. 10.1016/j.ecoenv.2019.110062. 31838233 10.1016/j.ecoenv.2019.110062

[91] Liu J, Zhang T, Wang Z, Dawson G, Chen W. Simple pyrolysis of Urea into graphitic carbon nitride with recyclable adsorption and photocatalytic activity. J Mater Chem. 2011;21:14398–401. 10.1039/C1JM12620B.

[92] Zhu B, Xia P, Ho W, Yu J. Isoelectric point and adsorption activity of porous g-C3N4. Appl Surf Sci. 2015;344:188–95. 10.1016/j.apsusc.2015.03.086.

[93] Zhang M, Xu J, Zong R, Zhu Y. Enhancement of visible light photocatalytic activities via porous structure of g-C3N4. Appl Catal B. 2014;147:229–35. 10.1016/j.apcatb.2013.09.002.

[94] Ma C, Liao H, Deng Q, Yang F, Qin T. Preparation of CSS-BC/g-C3N4 composite and its adsorption study on methylene blue. ChemistrySelect. 2025;10:e00142. 10.1002/slct.202500142.

[95] Zeng Y, Liu X, Liu C, Wang L, Xia Y, Zhang S, Luo S, Pei Y. Scalable one-step production of porous oxygen-doped g-C3N4 nanorods with effective electron separation for excellent visible-light photocatalytic activity. Appl Catal B. 2018;224:1–9. 10.1016/j.apcatb.2017.10.042.

[96] Cao S, Low J, Yu J, Jaroniec M. Polymeric photocatalysts based on graphitic carbon nitride. Adv Mater. 2015;27:2150–76. 10.1002/adma.201500033. 25704586 10.1002/adma.201500033

[97] Fdez-Sanromán A, Lomba-Fernández B, Sanromán A, Pazos M, Rosales E. Enhancing stability and immobilization techniques for graphitic carbon nitride in photocatalytic applications. J Mol Liq. 2024;405:125005. 10.1016/j.molliq.2024.125005.

[98] Zhu J, Xiao P, Li H, Carabineiro SAC. Graphitic carbon nitride: synthesis, properties, and applications in catalysis. ACS Appl Mater Interfaces. 2014;6:16449–65. 10.1021/am502925j. 25215903 10.1021/am502925j

[99] Akhundi A, Zaker Moshfegh A, Habibi-Yangjeh A, Sillanpää M. Simultaneous Dual-Functional photocatalysis by g-C3N4-Based nanostructures. ACS ES&T Eng. 2022;2:564–85. 10.1021/acsestengg.1c00346.

[100] Luo Y, Wei X, Gao B, Zou W, Zheng Y, Yang Y, Zhang Y, Tong Q, Dong L. Synergistic adsorption-photocatalysis processes of graphitic carbon nitrate (g-C_3_N_4_) for contaminant removal: Kinetics, models, and mechanisms. Chem Eng J. 2019;375:122019. 10.1016/j.cej.2019.122019.

[101] Hebbar RS, Inamuddin AM, Isloor. Carbon nanotube- and graphene-based advanced membrane materials for desalination. Environ Chem Lett. 2017;15:643–71. 10.1007/s10311-017-0653-z.

[102] Manimegalai S, Vickram S, Raj S, Rohini K, Thanigaivel S, Manikandan S, Subbaiya R, Karmegam N, Kim W, Govarthanan M. Chemosphere carbon-based nanomaterial intervention and efficient removal of various contaminants from effluents – a review, chemosphere. 2023;312:137319. 10.1016/j.chemosphere.2022.137319 10.1016/j.chemosphere.2022.137319 36410505

[103] Han M, Dong T, Hou D, Yao J, Han L. Carbon nanotube based Janus composite membrane of oil fouling resistance for direct contact membrane distillation. J Memb Sci. 2020;607:118078. 10.1016/j.memsci.2020.118078.

[104] Rasheed T, Adeel M, Nabeel F, Bilal M, Iqbal HMN. Science of the total environment TiO_2_ / SiO_2_ decorated carbon nanostructured materials as a multifunctional platform for emerging pollutants removal. Sci Total Environ. 2019;688:299–311. 10.1016/j.scitotenv.2019.06.200. 31229826 10.1016/j.scitotenv.2019.06.200

[105] Aqel A, El-Nour KMMA, Ammar RAA, Al-Warthan A. Carbon nanotubes, science and technology part (I) structure, synthesis and characterisation. Arab J Chem. 2012;5:1–23. 10.1016/j.arabjc.2010.08.022.

[106] Qiu L, Ding F. Understanding Single-Walled carbon nanotube growth for chirality controllable synthesis. Acc Mater Res. 2021. 10.1021/accountsmr.1c00111.35252877

[107] Onyancha RB, Ukhurebor KE, Aigbe UO, Osibote OA, Kusuma HS, Darmokoesoemo H. A Methodical review on carbon-based nanomaterials in energy-related applications. Adsorpt Sci Technol. 2022 (2022) 1–21. 10.1155/2022/4438286

[108] Saifuddin N, Raziah AZ, Junizah AR. Carbon nanotubes: a review on structure and their interaction with proteins. J Chem. 2013;2013:1–18. 10.1155/2013/676815.

[109] Palanisamy G, Venkatesh G, Srinivasan M, Bhuvaneswari K, Elavarasan N, Vignesh S, Pazhanivel T, Shkir M, Hakami J, Lee J. α-Bi_2_O_3_ nanoparticle and multiwall carbon nanotube hybrid with protonated g-C_3_N_4_ nanosheets for superior photocatalytic performance towards the mixed organic contaminants. J Alloys Compd. 2022;922:166147. 10.1016/j.jallcom.2022.166147.

[110] Wu K, Yu J, Jiang X. Multi-walled carbon nanotubes modified by polyaniline for the removal of Alizarin yellow R from aqueous solutions. Adsorpt Sci Technol. 2018;36:198–214. 10.1177/0263617416687564.

[111] Calucho E, Parolo C, Rivas L, Álvarez-Diduk R, Merkoçi A. Nanoparticle-based lateral flow assays. In: Comprehensive analytical chemistry. 2020. pp. 313–39. 10.1016/bs.coac.2020.04.011.

[112] Ghiazza M, Vietti G, Fenoglio I. Carbon nanotubes: Properties, applications, and toxicity. In: Health and environmental safety of nanomaterials: polymer nancomposites and other materials containing nanoparticles. In: Njuguna J, Pielichowski K, Zhu H, editors. Cambridge, United Kingdom: Woodhead Publishing Ltd; 2014. pp. 147–74. 10.1533/9780857096678.3.147.

[113] Das R, Shahnavaz Z, Ali ME, Islam MM, Abd Hamid SB. Can we optimize Arc discharge and laser ablation for well-Controlled carbon nanotube synthesis? Nanoscale Res Lett. 2016;11:1–23. 10.1186/s11671-016-1730-0. 27864819 10.1186/s11671-016-1730-0 PMC5116021

[114] Kharlamova MV, Burdanova MG, Paukov MI, Kramberger C. Synthesis, Sorting, and applications of Single-Chirality Single-Walled carbon nanotubes. Materials. 2022;15:1–44. 10.3390/ma15175898. 10.3390/ma15175898 PMC9457432 36079282

[115] Ivanova N, Gugleva V, Dobreva M, Pehlivanov I, Stefanov S, Andonova V. Synthesis of carbon nanotubes by catalytic chemical vapor deposition. In: Saleh HE-D, El-Sheikh SMM, editors. Perspective of carbon nanotubes. London, UK: IntechOpen; 2016. pp. 1–13.

[116] Prasek J, Drbohlavova J, Chomoucka J, Hubalek J, Jasek O, Adam V, Kizek R. Methods for carbon nanotubes synthesis - review. J Mater Chem. 2011;21:15872–84. 10.1039/c1jm12254a.

[117] Shanmugam NR, Prasad S. Carbon nanotubes: Synthesis and characterization, in: J.E. Morris, editor, Nanopackaging: nanotechnologies and electronics packaging, 2nd ed., Springer International Publishing AG, 2018: pp. 575–596. 10.1007/978-3-319-90362-0

[118] Monthioux M, Serp P, Flahaut E, Razafinimanana M, Laurent C, Peigney A, Bacsa W, Broto J-M. Introduction to carbon nanotubes. In: Bhushan B, editor. Springer handbook of nanotechnology. Berlin, Heidelberg: Springer; 2004. pp. 39–98. 10.1007/3-540-29838-x_3.

[119] Mondal J, An JM, Surwase SS, Chakraborty K, Sutradhar SC, Hwang J, Lee J, Lee YK. Carbon nanotube and its derived nanomaterials based high performance biosensing platform. Biosensors (Basel). 2022;12:731. 10.3390/bios12090731. 10.3390/bios12090731 PMC9496036 36140116

[120] Ismail RA, Mohsin MH, Ali AK, Hassoon KI, Erten-Ela S. Preparation and characterization of carbon nanotubes by pulsed laser ablation in water for optoelectronic application. Phys E Low Dimens Syst Nanostruct. 2020;119:1–8. 10.1016/j.physe.2020.113997.

[121] Reza Sadrolhosseini Y-LA, Adzir Mahdi M, Alizadeh F, Abdul Rashid S. Laser ablation technique for synthesis of metal nanoparticle in Liquid’. Laser technology and its applications. London, UK: IntechOpen; 2018. pp. 63–83. 10.5772/intechopen.80374.

[122] Huang L, Zhang Y, Liu X. Dynamics of carbon nanotube-based mode-locking fiber lasers. Nanophotonics. 2020;9:2731–61. 10.1515/nanoph-2020-0269.

[123] Vivekanandhan S, Schreiber M, Muthuramkumar S, Misra M, Mohanty AK. Carbon nanotubes from renewable feedstocks: a move toward sustainable nanofabrication. J Appl Polym Sci. 2017;134. 10.1002/app.44255.

[124] Duraia EM, Opoku M, Beall GW. Efficient eco-friendly synthesis of carbon nanotubes over graphite nanosheets from yellow corn: a one-step green approach. Sci Rep. 2024;14:16405. 10.1038/s41598-024-65893-6. 39013930 10.1038/s41598-024-65893-6 PMC11252411

[125] Lara-Romero J, Ocampo-Macias T, Martínez-Suarez R, Rangel-Segura R, López-Tinoco J, Paraguay-Delgado F, Alonso-Nuñez G, Jiménez-Sandoval S. Chiñas-Castillo, parametric study of the synthesis of carbon nanotubes by spray pyrolysis of a biorenewable feedstock: α-Pinene. ACS Sustain Chem Eng. 2017;5:3890–6. 10.1021/acssuschemeng.6b03054.

[126] Lian JZ, Balapa V, Goetheer E, Cucurachi S. Production of carbon nanotubes from captured carbon: an ex-ante life cycle assessment case study. Chem Eng J. 2024;502:158007. 10.1016/j.cej.2024.158007.

[127] Li H, Ning F, Chen X, Shi A. Effect of carbon and nitrogen double vacancies on the improved photocatalytic hydrogen evolution over porous carbon nitride nanosheets. Catal Sci Technol. 2021;11:3270–8. 10.1039/d0cy02453h.

[128] Alfei S, Zuccari G. Carbon-Nanotube-Based nanocomposites in environmental remediation: an overview of typologies and applications and an analysis of their Paradoxical Double-Sided effects. J Xenobiot. 2025;15:76. 10.3390/jox15030076. 40407540 10.3390/jox15030076 PMC12101166

[129] Zhao D, Zhang Y, Wu C. Adsorption of Pb(II) ions by functionalized multi-walled carbon nanotubes MWCNTs-NH2. J Hazard Mater Adv. 2025;19:100764. 10.1016/j.hazadv.2025.100764.

[130] Kumar S, Bhanjana G, Jangra K, Dilbaghi N, Umar A. Utilization of carbon nanotubes for the removal of Rhodamine B dye from aqueous solutions. J Nanosci Nanotechnol. 2014;14:4331–6. 10.1166/jnn.2014.8077. 24738392 10.1166/jnn.2014.8077

[131] Szlachta M, Wójtowicz P. Adsorption of methylene blue and congo red from aqueous solution by activated carbon and carbon nanotubes. Water Sci Technol. 2013;68:2240–8. 10.2166/wst.2013.487. 10.2166/wst.2013.487 24292474

[132] Ganjoo R, Sharma S, Kumar A, Daouda MMA. Activated carbon: Fundamentals, classification, in: activated carbon. Royal Soc Chem. 2023; pp. 1–22. 10.1039/BK9781839169861-00001.

[133] Jung G, Kim H. Synthesis and photocatalytic performance of PVA/TiO_2_/graphene-MWCNT nanocomposites for dye removal. J Appl Polym Sci. 2014;131:1–7. 10.1002/app.40715.

[134] Jadhav AH, Mai XT, Ofori FA, Kim H. Preparation, characterization, and kinetic study of end opened carbon nanotubes incorporated polyacrylonitrile electrospun nanofibers for the adsorption of pyrene from aqueous solution. Chem Eng J. 2015;259:348–56. 10.1016/j.cej.2014.07.127.

[135] Ma G, Zhu Y, Zhang Z, Li L. Preparation and characterization of multi-walled carbon nanotube/TiO 2 composites: decontamination organic pollutant in water. Appl Surf Sci. 2014;313:817–22. 10.1016/j.apsusc.2014.06.079.

[136] Garcia J, Gomes HT, Serp P, Kalck P, Figueiredo JL, Faria JL. Platinum catalysts supported on MWNT for catalytic wet air oxidation of nitrogen containing compounds. Catal Today. 2005;102–3. 10.1016/j.cattod.2005.02.013.

[137] Frankly RE. The structure of graphitic carbons. Acta Crystallograph. 10.1107/S0365110X51000842.

[138] Ould-Idriss A, Stitou M, Cuerda-Correa EM, Fernández-González C, MacÍas-García A, Alexandre-Franco MF, Gómez-Serrano V. Preparation of activated carbons from olive-tree wood revisited. I. Chemical activation with H3PO4. Fuel Process Technol. 2011;92:261–265. 10.1016/j.fuproc.2010.05.011

[139] Stavropoulos GG, Zabaniotou AA. Production and characterization of activated carbons from olive-seed waste residue. Microporous Mesoporous Mater. 2005;82:79–85. 10.1016/j.micromeso.2005.03.009.

[140] Acharya J, Sahu JN, Mohanty CR, Meikap BC. Removal of lead(II) from wastewater by activated carbon developed from tamarind wood by zinc chloride activation. Chem Eng J. 2009;149:249–62. 10.1016/j.cej.2008.10.029.

[141] Deng H, Li G, Yang H, Tang J, Tang J. Preparation of activated carbons from cotton stalk by microwave assisted KOH and K_2_CO_3_ activation. Chem Eng J. 2010;163:373–81. 10.1016/j.cej.2010.08.019.

[142] Li G, Iakunkov A, Boulanger N, Lazar OA, Enachescu M, Grimm A, Talyzin AV. Activated carbons with extremely high surface area produced from cones, bark and wood using the same procedure. RSC Adv. 2023;13:14543–53. 10.1039/D3RA00820G. 37188252 10.1039/d3ra00820g PMC10177221

[143] Kuyucu AE, Selçuk A, Önal Y, Alacabey İ, Erol K. Effective removal of dyes from aqueous systems by waste-derived carbon adsorbent: physicochemical characterization and adsorption studies. Sci Rep. 2025;15:28835. 10.1038/s41598-025-13685-x. 40770027 10.1038/s41598-025-13685-x PMC12328673

[144] Yao Y, Zuo H, Liu Y, Pang S, Lan L, Yao F, Wu Y, Liu Z. Efficient dye adsorption of mesoporous activated carbon from bamboo parenchyma cells by phosphoric acid activation. RSC Adv. 2024;14:12873–82. 10.1039/D4RA01652A. 38650691 10.1039/d4ra01652a PMC11034359

[145] El maguana Y, Elhadiri N, Benchanaa M, Chikri R. Activated carbon for dyes removal: modeling and understanding the adsorption process. J Chem. 2020;2020:1–9. 10.1155/2020/2096834

[146] Lawtae P, Tangsathitkulchai C. The use of high surface area Mesoporous-Activated carbon from Longan seed biomass for increasing capacity and kinetics of methylene blue adsorption from aqueous solution. Molecules. 2021;26:6521. 10.3390/molecules26216521. 34770928 10.3390/molecules26216521 PMC8587158

[147] Nguyen HD, Tran HN, Chao H, Lin C. Activated carbons derived from Teak Sawdust-Hydrochars for efficient removal of methylene blue, copper, and cadmium from aqueous solution. Water (Basel). 2019;11:2581. 10.3390/w11122581.

[148] Le PT, Bui HT, Le DN, Nguyen TH, Pham LA, Nguyen HN, Nguyen QS, Nguyen TP, Bich NT, Duong TT, Herrmann M, Ouillon S, Le TPQ. Preparation and characterization of biochar derived from agricultural by-products for dye removal. Adsorpt Sci Technol. 2021; 2021. 10.1155/2021/9161904.

[149] Chandrappa R, Das DB. Wastes from industrial and commercial activities. In: Solid waste management: principles and practice. Berlin, Heidelberg: Springer; 2012. pp. 217–47. 10.1007/978-3-642-28681-0_9.

[150] Paul Nayagam JO, Prasanna K. Utilization of shell-based agricultural waste adsorbents for removing dyes: a review. Chemosphere. 2022;291:132737. 10.1016/j.chemosphere.2021.132737. 10.1016/j.chemosphere.2021.132737 34742768

[151] Siddiqui SI, Fatima B, Tara N, Rathi G, Chaudhry SA. Recent advances in remediation of synthetic dyes from wastewaters using sustainable and low-cost adsorbents. Elsevier Ltd.; 2018. 10.1016/B978-0-08-102491-1.00015-0.

[152] Rangabhashiyam S, Anu N, Selvaraju N. Sequestration of dye from textile industry wastewater using agricultural waste products as adsorbents. J Environ Chem Eng. 2013;1:629–41. 10.1016/j.jece.2013.07.014.

[153] Husien S, El-taweel RM, Salim AI, Fahim IS, Said LA, Radwan AG. Review of activated carbon adsorbent material for textile dyes removal: preparation, and modelling. Cur Res Green and Sustain Chem. 2022;5:100325. 10.1016/j.crgsc.2022.100325

[154] Khan N, Tabasi ZA, Liu J, Zhang BH, Zhao Y. Recent advances in functional materials for wastewater treatment: from materials to technological innovations. J Mar Sci Eng. 2022;10:534. 10.3390/jmse10040534.

[155] Sharma YC, Uma SN, Upadhyay. Removal of a cationic dye from wastewaters by adsorption on activated carbon developed from coconut Coir. Energy Fuels. 2009;23:2983–8. 10.1021/ef9001132.

[156] Aljeboree AM, Alkaim AF, Al-Dujaili AH. Adsorption isotherm, kinetic modeling and thermodynamics of crystal Violet dye on coconut husk-based activated carbon. Desalin Water Treat. 2015;53:3656–67. 10.1080/19443994.2013.877854.

[157] Etim UJ, Umoren SA, Eduok UM. Coconut Coir dust as a low cost adsorbent for the removal of cationic dye from aqueous solution. J Saudi Chem Soc. 2016;20:S67–76. 10.1016/j.jscs.2012.09.014.

[158] de Macedo J, da Costa NB, Júnior LE, Almeida EF da, Vieira S, Cestari AR, de Gimenez I, Villarreal NL, Carreño LS, Barreto. Kinetic and calorimetric study of the adsorption of dyes on mesoporous activated carbon prepared from coconut coir dust. J Colloid Interface Sci. 298 (2006) 515–522. 10.1016/j.jcis.2006.01.021 10.1016/j.jcis.2006.01.021 16497318

[159] Aljeboree AM, Alshirifi AN, Alkaim AF. Kinetics and equilibrium study for the adsorption of textile dyes on coconut shell activated carbon. Arab J Chem. 2017;10:S3381–93. 10.1016/j.arabjc.2014.01.020.

[160] Bhatnagar A, Vilar VJP, Botelho CMS, Boaventura RAR. Coconut-based biosorbents for water treatment-a review of the recent literature. Adv Colloid Interface Sci. 2010;160:1–15. 10.1016/j.cis.2010.06.011. 20656282 10.1016/j.cis.2010.06.011

[161] Daoud M, Benturki O, Kecira Z, Girods P, Donnot A. Removal of reactive dye (BEZAKTIV red S-MAX) from aqueous solution by adsorption onto activated carbons prepared from date palm rachis and jujube stones. J Mol Liq. 2017;243:799–809. 10.1016/j.molliq.2017.08.093.

[162] Chowdhury S, Pan S, Balasubramanian R, Das P. Date palm based activated carbon for the efficient removal of organic dyes from aqueous environment. In: Sustainable agriculture reviews 34: date palm for food, medicine and the environment. Cham: Springer International Publishing; 2019. pp. 247–63. 10.1007/978-3-030-11345-2_12

[163] Jabbar NM, Salman SD, Rashid IM, Mahdi YS. Removal of an anionic Eosin dye from aqueous solution using modified activated carbon prepared from date palm fronds. Chem Data Collect. 2022;42:100965. 10.1016/j.cdc.2022.100965.

[164] Alharbi HA, Hameed BH, Alotaibi KD, Aloud SS, Al-Modaihsh AS. Mesoporous activated carbon from leaf sheath date palm fibers by Microwave-Assisted phosphoric acid activation for efficient dye adsorption. ACS Omega. 2022;7:46079–89. 10.1021/acsomega.2c03755. 36570315 10.1021/acsomega.2c03755 PMC9773966

[165] Alharbi HA, Hameed BH, Alotaibi KD, Al-Oud SS, Al-Modaihsh AS. Conversion of a mixture of date palm wastes to mesoporous activated carbon for efficient dye adsorption. Mater Res Express. 2023;10. 10.1088/2053-1591/acb2b6.

[166] Alharbi HA, Hameed BH, Alotaibi KD, Al-Oud SS, Al-Modaihsh AS. Recent methods in the production of activated carbon from date palm residues for the adsorption of textile dyes: a review. Front Environ Sci. 2022;10:1–24. 10.3389/fenvs.2022.996953.

[167] García JR, Sedran U, Zaini MAA, Zakaria ZA. Preparation, characterization, and dye removal study of activated carbon prepared from palm kernel shell. Environ Sci Pollut Res. 2018;25:5076–85. 10.1007/s11356-017-8975-8. 10.1007/s11356-017-8975-8 28391459

[168] Zhi LL, Zaini MAA. Rhodamine B dyes adsorption on palm kernel shell based activated carbons. Malays J Fundamental Appl Sci. 2019;15:743–7. 10.11113/mjfas.v15n5.1396.

[169] Mehr HV, Saffari J, Mohammadi SZ, Shojaei S. The removal of Methyl Violet 2B dye using palm kernel activated carbon: thermodynamic and kinetics model. Int J Environ Sci Technol. 2020;17:1773–82. 10.1007/s13762-019-02271-0.

[170] Xiang VM, Ghazi RM. Adsorption of methylene blue from aqueous solution using palm kernel shell activated carbon. AIP Conf Proc. 2019;2068. 10.1063/1.5089337

[171] Jasri K, Abdulhameed AS, Jawad AH, ALOthman ZA, Yousef TA, Al OK, Duaij. Mesoporous activated carbon produced from mixed wastes of oil palm frond and palm kernel shell using microwave radiation-assisted K_2_CO_3_ activation for methylene blue dye removal: optimization by response surface methodology. Diam Relat Mater. 2023;131:109581. 10.1016/j.diamond.2022.109581.

[172] Ibrahim AS, Ismail KN, Fuadi NA. Review study for activated carbon from palm shell used for treatment of waste water. J Purity Util Reaction Environ. 2012;1:222–36.

[173] Zabi NZ, Ibrahim WNW, Hanapi NSM, Hadzir NM. Removal of various contaminants by highly porous activated carbon sorbent derived from agricultural waste produced in Malaysia - a review. Nat Environ Pollution Technol. 2021;20:1173–83. 10.46488/NEPT.2021.V20I03.025.

[174] Cai Z, Sun Y, Liu W, Pan F, Sun P, Fu J. An overview of nanomaterials applied for removing dyes from wastewater. Environ Sci Pollut Res. 2017;24:15882–904. 10.1007/s11356-017-9003-8. 10.1007/s11356-017-9003-8 28477250

[175] Fawzi Suleiman Khasawneh O, Palaniandy P. Removal of organic pollutants from water by Fe2O3/TiO2 based photocatalytic degradation: a review. Environ Technol Innov. 2021;21:101230. 10.1016/j.eti.2020.101230.

[176] Marimuthu S, Antonisamy AJ, Malayandi S, Rajendran K, Tsai PC, Pugazhendhi A, Ponnusamy VK. Silver nanoparticles in dye effluent treatment: a review on synthesis, treatment methods, mechanisms, photocatalytic degradation, toxic effects and mitigation of toxicity. J Photochem Photobiol B. 2020;205:111823. 10.1016/j.jphotobiol.2020.111823. 32120184 10.1016/j.jphotobiol.2020.111823

[177] Worch E. Adsorption technology in water treatment: fundamentals, processes, and modeling. Berlin, Boston: De Gruyter; 2012. 10.1515/9783110240238.

[178] Madima N, Mishra SB, Inamuddin I, Mishra AK. Carbon-based nanomaterials for remediation of organic and inorganic pollutants from wastewater. a review. Environ Chem Lett. 2020;18:1169–91. 10.1007/s10311-020-01001-0.

[179] Nazal MK. An overview of Carbon-Based materials for the removal of pharmaceutical active Compounds. Carbon-Based Mater Environ Prot Remediat. 2020;1–19. 10.5772/intechopen.91934

[180] Sabzehmeidani MM, Mahnaee S, Ghaedi M, Heidari H, Roy VAL. Carbon based materials: a review of adsorbents for inorganic and organic compounds. Mater Adv. 2021;2:598–627. 10.1039/d0ma00087f.

[181] De Gisi S, Lofrano G, Grassi M, Notarnicola M. Characteristics and adsorption capacities of low-cost sorbents for wastewater treatment: a review. Sustain Mater Technol. 2016;9:10–40. 10.1016/j.susmat.2016.06.002.

[182] Danu BY, Agorku ES, Ampong FK, Awudza JAM, Torve V, Danquah IMK, Ama OM, Osifo PO, Ray SS. Iron sulfide functionalized polyaniline nanocomposite for the removal of Eosin Y from water: equilibrium and kinetic studies. Polym Sci - Ser B. 2021;63:304–13. 10.1134/S1560090421030040.

[183] Danu BY, Agorku ES, Ampong FK, Awudza JAM, Owusu B, Brown PNO, Ama OM, Osifo PO, Ray SS. Chitosan-Graft-Sodium alginate encapsulated vanadium pentoxide (V2 O5) nanocomposite for adsorption and antimicrobial studies. J Adv Mater Sci Eng. 2021;1:1–10. 10.33425/2771-666x.1006.

[184] Adusei JK, Agorku ES, Voegborlo RB, Ampong FK, Awarikabey E, Danu BY, Amarh FA. Zero valent iron impregnated sodium alginate grafted (Acrylamide-Co-Acrylic Acid) adsorbents for the removal of methylene blue in aqueous systems. J Macromolecular Sci Part B: Phys. 2023;62:265–79. 10.1080/00222348.2023.2211376.

[185] Adusei JK, Agorku ES, Voegborlo RB, Ampong FK, Danu BY, Amarh FA. Removal of Methyl red in aqueous systems using synthesized NaAlg-g-CHIT/nZVI adsorbent. Sci Afr. 2022;17:e01273. 10.1016/j.sciaf.2022.e01273.

[186] Foo KY, Hameed BH. An overview of dye removal via activated carbon adsorption process. Desalin Water Treat. 2010;19:255–74. 10.5004/dwt.2010.1214.

[187] Chung J, Sharma N, Kim M, Yun K. Activated carbon derived from sucrose and melamine as low-cost adsorbent with fast adsorption rate for removal of methylene blue in wastewaters. J Water Process Eng. 2022;47:102763. 10.1016/j.jwpe.2022.102763.

[188] Gohr MS, Abd-Elhamid AI, El-Shanshory AA, Soliman HMA. Adsorption of cationic dyes onto chemically modified activated carbon: kinetics and thermodynamic study. J Mol Liq. 2022;346:118227. 10.1016/j.molliq.2021.118227.

[189] Alhogbi BG, Altayeb S, Bahaidarah EA, Zawrah MF. Removal of anionic and cationic dyes from wastewater using activated carbon from palm tree fiber waste. Processes. 2021;9:1–21. 10.3390/pr9030416.

[190] Angin D. Utilization of activated carbon produced from fruit juice industry solid waste for the adsorption of yellow 18 from aqueous solutions. Bioresour Technol. 2014;168:259–66. 10.1016/j.biortech.2014.02.100. 24656549 10.1016/j.biortech.2014.02.100

[191] Wang Y, Pan C, Chu W, Vipin AK, Sun L. Environmental remediation applications of carbon nanotubes and graphene oxide: adsorption and catalysis. Nanomaterials. 2019;9. 10.3390/nano9030439. 10.3390/nano9030439 PMC6474092 30875970

[192] Wu CH. Adsorption of reactive dye onto carbon nanotubes: equilibrium, kinetics and thermodynamics. J Hazard Mater. 2007;144:93–100. 10.1016/j.jhazmat.2006.09.083. 17081687 10.1016/j.jhazmat.2006.09.083

[193] Foroutan R, Peighambardoust SJ, Esvandi Z, Khatooni H, Ramavandi B. Evaluation of two cationic dyes removal from aqueous environments using CNT/MgO/CuFe2O4magnetic composite powder: a comparative study. J Environ Chem Eng. 2021;9:104752. 10.1016/j.jece.2020.104752.

[194] Rajabi M, Mahanpoor K, Moradi O. Removal of dye molecules from aqueous solution by carbon nanotubes and carbon nanotube functional groups: critical review. RSC Adv. 2017;7:47083–90. 10.1039/c7ra09377b.

[195] Ai L, Jiang J. Removal of methylene blue from aqueous solution with self-assembled cylindrical graphene-carbon nanotube hybrid. Chem Eng J. 2012;192:156–63. 10.1016/j.cej.2012.03.056.

[196] Gupta VK, Kumar R, Nayak A, Saleh TA, Barakat MA. Adsorptive removal of dyes from aqueous solution onto carbon nanotubes: a review. Adv Colloid Interface Sci. 2013;193–4. 10.1016/j.cis.2013.03.003. 10.1016/j.cis.2013.03.003 23579224

[197] Cheng ZL, Li YX, Liu Z. Novel adsorption materials based on graphene oxide/Beta zeolite composite materials and their adsorption performance for Rhodamine B. J Alloys Compd. 2017;708:255–63. 10.1016/j.jallcom.2017.03.004.

[198] Chen L, Li Y, Du Q, Wang Z, Xia Y, Yedinak E, Lou J, Ci L. High performance agar/graphene oxide composite aerogel for methylene blue removal. Carbohydr Polym. 2017;155:345–53. 10.1016/j.carbpol.2016.08.047. 27702521 10.1016/j.carbpol.2016.08.047

[199] Abd-Elhamid AI, El-Aassar MR, El Fawal GF, Soliman HMA. Fabrication of polyacrylonitrile/β-cyclodextrin/graphene oxide nanofibers composite as an efficient adsorbent for cationic dye. Environ Nanotechnol Monit Manag. 2019;11:100207. 10.1016/j.enmm.2018.100207.

[200] Yusuf M, Elfghi FM, Zaidic AS, Abdullah EC, Khan AM. Applications of graphene and its derivatives as an adsorbent for heavy metals and dyes removal: a systematic and comprehensive overview. RSC Adv. 2015;5:50392–420.

[201] Kyzas GZ, Deliyanni EA, Bikiaris DN, Mitropoulos AC. Graphene composites as dye adsorbents: review. Chem Eng Res Des. 2018;129:75–88. 10.1016/j.cherd.2017.11.006.

[202] Hu H, Wen W, Ou JZ. Construction of adsorbents with graphene and its derivatives for wastewater treatment: a review. Environ Sci Nano. 2022;9:3226–76. 10.1039/d2en00248e.

[203] Hijab M, Saleem J, Parthasarathy P, Mackey HR, McKay G. Two-stage optimisation for malachite green removal using activated date pits. Biomass Convers Biorefin. 2021;11:727–40. 10.1007/s13399-020-00813-y.

[204] Al-Ghouti MA, Sweleh AO. Optimizing textile dye removal by activated carbon prepared from Olive stones. Environ Technol Innov. 2019;16:100488. 10.1016/j.eti.2019.100488.

[205] Erdogan T, Erdogan FO. Characterization of the adsorption of disperse yellow 211 on activated carbon from Cherry stones following microwave-assisted phosphoric acid treatment. Anal Lett. 2016;49:917–28. 10.1080/00032719.2015.1086776.

[206] Aminu I, Gumel SM, Ahmad WA, Idris AA. Adsorption isotherms and kinetic studies of Congo-Red removal from waste water using activated carbon prepared from jujube seed. Am J Analyt Chem. 2020;11:47–59. 10.4236/ajac.2020.111004.

[207] Kamdod AS, Pavan Kumar MV. Adsorption of methylene blue and Methyl orange on tamarind seed activated carbon and its composite with chitosan: equilibrium and kinetic studies. Desalin Water Treat. 2022;252:408–19. 10.5004/dwt.2022.28270.

[208] Jawad AH, Sauodi MH, Mastuli MS, Aouda MA, Radzun KA. Pomegranate peels collected from fresh juice shop as a renewable precursor for high surface area activated carbon with potential application for methylene blue adsorption. Desalin Water Treat. 2018;124:287–96. 10.5004/dwt.2018.22725.

[209] Şentürk İ, Alzein M. Adsorptive removal of basic blue 41 using pistachio shell adsorbent - performance in batch and column system. Sustain Chem Pharm. 2020;16:100254. 10.1016/j.scp.2020.100254.

[210] Widiyastuti W, Fahrudin Rois M, Suari NMIP, Setyawan H. Activated carbon nanofibers derived from coconut shell charcoal for dye removal application. Adv Powder Technol. 2020;31:3267–73. 10.1016/j.apt.2020.06.012.

[211] Ramutshatsha-Makhwedzha D, Mavhungu A, Moropeng ML, Mbaya R. Activated carbon derived from waste orange and lemon peels for the adsorption of methyl orange and methylene blue dyes from wastewater. Heliyon. 2022;8:e09930. 10.1016/j.heliyon.2022.e09930. 35965978 10.1016/j.heliyon.2022.e09930 PMC9363969

[212] Auta M, Hameed BH. Preparation of waste tea activated carbon using potassium acetate as an activating agent for adsorption of acid blue 25 dye. Chem Eng J. 2011;171:502–9. 10.1016/j.cej.2011.04.017.

[213] Yazdan MMS, Kumar R, Leung SW. The environmental and health impacts of steroids and hormones in wastewater effluent. as well as existing removal technologies: a review. Ecologies. 2022;3:206–24. 10.3390/ecologies3020016

[214] Sukeesan S, Boontanon SK, Boontanon N, Fujii S. Regeneration of Ion-Exchange resins and granular activated carbon with the sonochemical technique for enabling adsorption of aqueous Per- and polyfluoroalkyl substances. IOP Conf Ser Earth Environ Sci. 2022;973:12004. 10.1088/1755-1315/973/1/012004.

[215] da Santos DH, Xiao Y, Chaukura N, Hill JM, Selvasembian R, Zanta CLPS, Meili L. Regeneration of dye-saturated activated carbon through advanced oxidative processes: a review. Heliyon. 2022;8. 10.1016/j.heliyon.2022.e10205. 10.1016/j.heliyon.2022.e10205 PMC9404357 36033294

[216] Larasati A, Fowler GD, Graham NJD. Insights into chemical regeneration of activated carbon for water treatment. J Environ Chem Eng. 2021;9:105555. 10.1016/j.jece.2021.105555.

[217] Alcalde-Garcia F, Prasher S, Kaliaguine S, Tavares JR, Dumont M-J. Desorption strategies and reusability of biopolymeric adsorbents and semisynthetic derivatives in hydrogel and hydrogel composites used in adsorption processes. ACS Eng Au. 2023;3:443–60. 10.1021/acsengineeringau.3c00022.

[218] Durán-Jiménez G, Stevens LA, Hodgins GR, Uguna J, Ryan J, Binner ER, Robinson JP. Fast regeneration of activated carbons saturated with textile dyes: textural, thermal and dielectric characterization. Chem Eng J. 2019;378:121774. 10.1016/j.cej.2019.05.135.

[219] Messaoudi NE, Mohammed E, Khomri, · Abdelaziz E, Mouden A, Bouich, · Amane Jada A, Lacherai, · Hafiz MN, Iqbal, · Sikandar I, Mulla V, Kumar J, Heloisa P. Américo-Pinheiro, Regeneration and reusability of non-conventional low-cost adsorbents to remove dyes from wastewaters in multiple consecutive adsorption–desorption cycles: a review. Biomass Conv. Bioref. 2024;14:11739–11756. 10.1007/s13399-022-03604-9

[220] Durán-Jiménez G, Stevens LA, Hodgins GR, Uguna J, Ryan J, Binner ER, Robinson JP. Fast regeneration of activated carbons saturated with textile dyes: textural, thermal and dielectric characterization. Chem Eng J. 2019;378. 10.1016/j.cej.2019.05.135.

[221] da Silva Santos DH, Xiao Y, Chaukura N, Hill JM, Selvasembian R, Zanta CLPS, Meili L. Regeneration of dye-saturated activated carbon through advanced oxidative processes: a review. Heliyon. 2022;8:e10205. 10.1016/j.heliyon.2022.e10205 10.1016/j.heliyon.2022.e10205 PMC9404357 36033294

[222] Ceroni L, Benazzato S, Pressi S, Calvillo L, Marotta E, Menna E. Enhanced adsorption of methylene blue dye on functionalized Multi-walled carbon nanotubes. nanomaterials. 2024;14:522. 10.3390/nano14060522 10.3390/nano14060522 PMC10974461 38535671

[223] He Q, Qi J, Liu X, Zhang H, Wang Y, Wang W, Guo F. Carbon-in-Silicate nanohybrid constructed by in situ confined conversion of organics in rectorite for complete removal of dye from water. Nanomaterials. 2023;13:2627. 10.3390/nano13192627. 37836268 10.3390/nano13192627 PMC10574537

[224] Hossain MR, Rashid TU, Lata NP, Dey SC, Sarker M, Shamsuddin SM. Fabrication of novel nanohybrid material for the removal of Azo dyes from wastewater. J Compos Sci. 2022;6. 10.3390/jcs6100304.

[225] George G, Ealias AM, Saravanakumar MP. Advancements in textile dye removal: a critical review of layered double hydroxides and clay minerals as efficient adsorbents. Environ Sci Pollut Res. 2024;31:12748–79. 10.1007/s11356-024-32021-w. 10.1007/s11356-024-32021-w 38265587

[226] Kurniawan TA, Sillanpää MET, Sillanpää M. Nanoadsorbents for remediation of aquatic environment: local and practical solutions for global water pollution problems. Crit Rev Environ Sci Technol. 2012;42:1233–95. 10.1080/10643389.2011.556553.

[227] Sheoran K, Kaur H, Siwal SS, Saini AK, Vo DVN, Thakur VK. Recent advances of carbon-based nanomaterials (CBNMs) for wastewater treatment: synthesis and application. Chemosphere. 2022;299. 10.1016/j.chemosphere.2022.134364 10.1016/j.chemosphere.2022.134364 35318024

[228] Gao M, Peh CK, Meng FL, Ho GW. Photothermal membrane distillation toward solar water production. Small Methods. 2021;5:1–17. 10.1002/smtd.202001200 10.1002/smtd.202001200 34928082

[229] Liu Z, Ling Q, Cai Y, Xu L, Su J, Yu K, Wu X, Xu J, Hu B, Wang X. Synthesis of carbon-based nanomaterials and their application in pollution management. Nanoscale Adv. 2022;4:1246–62. 10.1039/d1na00843a. 36133685 10.1039/d1na00843a PMC9419251

[230] Lu F, Astruc D. Nanocatalysts and other nanomaterials for water remediation from organic pollutants. Coord Chem Rev. 2020;408:213180. 10.1016/j.ccr.2020.213180.

[231] Porcu S, Secci F, Ricci PC. Advances in hybrid composites for photocatalytic applications: a review. Molecules. 2022;27:6828. 10.3390/molecules27206828 10.3390/molecules27206828 PMC9607189 36296421

[232] Jia H, He W, Wamer WG, Han X, Zhang B, Zhang S, Zheng Z, Xiang Y, Yin JJ. Generation of reactive oxygen species, electrons/holes, and photocatalytic degradation of Rhodamine b by photoexcited cds and Ag2S micro-nano structures. J Phys Chem C. 2014;118:21447–56. 10.1021/jp505783y.

[233] Rasool MA, Sattar R, Anum A, Al-Hussain SA, Ahmad S, Irfan A, Zaki MEA. An insight into carbon Nanomaterial-Based photocatalytic water splitting for green hydrogen production. Catalysts. 2023;13:1–31. 10.3390/catal13010066.

[234] Ismail AA, Ali AM, Harraz FA, Faisal M, Shoukry H, Al-Salami AE. A facile synthesis of α-Fe2O3/carbon nanotubes and their photocatalytic and electrochemical sensing performances. Int J Electrochem Sci. 2019;14:15–32. 10.20964/2019.01.09.

[235] Yu L, Xu W, Liu H, Bao Y. Titanium dioxide–reduced graphene oxide composites for photocatalytic degradation of dyes in Water. Catalysts. 2022;12:1340. 10.3390/catal12111340

[236] Chakhtouna H, Zari N, Bouhfid R, kacem Qaiss A, Benzeid H. Novel photocatalyst based on date palm fibers for efficient dyes removal. J Water Process Eng. 2021;43:102167. 10.1016/j.jwpe.2021.102167

[237] Leelavathi H, Muralidharan R, Abirami N, Tamizharasan S, Sankeetha S, Kumarasamy A, Arulmozhi R. Construction of step-scheme g-C_3_N_4_/Co/ZnO heterojunction photocatalyst for aerobic photocatalitic degradation of synthetic wastewater. Colloids Surf Physicochem Eng Asp. 2023;656:130449. 10.1016/j.colsurfa.2022.130449.

[238] Abdel-Fatah Alkahlawy A, Abbas El-Salamony R, Gobara HM. Photocatalytic degradation of congo red dye via multi-walled carbon nanotubes modified Cuo and Zno nanoparticles under visible light irradiation. Egypt J Chem. 2021;64:1481–94. 10.21608/EJCHEM.2020.47684.2985.

[239] Zhao G, Zhang D, Yu J, Xie Y, Hu W, Jiao F. Multi-walled carbon nanotubes modified Bi2S3 microspheres for enhanced photocatalytic decomposition efficiency. Ceram Int. 2017;43:15080–8. 10.1016/j.ceramint.2017.08.036.

[240] Maruthupandy M, Qin P, Muneeswaran T, Rajivgandhi G, Quero F, Song J-M. Graphene-zinc oxide nanocomposites (G-ZnO NCs): Synthesis, characterization and their photocatalytic degradation of dye molecules. Mater Sci Engineering: B. 2020;254:114516. 10.1016/j.mseb.2020.114516.

[241] Kuila SK, Sarkar R, Kumbhakar P, Kumbhakar P, Tiwary CS, Kundu TK. Photocatalytic dye degradation under sunlight irradiation using cerium ion adsorbed two-dimensional graphitic carbon nitride. J Environ Chem Eng. 2020;8:103942. 10.1016/j.jece.2020.103942.

[242] Raizada P, Aslam Parwaz Khan A, Singh P. Construction of carbon nanotube mediated Fe doped graphitic carbon nitride and Ag3VO4 based Z-scheme heterojunction for H2O2 assisted 2,4 dimethyl phenol photodegradation. Sep Purif Technol. 2020;247:116957. 10.1016/j.seppur.2020.116957.

[243] Gao Z-Z, Qi N, Chen W-J, Zhao H. Construction of hydroxyethyl cellulose/silica/graphitic carbon nitride solid foam for adsorption and photocatalytic degradation of dyes. Arab J Chem. 2022;15:104105. 10.1016/j.arabjc.2022.104105.

[244] Loo WW, Pang YL, Lim S, Wong KH, Lai CW, Abdullah AZ. Enhancement of photocatalytic degradation of malachite green using iron doped titanium dioxide loaded on oil palm empty fruit bunch-derived activated carbon. Chemosphere. 2021;272:129588. 10.1016/j.chemosphere.2021.129588. 33482519 10.1016/j.chemosphere.2021.129588

[245] Kamaraj M, Srinivasan NR, Assefa G, Adugna AT, Kebede M. Facile development of sunlit ZnO nanoparticles-activated carbon hybrid from pernicious weed as an operative nano-adsorbent for removal of methylene blue and chromium from aqueous solution: extended application in tannery industrial wastewater. Environ Technol Innov. 2020;17:100540. 10.1016/j.eti.2019.100540.

[246] Sapkota KP, Lee I, Hanif MA, Islam MA, Hahn JR. Solar-light-driven efficient ZnO–Single-Walled carbon nanotube photocatalyst for the degradation of a persistent water pollutant organic dye. Catalysts. 2019;9:498. 10.3390/catal9060498.

[247] Kuvarega AT, Mamba BB. Double walled carbon nanotube/TiO_2_ nanocomposites for photocatalytic dye degradation. J Nanomater. 2016;1–9. 10.1155/2016/3746861

[248] Panahian Y, Arsalani N. Synthesis of hedgehoglike F-TiO2(B)/CNT nanocomposites for sonophotocatalytic and photocatalytic degradation of malachite green (MG) under visible light: kinetic study. J Phys Chem A. 2017;121:5614–24. 10.1021/acs.jpca.7b02580. 28691495 10.1021/acs.jpca.7b02580

[249] Anis SF, Hashaikeh R, Hilal N. Microfiltration membrane processes: a review of research trends over the past decade. J Water Process Eng. 2019;32. 10.1016/j.jwpe.2019.100941.

[250] Sagle A, Freeman B. Fundamentals of membranes for water treatment. The future of desalination in Texas. Austin, TX: Texas Water Development Board. 2004;363:137–54.

[251] Carroll T, King S, Gray SR, Bolto BA, Booker NA. The fouling of microfiltration membranes by NOM after coagulation treatment. Water Res. 2000;34. 10.1016/S0043-1354(00)00051-8.

[252] Zhang X, Zhang B, Wu Y, Wang T, Qiu J. Preparation and characterization of a diatomite hybrid microfiltration carbon membrane for oily wastewater treatment. J Taiwan Inst Chem Eng. 2018;89:39–48. 10.1016/j.jtice.2018.04.035.

[253] Homem NC, Beluci NdeCL, Amorim S, Reis R, Vieira AMS, Vieira MF, Bergamasco R, Amorim MTP. Surface modification of a polyethersulfone microfiltration membrane with graphene oxide for reactive dyes removal. Appl Surf Sci. 2019;486:499–507. 10.1016/j.apsusc.2019.04.276.

[254] Al Aani S, Mustafa TN, Hilal N. Ultrafiltration membranes for wastewater and water process engineering: a comprehensive statistical review over the past decade. J Water Process Eng. 2020;35. 10.1016/j.jwpe.2020.101241.

[255] Ahmad T, Guria C, Mandal A. A review of oily wastewater treatment using ultrafiltration membrane: a parametric study to enhance the membrane performance. J Water Process Eng. 2020;36. 10.1016/j.jwpe.2020.101289.

[256] Athanasekou CP, Morales-Torres S, Likodimos V, Romanos GE, Pastrana-Martinez LM, Falaras P, Faria JL, Figueiredo JL, Silva AMT. Prototype composite membranes of partially reduced graphene oxide/TiO2 for photocatalytic ultrafiltration water treatment under visible light. Appl Catal B. 2014;158–9. 10.1016/j.apcatb.2014.04.012.

[257] Fan Q, Cheng X, Zhu X, Luo C, Ren H, Wu D, Liang H. Secondary wastewater treatment using peroxymonosulfate activated by a carbon nanofiber supported Co3O4 (Co3O4@CNF) catalyst combined with ultrafiltration. Sep Purif Technol. 2022;287:120579. 10.1016/j.seppur.2022.120579.

[258] Oduro AA, Biney SAB, Nortey ES, Kangmennaa A, Forkuo RB, Agorku ES. Polyvinyl alcohol embedded activated Clay/Hydroxyapatite membranes for fouling control during the removal of an organic dye in water. J Appl Polym Sci. 2025;1–14. 10.1002/app.56701.

[259] Joo SH, Tansel B. Novel technologies for reverse osmosis concentrate treatment: a review. J Environ Manage. 2015;150. 10.1016/j.jenvman.2014.10.027. 10.1016/j.jenvman.2014.10.027 25528173

[260] Wenten IG, Khoiruddin. Reverse osmosis applications: prospect and challenges. Desalination. 2016;391. 10.1016/j.desal.2015.12.011.

[261] Inukai S, Cruz-Silva R, Ortiz-Medina J, Morelos-Gomez A, Takeuchi K, Hayashi T, Tanioka A, Araki T, Tejima S, Noguchi T, Terrones M, Endo M. High-performance multi-functional reverse osmosis membranes obtained by carbon nanotube·polyamide nanocomposite. Sci Rep. 2015;5:1–10. 10.1038/srep13562. 10.1038/srep13562 PMC4558580 26333385

[262] Mohammad AW, Teow YH, Ang WL, Chung YT, Oatley-Radcliffe DL, Hilal N. Nanofiltration membranes review: recent advances and future prospects. Desalination. 2015;356. 10.1016/j.desal.2014.10.043.

[263] Hilal N, Al-Zoubi H, Darwish NA, Mohammad AW, Arabi MA. A comprehensive review of nanofiltration membranes: treatment, pretreatment, modelling, and atomic force microscopy. Desalination. 2004;170. 10.1016/j.desal.2004.01.007.

[264] Ji Y, Qian W, Yu Y, An Q, Liu L, Zhou Y, Gao C. Recent developments in nanofiltration membranes based on nanomaterials. Chin J Chem Eng. 2017;25. 10.1016/j.cjche.2017.04.014.

[265] Wang L, Song X, Wang T, Wang S, Wang Z, Gao C. Fabrication and characterization of polyethersulfone/carbon nanotubes (PES/CNTs) based mixed matrix membranes (MMMs) for nanofiltration application. Appl Surf Sci. 2015;330:118–25. 10.1016/j.apsusc.2014.12.183.

[266] Misenan MSM, Farabi MSA, Akhlisah ZN, Khiar ASA. Enhancing polymer electrolytes with carbon nanotube fillers: a promising frontier. Next Mater. 2025;7:100365. 10.1016/j.nxmate.2024.100365.

[267] Saleem A, Iqbal R, Hussain A, Javed MS, Ashfaq MZ, Imran M, Hussain MM, Akbar AR, Jun S, Majeed MK. Recent advances and perspectives in carbon-based fillers reinforced Si3N4 composite for high power electronic devices. Ceram Int. 2022;48:13401–19. 10.1016/j.ceramint.2022.02.050.

[268] Huang Y, Kormakov S, He X, Gao X, Zheng X, Liu Y, Sun J, Wu D. Conductive polymer composites from renewable resources: an overview of preparation, properties, and applications. Polymers (Basel). 2019;11:1–32. 10.3390/polym11020187 10.3390/polym11020187 PMC6418900 30960171

[269] Regmi C, Kshetri YK, Wickramasinghe SR. Carbon-Based nanocomposite membranes for membrane distillation: progress, problems and future prospects. Membranes (Basel). 2024;14. 10.3390/membranes14070160 10.3390/membranes14070160 PMC11278710 39057668

[270] Gill YQ, Jin J, Song M. Comparative study of carbon-based nanofillers for improving the properties of HDPE for potential applications in food tray packaging. Polym Polym Compos. 2020;28:562–71. 10.1177/0967391119892091.

[271] Noamani S, Niroomand S, Rastgar M, Sadrzadeh M. Carbon-based polymer nanocomposite membranes for oily wastewater treatment. NPJ Clean Water. 2019;2:1–14. 10.1038/s41545-019-0044-z.

[272] Shafi’i AM, Ma’aruf A, Muhammad M, Sulaiman SR, Abdullahi A, Musa Shuaibu I, Abdurrashid. Carbon-Based Hybrid Mater Water Treatment: Compr Insights Appl. 2024;2:90–119. www.theacademic.in.

[273] Nasrollahzadeh M, Sajjadi M, Iravani S, Varma RS. Carbon-based sustainable nanomaterials for water treatment: state-of-art and future perspectives. Chemosphere. 2021;263:128005. 10.1016/j.chemosphere.2020.128005. 33297038 10.1016/j.chemosphere.2020.128005 PMC7880008

[274] Nazal MK. An Overview of Carbon-based materials for the removal of pharmaceutical active compounds. In: Carbon-based material for environmental protection and remediation. In: Bartoli M, Frediani M, Rosi L, editors. London, UK: IntechOpen; 2020. pp. 83–101. 10.5772/intechopen.91934.

[275] Piaskowski K, Świderska-Dąbrowska R, Dąbrowski T. Carbon-Based nanomaterials in water and wastewater treatment processes. Sustainability. 2025;17:7414. 10.3390/su17167414.

[276] Hamda AS, Areti HA, Gudeta RL, Abo LD, Jayakumar M. Carbon-based nanomaterials for water treatment: a comprehensive review of recent advances and mechanisms. Chem Eng J Adv. 2025;23:100834. 10.1016/j.ceja.2025.100834.

[277] Khademolqorani S, Shah KJ, Dilamian M, Osman AI, Altiok E, Azizi S, Kamika I, Yang Y, Yalcinkaya F, Yvaz A, Banitaba SN. Innovative integration of layered carbon materials in biopolymer fibrous membranes for sustainable water treatment. Adv Sustain Syst. 2025;9. 10.1002/adsu.202500035.

[278] Wang Y, Chen L, Zhu Y, Fang W, Tan Y, He Z, Liao H. Research status, trends, and mechanisms of Biochar adsorption for wastewater treatment: a scientometric review. Environ Sci Eur. 2024;36:25. 10.1186/s12302-024-00859-z.

[279] Nidheesh PV, Kumar M, Venkateshwaran G, Ambika S, Bhaskar S, Vinay P, Ghosh. Conversion of locally available materials to Biochar and activated carbon for drinking water treatment. Chemosphere. 2024;353:141566. 10.1016/j.chemosphere.2024.141566. 38428536 10.1016/j.chemosphere.2024.141566

[280] Dey D, Paul S, Banerjee P. Chap. 27 - Wastewater treatments using carbon nanotubes: recent developments. In: C. Verma, J. Aslam, M.E. Khan, editors. Adsorption through Advanced nanoscale materials. Elsevier, 2023: pp. 647–679. 10.1016/B978-0-443-18456-7.00027-4

[281] Prasser DR. startus-insights.com, Discover the top 10 water treatment trends in 2025. (2025). https://www.startus-insights.com/innovators-guide/water-treatment-trends/ (accessed July 20, 2025).

[282] Rani S, Sabharwal H, Kumar P, Chauhan AK, Khoo KS, Kataria N. Comparative behavior of carbon-based materials for the removal of emerging bisphenol A from water: adsorption modelling and mechanism. Groundw Sustain Dev. 2024;25:101121. 10.1016/j.gsd.2024.101121.

[283] Parameswaranpillai J, Das P, Ganguly S, Chavali M, Hameed N. Polymer-Carbonaceous filler based composites for wastewater treatment. Boca Raton: CRC 2023. 10.1201/9781003328094.

[284] Bhushan B, Negi P, Nayak A, Goyal S. Graphene composites for water remediation: an overview of their advanced performance with focus on challenges and future prospects. Adv Compos Hybrid Mater. 2024;8:55. 10.1007/s42114-024-01088-x.

[285] Selvaraj M, Hai A, Banat F, Haija MA. Application and prospects of carbon nanostructured materials in water treatment: a review. J Water Process Eng. 2020;33:100996. 10.1016/j.jwpe.2019.100996.

[286] Mbugua JK, Nyambura Mbugua M, Mumbua Mbugua L, Andati GW. The role of Biochar in sustainable wastewater management in Kenya: current practices, challenges, and future prospects. J Environ Ecol. 2025;1:10–6. 10.64229/c4qpb212.

[287] Mohammed S, Fatumah N, Abasi K, Olupot M, Egesa M, Rubhara T, Augustyniak A, O’Connor T, Tsolakis N, Gaffey J, McMahon H, Anastasiadis F. Co-designing sustainable Biochar business models with sub-Saharan African communities for inclusive socio-economic transformation. Sci Rep. 2024;14:15802. 10.1038/s41598-024-66120-y. 38982126 10.1038/s41598-024-66120-y PMC11233699

[288] Muzioreva H, Gumbo T, Kavishe N, Moyo T, Musonda I. Decentralized wastewater system practices in developing countries: a systematic review. Util Policy. 2022;79:101442. 10.1016/j.jup.2022.101442.

[289] Garrido-Baserba M, Sedlak DL, Molinos-Senante M, Barnosell I, Schraa O, Rosso D, Verdaguer M, Poch M. Using water and wastewater decentralization to enhance the resilience and sustainability of cities. Nat Water. 2024;2:953–74. 10.1038/s44221-024-00303-9.

[290] Zango ZU, Garba A, Haruna A, Imam SS, Katsina AU, Ali AF, Abidin AZ, Zango MU, Garba ZN, Hosseini-Bandegharaei A, Yuguda AU, Adamu H. A systematic review on applications of biochar and activated carbon derived from biomass as adsorbents for sustainable remediation of antibiotics from pharmaceutical wastewater. J Water Process Eng. 2024;67:106186. 10.1016/j.jwpe.2024.106186.

[291] Soffian MS, Abdul Halim FZ, Aziz F, A.Rahman M, Mohamed Amin MA, Awang DN, Chee. Carbon-based material derived from biomass waste for wastewater treatment. Environ Adv. 2022;9:100259. 10.1016/j.envadv.2022.100259.

[292] Kamali M, Appels L, Kwon EE, Aminabhavi TM, Dewil R. Biochar in water and wastewater treatment - a sustainability assessment. Chem Eng J. 2021;420:129946. 10.1016/j.cej.2021.129946.

[293] Anuwa-Amarh NA, Dizbay-Onat M, Venkiteshwaran K, Wu S. Carbon-Based adsorbents for microplastic removal from wastewater. Materials. 2024;17. 10.3390/ma17225428. 10.3390/ma17225428 PMC11595638 39597251

[294] Yagub MT, Sen TK, Afroze S, Ang HM. Dye and its removal from aqueous solution by adsorption: a review. Adv Colloid Interface Sci. 2014;209:172–84. 10.1016/j.cis.2014.04.002. 24780401 10.1016/j.cis.2014.04.002

[295] Bridges T, Jones S, Shudtz M. Innovating through Nature-Positive engineering: how can we move forward? Int J Mar Coastal Law. 2024;39:541–50. 10.1163/15718085-bja10197.

[296] Karczmarczyk A, Bus A, Kaczor G. Adapting wastewater infrastructure to the new EU directive: modernization challenges and environmental benefits – Masovian voivodship case study. Desalin Water Treat. 2025;323:101286. 10.1016/j.dwt.2025.101286.

[297] Dassanayake RS, Acharya S, Abidi N. Recent advances in Biopolymer-Based dye removal technologies. Molecules. 2021;26. 10.3390/molecules26154697. 10.3390/molecules26154697 PMC8347927 34361855

[298] Bulgariu L, Escudero LB, Bello OS, Iqbal M, Nisar J, Adegoke KA, Alakhras F, Kornaros M, Anastopoulos I. The utilization of leaf-based adsorbents for dyes removal: a review. J Mol Liq. 2019;276:728–47. 10.1016/j.molliq.2018.12.001.

[299] Akhtar MS, Ali S, Zaman W. Innovative adsorbents for pollutant removal: exploring the latest research and applications. Molecules. 2024;29. 10.3390/molecules29184317. 10.3390/molecules29184317 PMC11433758 39339312

[300] Nahurskyi N, Malovanyy M, Bordun I, Szymczykiewicz E. Magnetically sensitive Carbon-Based nanocomposites for the removal of dyes and heavy metals from wastewater: a review. Chem Chem Technol. 2024;18:170–87. 10.23939/chcht18.02.170

[301] Bazan-Wozniak A, Wolski R, Paluch D, Nowicki P, Pietrzak R. Removal of organic dyes from aqueous solutions by activated carbons prepared from residue of supercritical extraction of marigold. Materials. 2022;15:3655. 10.3390/ma15103655. 35629683 10.3390/ma15103655 PMC9143942

[302] Aragaw TA, Bogale FM. Biomass-Based adsorbents for removal of dyes from wastewater: a review. Front Environ Sci. 2021;9. 10.3389/fenvs.2021.764958.

[303] Sriram G, Dhanabalan K, Vishwanath RS, Oh TH. Progress in porous carbon-based adsorbents for the removal of dyes from single and binary systems and their adsorption mechanism – a review. Inorg Chem Front. 2025. 10.1039/D5QI00952A.

[304] Isaeva VI, Vedenyapina MD, Kurmysheva AY, Weichgrebe D, Nair RR, Nguyen NPT, Kustov LM. Modern Carbon–Based materials for adsorptive removal of organic and inorganic pollutants from water and wastewater. Molecules. 2021;26:6628. 10.3390/molecules26216628. 34771037 10.3390/molecules26216628 PMC8587771

[305] Moteallemi A, Taherkhani S, Ahmadfazeli A, Dehghani MH. A systematic review of plastic wastes as new adsorbents for dye removal in aqueous environments. Environ Sci Eur. 2025;37:115. 10.1186/s12302-025-01172-z.

[306] Belda Marín C, Egles C, Landoulsi J, Guénin E. Silk bionanocomposites for organic dye absorption and degradation. Appl Sci. 2022;12:9152. 10.3390/app12189152.

[307] Khan S, Noor T, Iqbal N, Yaqoob L. Photocatalytic dye degradation from textile wastewater: a review. ACS Omega. 2024;9:21751–67. 10.1021/acsomega.4c00887. 38799325 10.1021/acsomega.4c00887 PMC11112581

[308] Alamgir N, Mushtaq A, Ahmad JKE, Erum L, Li J, Qian X, Wang J, Gao. Shaping the future of solar-driven photocatalysis by reticular framework materials. J Mater Sci Technol. 2025;231:193–244. 10.1016/j.jmst.2025.02.009.

